# On the critical competition between singlet exciton decay and free charge generation in non-fullerene based organic solar cells with low energetic offsets†

**DOI:** 10.1039/d4ee01409j

**Published:** 2024-07-30

**Authors:** Manasi Pranav, Atul Shukla, David Moser, Julia Rumeney, Wenlan Liu, Rong Wang, Bowen Sun, Sander Smeets, Nurlan Tokmoldin, Yonglin Cao, Guorui He, Thorben Beitz, Frank Jaiser, Thomas Hultzsch, Safa Shoaee, Wouter Maes, Larry Lüer, Christoph Brabec, Koen Vandewal, Denis Andrienko, Sabine Ludwigs, Dieter Neher

**Affiliations:** a Institute of Physics and Astronomy, University of Potsdam, Karl-Liebknecht Straße 24/25 14476 Potsdam Germany neher@uni-potsdam.de; b IPOC – Functional Polymers, Institute of Polymer Chemistry, University of Stuttgart, Pfaffenwaldring 55 70569 Stuttgart Germany; c Max Planck Institute for Polymer Research, Ackermannweg 10 55128 Mainz Germany; d Institute of Materials for Electronics and Energy Technology (i-MEET), Friedrich-Alexander-Universität Erlangen-Nürnberg, Martensstrasse 7 Erlangen 91058 Germany; e UHasselt—Hasselt University, Institute for Materials Research, (IMO-IMOMEC), Agoralaan 1 3590 Diepenbeek Belgium; f IMOMEC Division, IMEC, Wetenschapspark 1 3590 Diepenbeek Belgium; g Heterostructure Semiconductor Physics, Paul-Drude-Institut für Festkörperelektronik, Leibniz-Institut im Forschungsverbund Berlin e. V, Hausvogteiplatz 5-7 10117 Berlin Germany; h Helmholtz-Institut Erlangen-Nürnberg for Renewable Energies (HIERN), Forschungszentrum Jülich, Immerwahrstraße 2 91058 Erlangen Germany

## Abstract

Reducing voltage losses while maintaining high photocurrents is the holy grail of current research on non-fullerene acceptor (NFA) based organic solar cell. Recent focus lies in understanding the various fundamental mechanisms in organic blends with minimal energy offsets – particularly the relationship between ionization energy offset (ΔIE) and free charge generation. Here, we quantitatively probe this relationship in multiple NFA-based blends by mixing Y-series NFAs with PM6 of different molecular weights, covering a broad power conversion efficiency (PCE) range: from 15% down to 1%. Spectroelectrochemistry reveals that a ΔIE of more than 0.3 eV is necessary for efficient photocurrent generation. Bias-dependent time-delayed collection experiments reveal a very pronounced field-dependence of free charge generation for small ΔIE blends, which is mirrored by a strong and simultaneous field-dependence of the quantified photoluminescence from the NFA local singlet exciton (LE). We find that the decay of singlet excitons is the primary competition to free charge generation in low-offset NFA-based organic solar cells, with neither noticeable losses from charge-transfer (CT) decay nor evidence for LE–CT hybridization. In agreement with this conclusion, transient absorption spectroscopy consistently reveals that a smaller ΔIE slows the NFA exciton dissociation into free charges, albeit restorable by an electric field. Our experimental data align with Marcus theory calculations, supported by density functional theory simulations, for zero-field free charge generation and exciton decay efficiencies. We conclude that efficient photocurrent generation generally requires that the CT state is located below the LE, but that this restriction is lifted in systems with a small reorganization energy for charge transfer.

## Abstract 2

A quantitative study, supported by Marcus theory and DFT, showing why the fate of singlet excitons is the pivot to free charge generation in low-energy offset organic solar cells.

## Graphical abstract

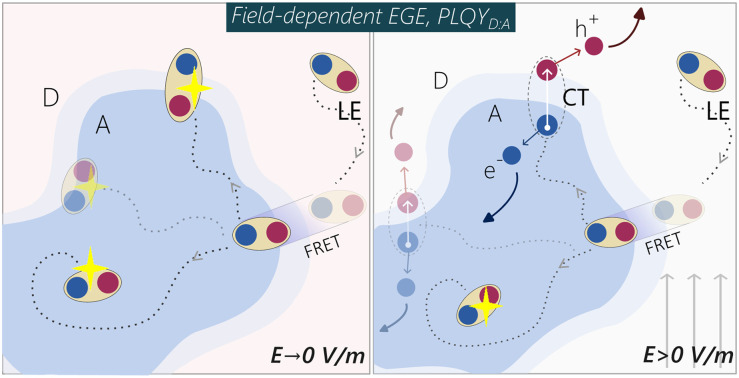

**Broader context**

Within the organic solar cell research community, there is consensus that a major voltage loss arises from the necessary energy offset between the blend constituents at the donor:acceptor (D:NFA) heterojunction. Therefore, recent efforts aim to increase the device efficiency through the reduction of the interfacial energy offset in D:NFA blends. However, the offset must be large enough to drive the dissociation of initially photogenerated local singlet excitons into free charge. Agreeably, low-offset systems with insufficient driving force suffer from small fill factors and low, often bias-dependent, photocurrents. As causes for this, field-dependent exciton dissociation, inefficient charge-transfer separation as well as the hybridization of the singlet state with the interfacial charge-transfer state have been proposed in the past. Given the great importance of studying the underlying mechanisms in such low-offset blends, we herein quantitatively assess the free charge generation and emission properties on a sample set of Y-series based blends in which the energetic driving force and the resultant device performance were methodically tuned. Thereby, we correlate experimental observations from several optoelectronic and spectroscopic techniques at various time-scales with Marcus theory simulations supporting by quantum mechanical modelling. We closely examine the processes most critical to free charge generation and, thereby, generate a model of free-charge generation limited by LE decay on the NFA acceptor domains. This is found to generally and accurately describe the generation and emission properties of several D:NFA blends. In broader terms, our findings suggests a lower limit to the energetic offset at the D:NFA interface for efficient free charge generation, which describes the boundary within which the energetics of several reported state-of-the-art single-junction blends are found to lie. Ultimately, our findings provide comprehensive views on the energetic offset regimes that are of most interest to circumvent loss pathways during free charge generation.

## Introduction

Organic solar cells (OSCs) have witnessed remarkable improvement in performance since the advent of low-bandgap non-fullerene acceptors (NFAs). When blended with an appropriate electron donor (D) to form a bulk heterojunction (BHJ), state-of-the-art NFA-based OSCs nowadays match their inorganic and perovskite competitors in terms of high short-circuit current densities (*J*_SC_) and fill factors (FF), but lag behind with respect to their open circuit voltage (*V*_OC_).^1–4^ This is in part due to the need of an energy offset between the blend constituents at the D:A heterojunction, which must be large enough to dissociate the initially photogenerated local singlet exciton (LE) into an interfacial charge-transfer (CT) state and eventually into free charge carriers. Since in most state-of-the-art OSCs the difference of the ionisation energies (ΔIE) is smaller than that of the electron affinities (ΔEA), the ΔIE critically determines voltage losses. In practice, ΔIE of the neat components is often used as a first approximation of the LE–CT energetic offset; however, this simplification neglects other contributions to the offset, such as the difference of the LE and CT binding energies,^5^ the electrostatics at the D:A interfaces,^6^ or differences in the molecular packing and conformation in the blend and especially at the D:A interface.^7,8^

In organic solar cells, photocurrent generation comprises several steps. The first is the dissociation of the initially formed LE to form the interfacial CT state. This is also referred to as charge generation. The next step is the split-up of the CT state into a pair of independent unbound charge carriers, which is called charge separation. The combination of these first two processes is often denoted as free charge generation. Finally, these free charge carriers need to reach the electrodes in order to be extracted to the outside, a process called charge collection/extraction. There is an ongoing debate about the minimum ΔIE required to guarantee efficient free charge generation but also about the main reasons for the decreasing performance of low-offset D:A blends.^9–12^ Nakano *et al.* demonstrated a strict correlation between the free charge generation efficiency and the LE–CT energy offset. It was concluded that the performance of low-offset devices is limited by inefficient charge generation rather than charge separation.^13^ Classen *et al.* arrived at the same conclusion, observing efficient charge generation for an ΔIE as small as 50 meV.^14^ Therein, an equilibrium model was postulated where a low energetic driving force for charge generation (the LE to CT transition) can be partially compensated for by a long lived LE state. In contrast, Karuthedath *et al.* and later Gorenflot *et al.* argued that a sizable bulk ΔIE of 0.5 eV is needed for efficient free charge generation. Only then is there a large enough driving force for LE dissociation to occur.^9,12^ These authors also highlighted the role of band-bending due to the large quadrupole moment of many NFAs, which lifts the CT state above the local LE for a very small ΔIE. Interestingly, these authors also noted a marked effect of ΔIE on the charge separation efficiency which is yet to be fully understood. In another work, Qian *et al.* probed a series of PM6 and PTO2 polymer-based OSCs, suggesting that low-offset systems display strong hybridization of the LE and CT states.^15^ They further argued that hybridization accelerates geminate recombination, thereby reducing the exciton-to-free charge conversion efficiency. In contrast, Jasiūnas *et al.* attributed the poorer free charge generation efficiency in low-offset blends to fast hole-back transfer.^16^ Finally, Müller *et al.* concluded that a reduced IE offset strongly hinders CT splitting as compared to LE dissociation, the former being attributed as the main factor responsible for the resulting low free charge generation.^17^

While different pictures were proposed about why low ΔIE limits device performance and over which range this deteriorates the PCE, a pronounced dependence of the photocurrent on external bias was consistently observed in low-offset OSCs. This implies that at least one of the fundamental steps in photon-to-collected-charge conversion is assisted by the internal electric field. Indeed, several groups reported a pronounced effect of the internal electric field on the photovoltaic quantum efficiency (EQE_PV_), the steady state photoluminescence (ssPL), the transient PL (trPL) lifetime, and the fate of ground-state bleach signatures in transient absorption (TA) spectroscopy. These observations have been attributed mainly to field-assisted charge separation^17–20^ and partially also to the field-assisted charge generation.^21–23^ However, the assignment of the observed field-dependence to one particular process may be difficult due to multi-step nature of photocurrent generation in OSCs For example, bias-dependent PL quenching has been studied in fullerene-based OSCs as well as in NFA-based systems as a means to study dissociation efficiency of LE and CT states.^13,15,17,20,24^ While it is true that a field-induced quenching of the ssPL intensity always indicates a depletion of the radiative LE states, other processes such as the repopulation of LE from non-dissociated CT states, possibly coupled to the reformation of CT states from free charges, are also influenced by the internal electric field. These additional processes may play a significant role in low-offset systems where the LE and CT populations are in kinetic equilibrium.^14,16,25^ Furthermore, bias-dependent EQE_PV_ can be caused by the electric field-dependence of not just charge generation, but also charge separation and eventually extraction. It is therefore crucial to disentangle these processes in a functional device.

In this work, we systematically explore the role of low driving force to free charge generation through a methodical assessment of generation and emission properties in a sample set of Y-series based OSCs. We begin with a detailed investigation of blends of the NFAs Y5 and Y6 with two separate molecular weights of the polymer donor PM6. By using PM6 with different molecular weights and two NFAs with different termination of the conjugated core (H- *versus* F-), we reduce the ΔIE in the blend from 350 meV to 210 meV, accompanied by a strong reduction of the device power conversion efficiencies (PCEs) from 15% to 1%. Poor PCEs are mainly caused by a pronounced field-dependence of free charge generation, probed *via* time-delayed collection field (TDCF) measurements. In such inefficient blends, TA spectroscopy also shows strongly diminished electro-absorption and polaron bands of the donor polaron, accompanied by negligible formation of excited states (ground state bleach) of the donor upon selective NFA excitation. In addition, bias-dependent TA spectra show accelerated formation of the donor ground state bleach and electro-absorption bands in the presence of an external field in devices, corroborating observations from TDCF. We further demonstrate that the enhancement in free charge generation under an effective electric field is accompanied by a concomitant reduction in ssPL of the blend, which is dominated by the emission of the NFA LE.^11,26^ Most importantly, we are able to accurately reconstruct the field-dependent photoluminescence quantum efficiency (PLQY) from the PLQY of the neat acceptor for all of the studied systems, taking into account the free charge generation efficiency from TDCF and the LE reformation efficiency, obtained from the combination of electro-(EL) and photoluminescence. Based on these findings, we conclude that photocurrent losses by geminate CT recombination are of very minor importance. Moreover, the emission properties of the NFA LE in the blend are found to be little affected by the presence of the donor polymer, *i.e.* by hybridization with the CT state. Supported by quantum chemical simulations we are, finally, able to reproduce the large effect of ΔIE has on the photogeneration efficiency using Marcus theory for charge transfer.

## Results

### (a) Optoelectronic properties

**Fig. 1:**
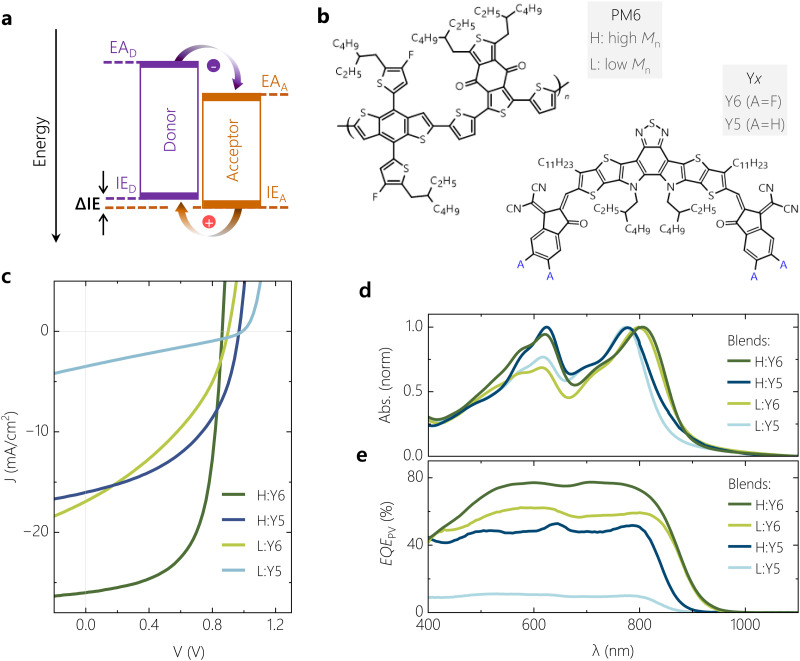
**Molecular structure, electronic, optical and photovoltaic properties of model systems.** (a) Schematic representation of a D:A heterojunction with relevant electronic levels and possible charge transition routes. (b) Molecular structures of PM6, Y5 and Y6. (c) Current voltage (*JV*) characteristics measured under simulated AM1.5G illumination, depicting the performance range of the four OSC model systems. (d) Normalised absorbance of blend films of comparable thickness and (e) photovoltaic quantum efficiency (EQE_PV_) of the four model systems measured under short-circuit conditions.

Fig. 1b shows the molecular structure of the polymer donor PM6 along with the two NFAs Y5 and Y6. Details on the synthesis and molecular weight of PM6 and full chemical names of the components are given in Supplementary Note 1 (ESI†). The two NFA molecules differ in their terminal sites, *i.e.*, Y6 is fluorinated while Y5 is not. As a consequence, a *ca.* 0.1 eV smaller IE has been reported for Y5.^27^ To tune the offset further, we combined these two NFAs with PM6 from commercial sources (with high number-average molar mass (*M*_n_) of >20 kDa), labelled as H or H_PM6_, and with synthesized PM6 of low *M*_n_ of *ca.* 3.5 kDa, denoted as L or L_PM6_, with the synthesis described in ref. 28 and the ESI.† It has been shown recently that PM6 of lower molecular weight aggregates less in solution and blend films, as documented by temperature-dependent absorption spectroscopy, atomic force microscopy (AFM) and transmission electron microscopy. It was further concluded that a lower degree of polymer aggregation increases the IE and reduces the ΔIE in the blend.^28^ These findings are in full agreement to earlier studies, where it was found that a higher molecular weight fosters chain aggregation of polydisperse conjugated polymers in solution and in solid state.^29–31^ This is explained by stronger interchain interactions, but also the decreasing role of chain ends acting as structural defects in the solid films. Also, a systematic reduction of the polymer IE with increasing degree of aggregation has been widely observed, which is due to of a stronger delocalization of holes on large chain aggregates.^30,32^ To confirm that the PM6 polymer weight affects the polymer aggregation properties in our samples also, AFM was performed on the neat polymer films and on our four blends (see Fig. S1 for the AFM heights profiles, ESI†). Neat films of L_PM6_ completely lack the fibrillar structure typically seen for films of neat H_PM6_, while we also notice an overall smoother surface topology of both L_PM6_-based blends.

The current–voltage (*JV*) characteristics of regular devices with comparable thicknesses of the photoactive layer (100–110 nm) are shown in Fig. 1c (see Fig. S2 (ESI†) for the statistics of the photovoltaic parameters). While the H:Y6 blend exhibits a typical PCE of up to 15%, the device performance drops considerably upon replacing Y6 by Y5. This is because the reduction in *J*_SC_ and FF overcompensates the increase in *V*_OC_. Our earlier studies suggested inefficient and field-dependent exciton dissociation as the primary reason for the poor performance of the PM6:Y5 blend, related to a smaller ΔIE.^11,26,33^ Replacing H_PM6_ by L_PM6_ changes the device parameters in a similar way, suggesting a reduced ΔIE as the main cause for the poor performance, herein arising from reduced polymer aggregation as described earlier. This varies the PCE by more than a factor of ten, from on average 15% for H:Y6 down to 1% for L:Y5, while keeping the chemical structure of the conjugated backbone of the constituents nearly the same. We note here that a strong decrease in performance was reported by Karki *et al.* when blending Y6 with a *ca.* 1 : 1 mixture of high and a low *M*_n_ PM6, which was attributed mainly to a different bulk and interface morphology between the cases.^29^ We will show later that the reduction in PCE is not primarily caused by differences in charge extraction. Evidence for the different aggregation properties of the two molecular weight batches, in neat layers and in the blends, comes from the absorption spectra in Fig. S3 and Fig. 1d (ESI†), respectively. While the shape and spectral position of NFA absorption in the blend is nearly unaffected by the *M*_n_ of PM6, replacing of H_PM6_ with L_PM6_ leads to an overall reduction in the polymer peak absorption strength, but also of the 0–0 : 0–1 peak ratio. Based on the results of absorption spectroscopy on neat PM6,^28,34^ these spectral changes are interpreted to originate from weaker polymer chain aggregation, as noted earlier. The corresponding EQE_PV_ spectra are shown in Fig. 1e. Replacing Y6 by Y5 and/or H_PM6_ by L_PM6_ reduces EQE_PV_ over the entire spectral range but has little effect on the shape of the spectra. EQE_PV_ spectra generally differ from the absorption spectra because photons which are reflected at the cathode also contribute to photocurrent generation. Obviously, the reduced photocurrent generation in our OSCs with Y5 or L_PM6_ does not originate from effects related to individual material properties such as very short exciton diffusion lengths in one of the blend components. We propose the free charge generation process in such low-offset OSC systems is dictated by the hole-transfer process even when the excitons are initially generated in the donor, in agreement to results from previous studies.^12^

### (b) Blend energetics

*In situ* spectroelectrochemistry (SEC), *i.e.*, coupling cyclic voltammetry with *in situ* UV-vis-NIR spectroscopy, is used to determine the IEs of the four blends with identical preparation as used for the solar cell characterization with ITO as the substrate. This technique has proven to be useful in terms of mapping the spectral evolution during charging in electrochemical experiments and determining the oxidation and reduction onsets from the spectral onsets of neutral and first charged species (polaron).^35^ In particular for blend films, there lies a problem of overlapping charged states which makes it impossible to extract the onsets from the cyclic voltammograms alone. In an earlier study we have shown the advantages of the spectral onset determination for the system PM6:Y6 with different molecular weights, spin-coated from other solvents.^35^ A general finding was that PM6 has a lower oxidation onset than Y6, *i.e.* higher IE. Here, we focus on the oxidation of the blend films which involves the potential scan to positive potentials. From the oxidation onsets, the IEs and ΔIEs can be calculated which will be later used for a comparison with the open circuit potentials *V*_OC_. The oxidation of neat films of the single compounds (identical preparation as for blends) was done in parallel and is used to help identify characteristic absorption bands in the blends.

**Fig. 2:**
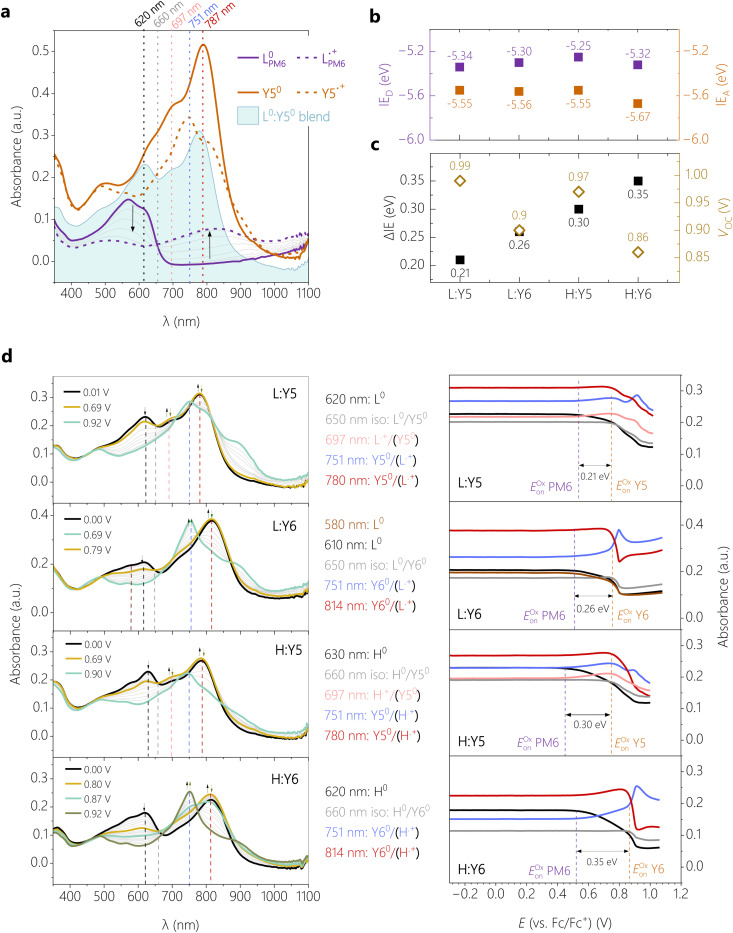
**Blend energetics.** (a) Absorption spectra recorded during cyclic voltammetry of the neat films of L_PM6_ and Y5, and the blend of L_PM6_ and Y5 in the neutral form (at 0.00 V *vs.* Fc/Fc^+^ for neat and blend) and the radical cation form of neat L_PM6_ (0.97 V *vs.* Fc/Fc^+^) and Y5 (0.92 V *vs.* Fc/Fc^+^). The vertical dashed lines are guides-to-the-eye to highlight characteristic bands of the different species. (b) Ionization energies (IE) of donor and acceptor measured in the blends. (c) Comparison of open circuit potential *V*_OC_ with the IE offsets ΔIE as determined from the data in (b). (d) Absorption spectra (left) for selected potentials recorded during oxidation, and the corresponding peak trends (right) for the different blends L_PM6_:Y5, L_PM6_:Y6, H_PM6_:Y5 and H_PM6_:Y6 (top to down).

In Fig. 2a, the characteristic spectra of films of neat L_PM6_, neat Y5 and the blend L_PM6_:Y5 are shown for the neutral state. The corresponding CVs are summarized in the Fig. S4 (ESI†). During a positive potential sweep, the neutral absorption bands of L_PM6_ at 570 nm and 620 nm decrease and a broad bathochromically shifted band is appearing with its peak at 820 nm, indicating the radical cation form 
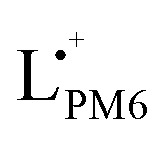
. One can also clearly identify a characteristic isosbestic point at 660 nm. For neat Y5, the spectral response during cyclic voltammetry is a decrease of the 787 nm band of the neutral molecule and the appearance of a new hypsochromically shifted band maximum at 751 nm, which is assigned to the formation of the radical cation form 
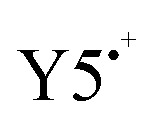
. The neutral blend spectrum L_PM6_:Y5 resembles the neutral spectra of the single components, with slight shifts which can be explained by the superposition of the spectra. The maximum around 620 nm in the blend spectrum can be attributed to L_PM6_, whereas the maximum at 780 nm to the Y5 species. Following the same principle, the main PM6 and the main Y*x* absorption bands can be assigned in all blends (ESI,† Fig. S5). For the Y6 systems, the maximum of Y6 is bathochromically shifted to 814 nm with respect to Y5.

In Fig. 2d (left), the spectra of the four blends during oxidation are shown in the potential range between 0.00 and ∼1.00 V *vs.* Fc/Fc^+^, the intensity evolution of characteristic absorption bands is highlighted in Fig. 2d (right). The corresponding oxidation cycles can be found in Fig. S6 (ESI†). For L_PM6_:Y5, increasing the potential from 0.00 V (black spectrum) to 0.69 V (dark yellow spectrum) goes along with a decrease of the intensity of the absorption maximum at 620 nm which is characteristic for the continuous oxidation of the neutral L^0^_PM6_. Parallel to this, the increase of absorption at 697, 751 and 780 nm indicates the formation of the radical cation 
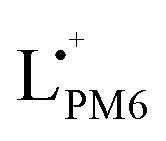
. Consistent with the spectra evolution of neat L_PM6_ an isosbestic point at 650 nm can be observed in this potential range. The peak evolutions in Fig. 2d (right) can be used to determine the oxidation onsets *via* the tangent method.^35^ This method involves the transfer of the potential onsets to the Fermi scale with a correction factor of −4.8 eV. Hereby, the bands at 620 nm and 697 nm are used to determine the oxidation onset *E*^ox^_on_ (L_PM6_) of PM6, which gives 0.54 V equivalent to IE_D_ = −5.34 eV. Increasing the potential from 0.69 V to ∼1.00 V, the band at 780 nm is decreasing and the band at 751 nm is appearing, showing the oxidation of neutral Y5^0^ to the radical cation 
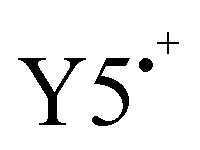
. Unfortunately, the oxidation of Y5 goes along with an overall decrease of the absorption between 600 and 900 nm, which aggravates the determination of the exact oxidation onset from these spectra. We, therefore, base the determination of the oxidation onset *E*^ox^_on_ (Y5) of Y5 on the absorption at the isosbestic point of PM6 in the blend at 650 nm, which yields a value of 0.75 V or an IE_A_ of −5.55 eV.

The evaluation of the blend H_PM6_:Y5 follows qualitatively the same procedure as for the L_PM6_:Y5. Hereby, the oxidation onset of *E*^ox^_on_ (H_PM6_) is determined at 0.45 V, equivalent an IE_D_ of −5.25 eV. The oxidation onset of *E*^ox^_on_ (Y5) is again determined from the absorption at the isosbestic point of PM6, yielding 0.75 V or an IE_A_ of −5.55 eV. The IEs of the Y6-based blends have been analyzed the same way. Here, we additionally benefit from the more pronounced increase of the absorption at 751 nm, assigned to the radical cation of Y6. Note that different L_PM6_:Y6 samples revealed slightly different spectral shapes but also different oxidation onsets, while the value of ΔIE was reproducible at 0.30 ± 0.02 V. Probably this can be explained by the low molecular weight of this polymer and the lower aggregation tendency, which might lead to dissolution of the blends during the electrochemical experiment. Therefore, the absolute values of IE_A_ and IE_D_ of L_PM6_:Y6 should be interpreted with caution.

Fig. 2b plots the ionization energies of the four blends. With the exemption of the L_PM6_:Y6, the trends follow the expectations from the blend absorption as discussed earlier. For the Y5-based blends, the *ca.* 0.1 eV reduced IE of H_PM6_ compared to L_PM6_ is consistent with a stronger aggregation of the H_PM6_ in the blend with Y5. In contrast, replacing Y5 by Y6 in the blend with H_PM6_ increases the IE_A_, which is consistent with the reported down-shift of the IE upon fluorination.^27^ On the other hand, we find similar values of the Y5 NFA in blends with H_PM6_, which agrees with the similarities in Y5 absorption of the blend. We note again that the L_PM6_:Y6 shows overall lower ionization energies, which is a direct outcome of the SEC as described earlier.

The resulting ΔIE is compared to the device *V*_OC_ in Fig. 2c. The highest performing H_PM6_:Y6 blend, with a PCE of over 15% and a *V*_OC_ of 0.86 V, has the largest IE offset of 0.35 eV while the poorest performing blend, L_PM6_:Y5 with an PCE of 1% and a *V*_OC_ of 0.99 V, has the smallest IE offset of 0.21 eV. Here we remind the reader that the correct determination of ΔIE in the PM6:Y6 blend is among the hottest debated topics of research on the PM6:Y6 blend.^36^ In the past, these energies were mostly derived from CV and UPS on neat layers, with large differences in the final results. For example, the CV data in the first PM6:Y6 paper^37^ yielded an ΔIE of less than 0.1 eV while UPS data from different sources yielded ΔIE > 0.5 eV.^27^ However, a ΔIE of < 0.1 eV is too small to split the Y6 excitons, which have a binding energy of *ca.* 0.3 V.^35^ On the other hand, with the well-established fundamental gap of 1.7 eV for Y6, ΔIE of *ca.* 0.5 eV translates into an energy of the charge separated states (*E*_CS_) of less than 1.2 V, which is rather small in light of a *V*_OC_ of 0.83 V. A ΔIE of *ca.* 0.3–0.34 eV as reported here and in our previous work for H:Y6^35^ should be sufficiently large to dissociate the Y6 exciton while providing a high enough *E*_CS_ of 1.4 eV to explain the blend *V*_OC_. Regarding our other blends, the increase in *V*_OC_ relative to H:Y6 is qualitatively in agreement with the trend in ΔIE when taking into account that Y5 has a *ca.* 30 meV larger IE-EA gap than Y6.^27^ As outlined earlier, ΔIE provides an only rough estimate of the energy of the CT state, the latter being relevant for the value of *V*_OC_ in most organic solar cells.^38^

### (c) Anticorrelation of field-dependent free charge generation and emission

**Fig. 3:**
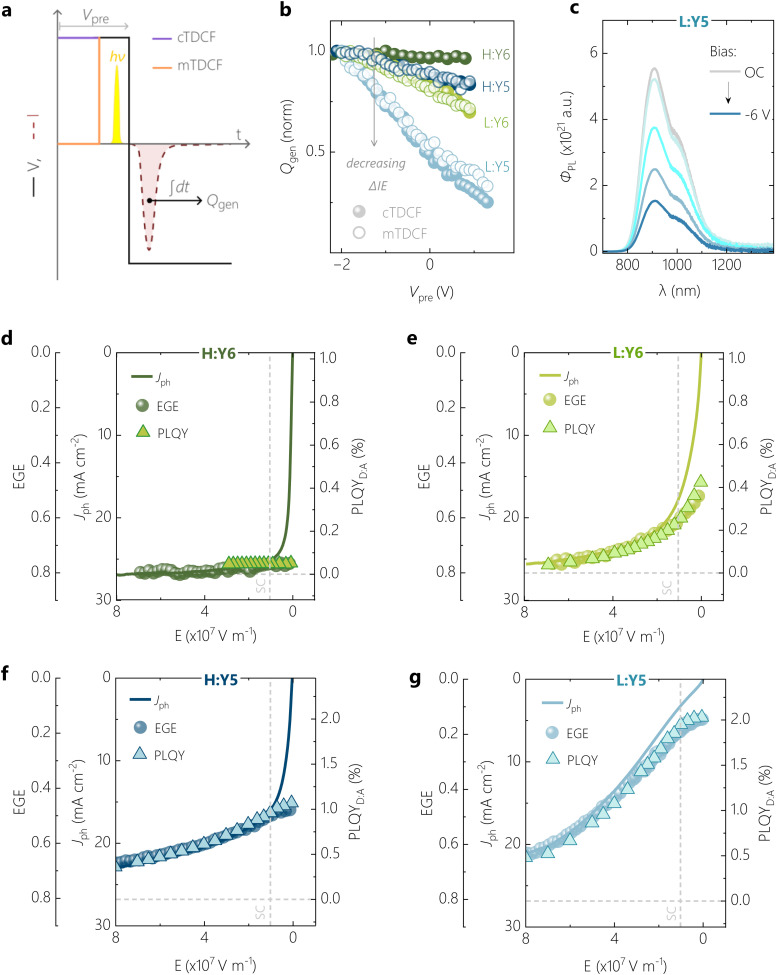
**Ubiquitous anticorrelation of bias-dependent LE emission and free charge generation.** (a) Schematic illustration of classical and modified time-delayed collection field (cTDCF and mTDCF) techniques during one extraction cycle. (b) Normalised comparison of the photogenerated charge *Q*_gen_ as a function of applied pre-bias *V*_pre_, measured using cTDCF (filled spheres) and mTDCF (hollow circles). (c) An example of steady state photoluminescence (ssPL) spectra, quenched with applied reverse bias, shown for the L:Y5 system. (d) Overlay of field-dependent photocurrent density (*J*_ph_), external free charge generation efficiency (EGE) from TDCF, and PLQY_D:A_ for the H:Y6 OSC, showing very good anticorrelation of generation and emission. The same is plotted for L:Y6, H:Y5 and L:Y5 in (e), (f), and (g), respectively. Horizontal dashed lines denote zero emission from the NFA in the blend, and vertical dashed lines denote the electric field at short-circuit (SC) condition.

In our previous studies on a blend of high *M*_n_ PM6 with Y5, we showed that an anticorrelation exists between free charge generation and the intensity of ssPL.^26^ This provided us with evidence that the bias-dependence of *J*_ph_ in this blend is largely determined by the field-dependence of the dissociation of the Y5 LE exciton. In the following, we will build on this by demonstrating quantitative relations between the bias-dependence of *J*_ph_, the external free charge generation efficiency (EGE) and of the photoluminescence quantum efficiency of the blend (PLQY_D:A_). As in ref. 26, our method of choice to measure the efficiency of exciton to free charge conversion is TDCF, see ref. 32 and 39 for details. A low laser fluence of *ca.* 60 nJ cm^−2^ (*λ* = 532 nm, pulse width = *ca.* 6 ns) was used to excite the device held at a pre-bias (*V*_pre_), followed by immediate collection of photogenerated free charge carriers using a high reverse collection bias (see Fig. 3a). In our current setup, a fast slew rate of 2 ns of the voltage source enables fast extraction of the photogenerated free charge (*Q*_gen_). While these experimental conditions minimize losses due to non-geminate recombination (NGR) among photogenerated charge carriers, recombination between photogenerated and dark injected charge carriers might be an issue, especially at positive *V*_pre_.^40^ To check whether this loss process is an issue in our samples, we cross-checked the results from classical TDCF (cTDCF) with our recently developed modified-TDCF, or mTDCF, technique.^26^ In contrast to cTDCF, the pre-bias is applied for only 25 ns in mTDCF, which is three times the RC time of our sample. With this, the injection of dark charge carriers into the bulk should be largely minimized while the device capacitance is charged with the applied voltage. Fig. 3b shows the *Q*_gen_ from cTDCF and mTDCF for the present four systems as a function of the pre-bias voltage, normalized at *V*_pre_ = −2 V. Both techniques demonstrate the same field-dependence of free charge generation, though our measurements were performed up to *V*_pre_ = *V*_OC_. It is only for the highly inefficient L:Y5 device that we measure up to 10% less free charge from cTDCF compared to mTDCF around and above *V*_OC_ conditions. While this indicates extraction losses due to recombination with dark charge in the positive pre-bias range, the effect is too small to account for the pronounced field-dependence of free charge generation derived from TDCF for this system. This is in agreement with our recent drift-diffusion simulations of TDCF experiments where we showed that even in the unlikely case of Langevin-type recombination, extraction losses in m-TDCF should be less than 10% at *V*_OC_.^41^ The results in Fig. 3b therefore highlight a clear correlation of progressive bias-dependent free charge generation with a diminishing ΔIE in the D:A blends.

Knowing *Q*_gen_ and the excitation fluence of the incident photons, we calculate the external free charge generation efficiency, or EGE, and correlate this with *J*_ph_. The applied bias is expressed as an effective electric field *E* = (*V*_bi_ − *V*)/*d*, where *V* is the applied bias and *V*_bi_ is the built-in voltage of the respective OSC determined at zero-photocurrent at steady state. As shown in Fig. 3d–g, EGE(*E*) overlaps almost perfectly with *J*_ph_(*E*) over a broad electric field range for all four systems when scaling the two properties such that an EGE of 80% corresponds to a *J*_ph_ of 27 mA cm^−2^, *i.e.* of the high performing H:Y6 blend (see Fig. S7 for a condensed representation of the data, ESI†). It has been shown that photon losses due to reflection and parasitic absorption account typically for a *ca.* 20% loss of maximum photogenerated free charge.^42^ Deviations between EGE(*E*) and *J*_ph_(*E*) are present mainly at low electric-fields, arising from NGR which additionally reduces *J*_ph_. These non-geminate losses become more pronounced with decreasing ΔIE. It was shown in recent studies that low-offset D:A blends exhibit a larger NGR coefficient, though the reason for this correlation is still unknown.^11^ We conclude that *J*_ph_(*E*) is governed by the field-dependence of free charge generation over a wide electric field range, with only small contributions of NGR. Notably, even the low performing L:Y5 blend achieves an EGE larger than 60% at an electric field of 8 × 10^7^ V m^−1^ (corresponding to −7 V reverse bias), more than three times the value at the short-circuit condition.

At this point, a question arises whether the field-dependence of EGE of these systems, measured by predominantly exciting the donor at *λ* = 532 nm, is fully representative of the generation properties of each D:A blend over a wide spectral range. Consider the scenario where ΔEA is larger than ΔIE, as is the case in our systems. If photogenerated excitons in the donor would, for instance, dissociate at a D:A interface prior to resonant energy transfer to the NFA, then electron transfer from donor excitons is expected to be more efficient and hence less bias-dependent than hole transfer from NFA excitons. To address this, we recorded EQE_PV_ for different biases on the two D:A systems with strongest bias-dependent EGE, *i.e.* L:Y6 and L:Y5. As shown in Fig. S8a (ESI†), an increasing negative bias increases the EQE_PV_ over the entire spectral range for both systems. Inspection of the normalised EQE_PV_(*V*) of L:Y6 in Fig. S8b (ESI†) shows the shape of the spectra to be completely unaffected by the bias, while the normalised EQE_PV_(*V*) of L:Y5 exhibits a marginally enhanced bias-effect when exciting the Y5 acceptor than when exciting the L_PM6_ donor. TDCF experiments with selective excitation of the Y5 NFA at *λ* = 800 nm prove that the free charge generation is indeed marginally more bias-dependent, but that this effect is fairly small compared to selective donor excitation at *λ* = 532 nm (a difference of *ca.* 5% at *V*_pre_ = *V*_OC_, see Fig. S9c and d, ESI†). This provides further evidence that the hole transfer pathway is indeed the critical step in the photogeneration process for equally all blend systems, which we later corroborate with transient absorption data in sub-ps timescales.

We next measured the bias-dependence of ssPL spectra at a fixed *λ* = 520 nm excitation and at an intensity so as to produce the same short-circuit current as under AM1.5G illumination. These measurements were taken on the very same device structures as measured in TDCF, and it was ensured that the excitation spot is focussed only onto the device area. For all blends, the spectral position and shape of the PL differs only marginally from that of the neat acceptor dispersed in a polystyrene matrix (PS:NFA) with same concentration as in the D:A blends (denoted as PS:Y*x* in the following), see Fig. S10 (ESI†). Such spectral differences are likely due to microcavity effects in the full device, as well as minor differences in the NFA aggregation properties.^43,44^ The overlapping ssPL from D:A and PS:NFA shows that the PL in blends with the donor is governed entirely by the radiative decay of the respective NFA LE state with very little contribution from radiative CT decay. For the blends that exhibit a bias-dependent *J*_ph_, we observe a strong reduction of the PL photon flux (*Φ*_PL_) with increasingly negative reverse bias, as can be seen for L:Y5 in Fig. 3c and for L:Y6 in Fig. S9b (ESI†) (H:Y5 has been previously reported^26^), while the spectral shape is not affected by the applied bias (Fig. S10a and c, ESI†). If radiative recombination of the CT state were to contribute to the ssPL spectrum, its contribution would decrease rapidly with higher negative bias because charge extraction hinders the reformation of CT states *via* NGR. In such a case, spectral changes would be expected.^32^ In addition, we measured the EL spectrum at a voltage that produces the same recombination current as in the ssPL experiment. We find that the shape of the EL spectrum is very similar to the device ssPL, while its intensity is significantly lower (see Fig. S10b and d, ESI†). This confirms our view that all emission from these devices occurs *via* the local LE excitons.

In Fig. 3d–g, we correlate *Φ*_PL_ to *J*_ph_ and EGE as a function of the effective electric field. To do so, the bias-dependent PL peak intensities (*Φ*_PL,max_) were first converted into a PL quantum yield (PLQY_D:A_(*E*)), extrapolated from the measured PLQY of the device at *V*_OC_ using1
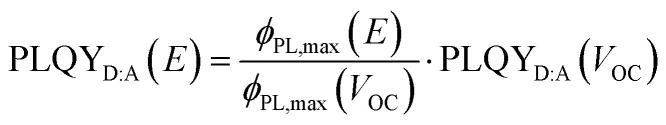
Here, PLQY_D:A_,(*V*_OC_) was measured on a full device structure in an integrating sphere under 1 sun equivalent illumination at open-circuit conditions. It was ensured that the excitation laser beam in the integrating sphere only illuminated the area comprising of the complete OSC device stack.

To correlate PLQY_D:A_(*E*) with EGE(*E*) and *J*_ph_(*E*), we first aligned PLQY_D:A_ = 0 to EGE = 80%; the condition where we concluded that all photogenerated excitons are converted into free carriers. We were then able to establish a nearly perfect anticorrelation between the field-dependences of PLQY_D:A_ and EGE for all four blends when we aligned EGE = 0% to the PLQY of the corresponding inert PS:Y*x* blend, *i.e.* PLQY_PS:A_ – where all recombination proceeds through the decay of the Y*x* excitons. This quantitative agreement leads us to the conclusion that the field-enhanced free charge generation comes directly at the cost of quenched excitonic emission. In other words, geminate exciton recombination is the main (if not only) decay channel competing with free charge generation. Note that the PLQY of the PS:Y*x* devices are field-independent (see Fig. S11, ESI†), which means that the internal electric field enhances charge generation only at the D:A heterojunction but not within the NFA domains.

### (d) Charge generation dynamics in transient absorption spectroscopy

We confirm these conclusions with measurements of the early time dynamics of the photoexcited species using TA spectroscopy. Given the fact that the ssPL is dominated by the NFA emission, we firstly performed TA measurements by selectively exciting the NFA at *λ* = 720 nm, with a low pump fluence of ≈2 μJ cm^−2^. TA spectra were recorded while probing in the visible (500–680 nm) and near infrared (NIR) regions (680–1350 nm) of the electromagnetic spectrum, resolved in time delays between 0.1 ps and 7 ns. As a benchmark, the optical transitions in pure NFAs, probed in films of PS:NFA, are overlaid on the TA spectra of different D:A blends.

**Fig. 4:**
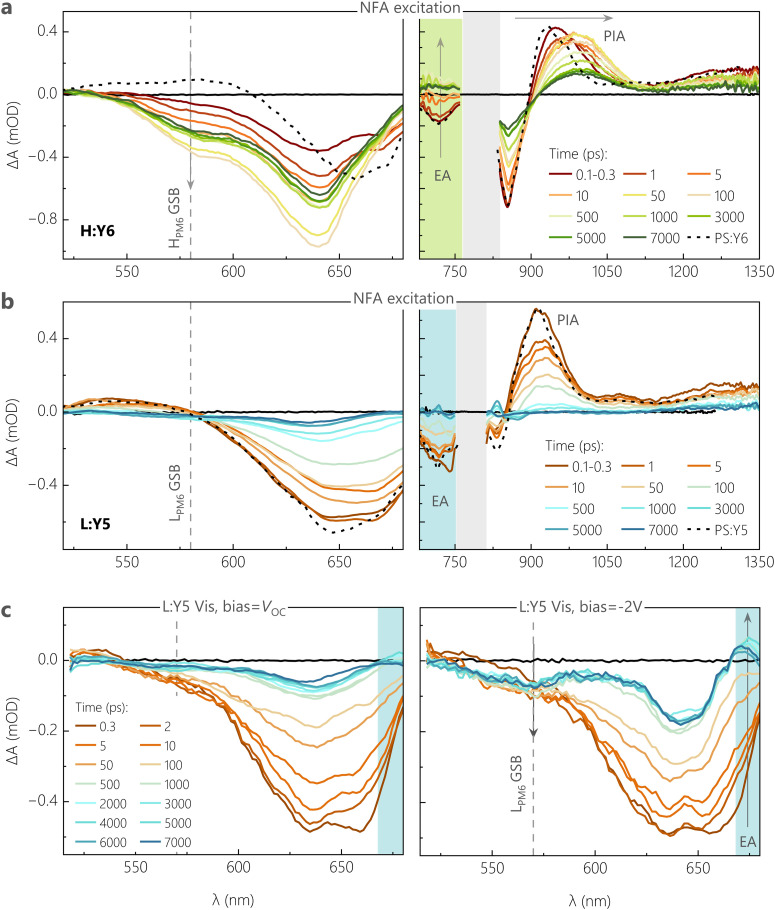
**Spectroscopic investigation (open-circuit and biased) of dynamics of free charge generation reflects field-dependent performance of model NFA blends.** Transient absorption spectra in the visible and infrared region, probed on films of the (a) H:Y6 blend and (b) L:Y5 blend, excited with a 1.77 eV laser pulse of 2 μJ cm^−2^ fluence for exclusive NFA excitation. The grey shaded area denotes the region of optical excitation of the probe beam. The black dotted lines denote the optical transitions of the respective PS:NFA films at 1 ps in order to benchmark the transient spectra of the blends. (c) Bias-dependent transient absorption spectra in the visible region probed on an L:Y5 device, when biased externally with *V*_OC_ and −2 V. The semi-transparent sample was excited with a 1.77 eV laser pulse of 6 μJ cm^−2^ fluence for selective NFA excitation.

Fig. 4a shows the visible and NIR TA spectra for H:Y6 at different time delays between the incident pump and probe pulses. At very short time delays (0.1–0.3 ps), we observe a negative feature due to the ground state bleach (GSB) of Y6 at *λ* > 600 nm, along with a substantial GSB contribution from H_PM6_ between 580–600 nm in the visible region.^45,46^ The presence of H_PM6_ GSB upon selective pumping of Y6 provides confirmation of hole transfer at ultrafast time scales (<0.3 ps). A similar observation is made from the NIR region, wherein the formation of new positive bands between 700–750 nm is attributed to the electro-absorption (EA) feature of H_PM6_, arising from the Stark-shift of the H_PM6_ absorption due to the electric field caused by charge separated species.^45,47^ The EA feature is accompanied by spectral red shifts and broadening of the positive photoinduced absorption band (PIA), as referenced against the PIA feature of pure Y6 (peak at 925 nm). This is attributed to the growth of a polaron absorption band centred at 980 nm due to contributions from hole absorption of PM6^48^ with possible contributions from anion absorption band of Y6.^36,49,50^ With progression of the delay, both the H_PM6_ GSB and the EA features rise and complete their evolutions at ≈100 ps (see Fig. S12c, ESI†). It is important to note that even with the initial sub-picosecond rise, the complete hole transfer process takes about 100 ps to reach its peak due to the time required for excitons formed within Y6 domains to diffuse to the interface, as has been previously reported for Y6 and several other NFAs.^51^ In this context, we acknowledge recent TA work suggesting that free charge formation in Y6-based OSCs occurs mainly *via* the dissociation of intermolecular (intramoiety) charge transfer states (often denoted as *x*CT), especially at longer delay times.^46,47^ On the other hand, analysis of the EQE_PV_ spectra of CuSCN/NFA bilayer devices with different NFA layer thickness^52^ and of the intensity dependence of pulsed PL measurements on neat NFA layers^53^ revealed similar values for the exciton diffusion lengths. This suggests that the *x*CT and the LE are in kinetic equilibrium (at least at room temperature) through, for example, thermal re-excitation of the LE state^47^ and/or through strong electronic coupling.^54^ The TA measurement for the L:Y6 blend with smaller ΔIE was performed under the same conditions for a clear comparison, see Fig. S12a (ESI†) for the visible and NIR region TA spectra. Unsurprisingly, the spectral signatures of H_PM6_ GSB, EA and the polaron absorption closely match those of H:Y6. However, compared to H:Y6, L:Y6 exhibited a muted rise in both H_PM6_ GSB and EA features. The comparatively slow rise component of these features in L:Y6 can be observed in both the initial sub-picosecond regime as well as the generation timescales attributed to the exciton diffusion-dependent component (see Fig. S12c, ESI†). Fig. S12b (ESI†) further shows the comparison of the negative GSB dynamics for H:Y6 and L:Y6 blends, wherein the H:Y6 (L:Y6) reaches its peak at ≈100 ps (≈200–300 ps). As a consequence, exciton recombination competes efficiently with charge generation in L:Y6, in full support of our findings from TDCF and ssPL. Fig. S12c (ESI†) shows the comparison of PM6 GSB and the EA feature for H:Y6 and L:Y6 blends. Clearly, the EA feature is found to closely mirror the rise in PM6 GSB, suggesting instantaneous dissociation of and almost no accumulation in the population for CT states.

Next, we move our focus to the Y5 acceptor blends. Fig. S32a (ESI†) shows the visible and NIR region TA spectra for H:Y5 at a pump fluence of 2 μJ cm^−2^. The GSB of Y5, peaking at 640 nm, shows a minor rise in intensity at early time delays (<5 ps) due to hole transfer, resulting in an overlapping contribution from H_PM6_ at this wavelength and this is followed by a strong decay of Y5 excitons as the time progresses. At 50 ps, a GSB feature arises around 580 nm – which is selectively attributed to H_PM6_ GSB – following the decay of the overlapping positive feature of PIA from the Y5 exciton. Similarly, in the NIR region, the positive exciton PIA band at 900 nm Y5 shows a minor red shift and broadening due to the overlapping hole absorption of PM6 in the early time scales, and this is followed by a consistent decay due to inefficient dissociation of Y5 excitons. This is in clear contrast to the H:Y6 blend, wherein the strong contribution from H_PM6_ GSB is visible from the very early time delays due to a more efficient hole transfer. In most contrast to H:Y6, Fig. 4b shows the TA results for L:Y5, the most inefficient blend with the smallest ΔIE, under the same pump fluence. Both visible and NIR TA spectra are dominated by excitonic decay of GSB and PIA features as referenced against the pure Y5. The negligible hole transfer in the blend is clear from the feeble generation of L_PM6_ GSB at the 580–590 nm wavelength window. To get a meaningful comparison of hole transfer and free charge generation efficiency between H:Y5 and L:Y5 blends, we plot the GSB dynamics at 630–640 nm, corresponding to overlapping GSB of PM6 and Y5, see Fig. S13b (ESI†). The comparison of dynamics at early time scales shows an obvious rise in GSB intensity in H:Y5 which is absent in L:Y5. Fig. S13c (ESI†) shows the longer time dynamics normalised to the initial intensity of the two Y5-based blends along with the normalised GSB decay of PS:Y5. H:Y5 clearly shows a higher intensity at longer time due to the formation of longer-lived free charge carriers, while the dynamics of L:Y5 are closely aligned with that of PS:Y5. These results are indicative of the fact that in L:Y5 system, the TAS dynamics are dictated by the intrinsic photophysical properties of Y5, with minimal contribution from L_PM6_ GSB. A similar observation has been made for several other poorly performing NFA-based blends with diminishing energy offset.^12,55,56^ These results closely follow the trend shown earlier in TDCF generation at *V*_OC_ and signify the inefficiency in charge generation in the low-offset systems.

To confirm and corroborate the observation of field-dependent exciton dissociation in earlier sections, we performed bias-dependent TA measurements on H:Y6 and L:Y5 blend systems due to their very different field-dependent free charge generation characteristics shown in TDCF measurements. Measurements were performed on semi-transparent devices with the same device structure as that used for photovoltaic and TDCF studies, but with a thinner 50 nm active layer and with the thickness of silver electrode reduced from 100 to 15 nm. The biased TA measurements were performed in the visible region of the probe to avoid artefacts from longer probe wavelengths due to the semi-transparent contact.^57^ To achieve sufficient signal to noise ratio, a pump fluence of 6 μJ cm^−2^ was used for both devices. The biased-TA response for H:Y6 device under *V*_OC_ conditions and at −2 V, shown in Fig. S14a (ESI†), was found to be comparable and also similar to those recorded on bare films. The GSB feature around 580 nm (selective to PM6) shows a fast sub-picosecond rise along with a relatively slow rise, reaching its peak after *ca.*70–80 ps. Fig. S14b and c (ESI†) shows the TA dynamics of the different GSB bands of H:Y6 devices at 580 and 640 nm, respectively, under different bias conditions. Clearly, the presence of an external field does not alter the free charge generation characteristics of this system. We note that the dynamics at 580 nm in biased TA of devices are slightly different for those seen in bare films and can be attributed to the relatively higher fluences used. The field-dependence of generation is, however, clearly observable in the TA spectra for L:Y5 devices at *V*_OC_ conditions and −2 V, shown in Fig. 4c. At *V*_OC_, the TA spectra show a continuous decay of the Y5 GSB due to the decay of Y5 excited states and almost no contribution from the L_PM6_ GSB at lower wavelengths (570–580 nm), suggesting feeble hole transfer in this blend system at *V*_OC_ conditions. This is consistent with the TA results on bare L:Y5 films in Fig. 4b. Under a bias of −2 V, the GSB decay of Y5 is contrastingly subdued with relatively higher intensity of the GSB at longer time delays (>500 ps), suggesting a higher contribution from long-lived free charge carriers. Further, we notice a clear rise in GSB contribution at lower wavelengths (520–570 nm band) in early time scales up to 5 ps, which can be safely attributed to the L_PM6_ GSB as a result of the hole transfer process. This is also accompanied by a negative absorption band at 670 nm which is attributed to the EA feature arising from the generation of free charge carriers. The clear rise in L_PM6_ GSB with an external bias, along with the concomitant rise in EA, confirms that the poor performance of this blend system indeed originates from the inefficient dissociation of LE states to CT, but also that charge generation can be promoted by an electric field.

**Fig. 5:**
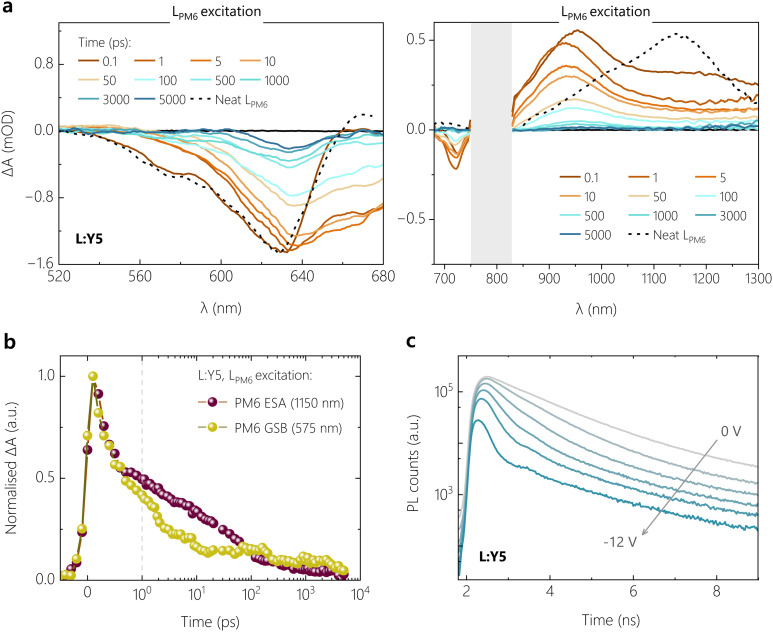
**Preferential donor excitation TAS, and transient photoluminescence trPL.** Transient absorption spectra of L:Y5 at pump excitation wavelength of 400 nm in the visible (a) and infrared region (b) at a pump excitation wavelength of 400 nm. The optical transitions of L_PM6_ at 1 ps are overlaid as a dotted line. The grey shaded area in the infrared region denotes the region of optical excitation of the probe beam. (c) Comparison of kinetic traces of the exciton PIA and GSB features of L_PM6_, demonstrating efficient Förster resonance energy transfer as the dominant process. (d) trPL decays for the L:Y5 device as a function of applied reverse bias.

To complete the TA study, we addressed the role of electron transfer. To this end, we performed TA experiments on the poorest performing blend system, L:Y5, with preferential excitation of the donor at 400 nm. At this excitation wavelength, L_PM6_ is found to contribute *ca.* 83% of the total blend absorption as benchmarked by comparing the L:Y5 blend with PS:Y5 films. Fig. 5a shows the resultant visible and NIR TA results for the L:Y5 blend, using a pump fluence of 3 μJ cm^−2^ (to yield a similar exciton density as in selective NFA excitation measurements). At 0.1 ps, the GSB of L:Y5 is found to be dominated by L_PM6_ with a shoulder at 575 nm, as shown by the overlapping GSB of the neat L_PM6_ in Fig. 5a. As time progresses, this feature is rapidly quenched along with the simultaneous rise of a GSB feature between 650–680 nm attributed to the GSB of Y5. This is further accompanied by the rapid reduction in the feature at around 1150 nm assigned to the PIA of L_PM6_ LE, as seen in Fig. 5a. Fig. 5b shows the comparison between the kinetic traces of L_PM6_ PIA at 1150 and the L_PM6_ GSB feature at 575 nm, wherein both demonstrate almost complete overlap in the early sub-picosecond timescales. The simultaneous reduction in the exciton PIA and the GSB of L_PM6_ in the early timescale suggests that PM6 LE states rapidly undergo Förster resonance energy transfer to yield Y5 LE states, and hence the overall charge generation step is limited by poor hole transfer.

An important consideration when comparing TA spectra results with ssPL, *JV* and TDCF is the relatively higher excitation density in TA measurements. It has been shown that singlet–singlet annihilation (SSA) already sets in at an exciton density of 1 × 10^16^ cm^−3^ in Y-series NFAs, which is a factor of *ca.* 13 lower than that when exciting our samples at a fluence of 2 μJ cm^−2^.^53^ This is also why the free charge generation efficiency in TAS is lower than that in TDCF; SSA competes with exciton dissociation in TAS but not in TDCF. We, therefore, complemented our studies with bias-dependent transient PL (trPL) measurements on both Y5 blends, with the results shown in Fig. 5c and Fig. S15a (ESI†) for L:Y5 and H:Y5 devices, respectively. For both blends, the trPL transients display two distinct time regimes: an early regime up to *ca.* 5 ns, in which the increasing reverse bias progressively speeds up the trPL signal decay and a regime at longer timescales where the magnitude of signal decay shows a strong bias-dependence while the decay rate is weakly affected. Both blends exhibit similar decay properties, but the bias-dependence on the trPL shape and magnitude is much more pronounced for the L:Y5 blend. By comparing the trPL kinetics of the L:Y5 blend with that of PS:Y5, we see that the second trPL decay regime of the blend corresponds exactly to the longer time decay of neat Y5 excitons, which have an average lifetime of 2 ns (see Fig. S15b, ESI†). This rules out that exciton reformation from long-lived CT states contributes significantly to the trPL signal in the considered ns time range.

### (e) Analytical model of blend emission

From the above experimental observations, we arrive at the following conclusions:

(i) There is no evidence for the build-up of an appreciable CT state population in the process of free charge generation, wherein geminate CT recombination would compete with charge separation. We conclude this from (a) the quantitative anticorrelation between the NFA LE ssPL in the blend and the free charge generation efficiency from TDCF, (b) the fact that the growth of the PM6 GSB goes nearly in parallel with the appearance of an EA signal in TAS (see Fig. S12c, ESI†), and (c) the distinct similarity in longer time trPL decay characteristics of the NFA when blended with a donor or dispersed by PS matrix.

(ii) There is no evidence for LE–CT hybridization, which would provide the LE state with a stronger CT character affecting its emission properties.^58–60^ This conclusion is reached based on (a) the excellent agreement of the PLQY of the blend, extrapolated to zero free charge generation, with the PLQY of the respective NFA in an inert PS matrix (see Fig. S16, ESI†), but also on (b) the lack of early charge generation in the poorly-performing system in TA measurements.

For all systems that obey the above two conditions, it must be possible to predict the absolute PLQY of the blend (PLQY_D:A,pred_) from only the field-dependence of the free charge generation efficiency and the probability that free charge recombination reforms LE excitons, according to:

2 PLQY_D:A,pred_(*E*) = PLQY_PS:A_(1 − IGE(*E*)) + ELQY_D:A_*J*_NGR,norm_(*E*)

**Fig. 6:**
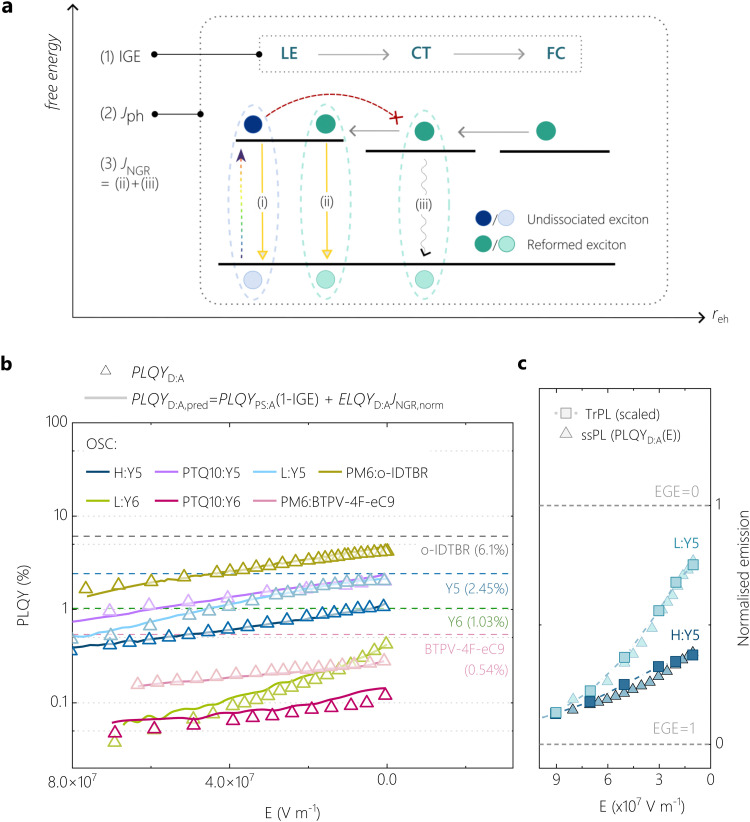
**Accurate reconstruction of experimental PLQY using a singlet-decay-limited generation model.** (a) An illustration of the physical origins of *J*_ph_, EGE and non-geminate bimolecular recombination current *J*_NGR_. The EGE is the product of forward processes charge generation and charge separation into free charge (FC) states. Various recombination pathways that can occur also are illustrated in the scheme. The blue circles represent undissociated LE excitons on the NFA that decay in competition with charge generation (emission pathway (i)). Green circles represent exciton states formed by free charge carriers that undergo NGR: either *via* CT states (emission pathway (iii)) or *via* further LE state reformation (emission pathway (ii)). (b) For various OSCs, a comparison of the field-dependence of the predicted PLQY_D:A,pred_ (solid lines, reconstructed using eqn (2)), and the experimental PLQY_D:A_(*E*) (symbols, obtained from field-dependent ssPL and eqn (1)). (c) Agreement between the steady-state normalised PLQY_D:A_(*E*) and the field-dependence of trPL quenching, which is reconstructed from field-dependent exciton splitting efficiencies for L:Y5 and H:Y5 (see Supplementary Note 2, ESI†).

The elements of this model are illustrated in a simplified three-state diagram in Fig. 6a. The first term denotes photon emission from local excitons on the NFA, which decay back to the ground state in competition with their separation into free charge. This is illustrated by emission pathway (i) in Fig. 6a. From optical simulations and our comparison of the EGE with the saturation photocurrent of H:Y6, we conclude that 80% of the incident photons are absorbed in our PM6:Y6 blends. We can, therefore, approximate the probability that an absorbed photon generates a free charge carrier by the field-dependent internal generation efficiency IGE(*E*) = EGE(*E*)/0.8. Since our experiments did not reveal any evidence for hybridization of local excitons and CT states, we then set the radiative efficiency of undissociated NFA excitons in the D:A blends equal to PLQY_PS:A_, *i.e.* the PL quantum efficiency in the absence of the donor, which was earlier found to be field-independent. Accordingly, the fraction of initially absorbed photons reemitted from undissociated LE states against the competition with free charge generation is given as PLQY_PS:A_·(1 − IGE(*E*)). The second term describes NFA photon emission in the blends by the reformation of LE states from free charge recombination.^11,15^ The probability that an absorbed photon results in re-emission from a reformed NFA LE state is given by ELQY_D:A_·*J*_NGR,norm_(*E*). Here, ELQY_D:A_ is the EL quantum efficiency of the blend and *J*_NGR,norm_ is the non-geminate recombination current density normalized to the saturation current density *J*_gen_. In our PM6:Y*x* blends, we set *J*_gen_ equal to 27 mA cm^−2^ as outlined earlier, which represents the case that every photogenerated exciton creates a free charge carrier.

Fig. S15 in ESI† graphically shows the experimental values of IGE and *J*_NGR,norm_ as a function of the electric field for the present four systems, used for the prediction of the blend's PLQY_D:A,pred_ in eqn (2). All other parameters are given in Table 1. As shown in Fig. 6b, the predicted PLQY_D:A,pred_ (solid lines) shows excellent agreement to the experimental PLQY_D:A_ data (triangles) over the entire field range and for all of our model systems. To further check for the generality of our conclusions, we extended our approach to other NFA-based blend systems. For instance, we blended Y6 and Y5 acceptors with another donor polymer PTQ10, which, according to the data summarized in the Supplementary Note 4 (ESI†), has an IE *ca.* 0.12 eV on average higher than H_PM6_, rendering it a proper comparison with our L_PM6_-based blends.^27^ The absorption, photovoltaic performance, TDCF and TAS results for PTQ10:Y5 and PTQ10:Y6 are shown in detail in Fig. S17–S19 (ESI†). Indeed, the PTQ10:Y5 blend exhibits field-assisted free charge generation characteristics similar to the L:Y5 blend, despite the very different chemical structure and molecular weight of PTQ10 and L_PM6_. In addition, we tested our model on a H_PM6_ blended with BTPV-4F-eC9, a novel NIR absorbing Y-series NFA with extended absorption.^61^ This NFA was reported to have a smaller IE than Y6 and indeed, the H:BTPV-4F-eC9 blend exhibits a weak field-dependence of free charge generation and PL. Further details on this material system will be published in a later work. We also included data for the blend of high molecular weight PM6 with o-IDTBR, another NFA with a shallower IE than Y6, for which a field-dependence of free charge generation and exciton dissociation was previously reported.^11^

**Table 1:** Comparison of the measured PLQY_D:A_ and predicted PLQY_D:A,pred_ at the *V*_OC_ or zero field conditions. The latter is predicted from our model of singlet-decay limited free charge generation as outlined in the text. Also tabulated are the ELQY values used in eqn (2). The far right column row, obtained from the ratio of ELQY of the blend to PLQY of PS:NFA, denotes the probability that non-geminate free charge recombination (NGR) proceeds *via* the reformation and decay of the NFA singlet

in%	PLQY_D:A_	PLQY_D:A,pred_(*V*_OC_)	ELQY	Fraction of NGR *via* LE
H:Y6	5.2 × 10^−2^	5.6 × 10^−2^	4.0 × 10^−3^	0.4
L:Y6	0.42	0.42	3.5 × 10^−2^	3.4
H:Y5	1.07	1.13	0.22	8.9
L:Y5	2.03	2.12	0.13	5.3
PM6:o-IDTBR	4.20	4.77	0.20	3.2
PTQ10:Y6	0.11	0.14	9.9 × 10^−3^	1.0
PTQ10:Y5	2.01	2.41	0.38	15.5
PM6:BTPV-4F-eC9	0.28	0.28	4.6 × 10^−3^	0.9

Notably, for all these various blends exhibiting different ΔIE, we can closely describe their emission properties with our model which assumes free charge generation process that is primarily limited by singlet exciton decay, see Fig. 6b. To substantiate this, Table 1 shows the comparison of measured and predicted PLQY of the blend at *V*_OC_ conditions for all these tested OSCs. Interestingly, the contribution to the total emission from reformed LE states *via* NGR is very minor (see Fig. S20, ESI†). Accordingly, the ratio of ELQY_D:A_ to PLQY_PS:A_ in the right side column of Table 1 describes the likelihood that NGR occurs through emissive LE states, and this is found to be smaller than 10% (in most cases less than 5%). In fact, the main difference between LE dissociation into free charge and free charge recombination *via* LE states is that the latter process produces a significant density of triplet CT states, which can decay further to local triplet excitons through back electron transfer.^62^ Recent TA work suggested that free charge recombination in PM6:Y6 proceeds almost entirely through this dark pathway,^63^ with which our results indeed agree.

The critical role of LE splitting efficiency in blend emission is also evidenced by trPL. Fig. S21 (ESI†) shows the LE splitting efficiency as a function of applied bias for the Y5-based blends, obtained by integrating the trPL kinetics of each blend. If the reduction of PLQY_D:A_ with increasing reverse bias originates entirely from field-induced quenching of the singlet exciton emission, the trPL quenching efficiency as function of electric field should align perfectly with the PLQY_D:A_ data (see Supplementary Note 2 for description, ESI†). This is indeed the case and is shown in Fig. 6c for the two PM6:Y5-based blends. The trPL quenching of the Y5-based blends also show convergence at high external bias, which additionally confirms our conclusion that the loss channel *via* radiative decay of the LE state can be substantially circumvented at high field conditions, despite smaller ΔIE in the inefficient blends.

### (f) Marcus theory simulations with quantum chemical calculations

To quantitatively address the role of the IE offset on the efficiency of free charge formation, we solved the standard rate model for the steady state population of LE and CT states:^12,64^3a
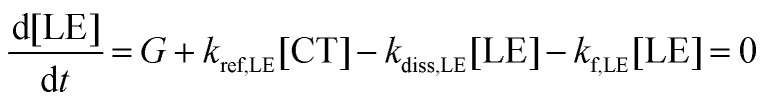
3b
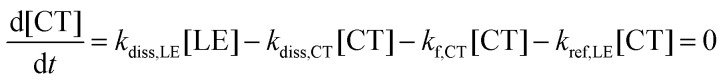


These quantities are illustrated in Fig. 8a. Here, [LE] and [CT] are the densities of LE and CT, respectively. *G* is the generation rate of LE states, *k*_f,LE_ and *k*_f,CT_ are the decay rates of LE and CT excitons, respectively, *k*_diss,LE_ and *k*_diss,CT_ are the dissociation rates of LE and CT excitons, respectively, and *k*_ref,LE_ is the reformation rate of LE states from CT states.

Since we are only concerned with the generation pathway, we exclude the reformation of CT states from free charge carriers through NGR. We then used Marcus theory to calculate the rates *k*_diss,LE_ and *k*_ref,LE_:4a
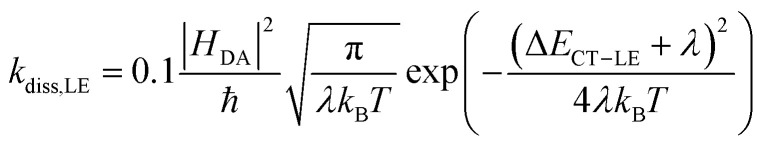
4b
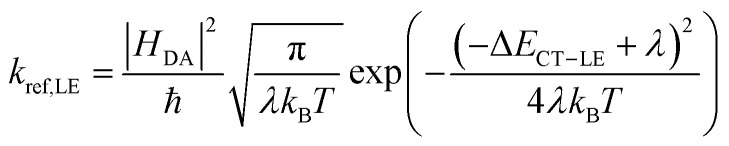
Here, |*H*_DA_| is the electronic coupling of the donor and acceptor for hole transfer, *λ* the corresponding reorganization energy, *k*_B_ the Boltzmann constant, and *T* the temperature. The energetics at the heterojunction are expressed by the energetic offset between the CT and LE states (Δ*E*_CT–LE_) which is related to the ΔIE in a first order approximation, as mentioned earlier. Following common practice, we assumed that the effective density of states of local excitons in our blends is ten times that of CT states,^14^ meaning that not every excited NFA has a neighbouring donor molecule to undergo charge transfer. This rationalizes the pre-factor 0.1 in eqn (4a). In contrast, every CT state has at least one neutral neighbouring NFA molecule available for LE reformation.

**Fig. 7:**
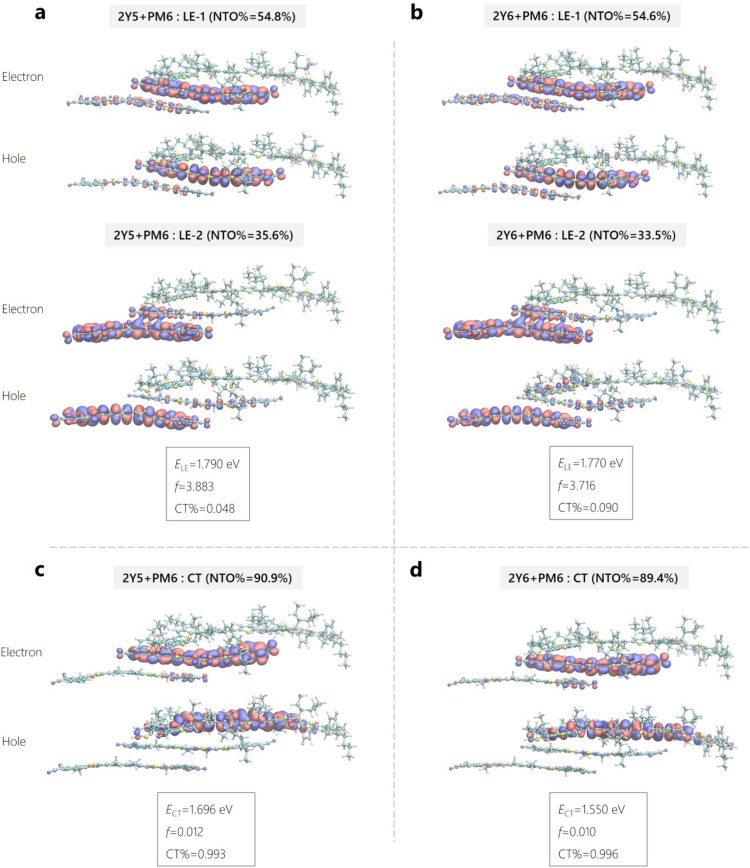
**Modelling of excited state energies for H**_**PM6**_ **:Y*x* geometries.** (a) and (b) Natural transition orbitals or NTOs (hole and electron) of the singlet local excited (LE) state in the 2Y5 + PM6 and 2Y6 + PM6 system. (c) and (d) NTOs of the charge transfer (CT) states for the same Y5- and Y6-based interfacial trimer systems. The two pairs NTOs of the LE state and one pair NTOs of the CT state are plotted, along with the corresponding weights (NTO%). The excitation energies (*E*_LE_/*E*_CT_), oscillator strength (f) and weight of CT character are provided as well.

To ascertain the values of |*H*_DA_| and the inner reorganization energy for charge transfer, quantum mechanical calculations were performed on model D:A interface systems using density functional theory (DFT). The computational modelling therein was also used to atomically resolve and compare the nature of the neutral LE and CT states of the interfacial systems and to determine the excited state energies. Here, the interface systems consist of either a single Y*x* molecule or an optimized Y*x* dimer stacked onto a PM6 oligomer (of two repeat units) with a stacking distance of 4.5 Å. Further details can be in found in Supplementary Note 3 (ESI†). Fig. 7 shows the natural transition orbitals (NTOs) of the electrons and holes of the excited states along with the excitation energies (*E*_LE_/*E*_CT_), oscillator strength (*f*) and weight of CT character (CT%) of the Y*x*-dimer/PM6 interfacial system (denoted here as an interfacial trimer or 2Y*x* + PM6). Fig. S22 and Table N3.1 (ESI†) provides the data for the corresponding interfacial systems with only one Y*x* molecule (interfacial dimer or Y*x* + PM6). The LE states of the interfacial trimer in Y5 and Y6-based systems in Fig. 7a and b have similar excitation energy, *E*_LE_ = 1.790 eV *vs. E*_LE_ = 1.770 eV, wherein the corresponding NTO hole and electron molecular orbitals are also very similar to each other. The predicted LE state energies are still much higher (>0.30 eV) than what we have found in the experiment. In our previous work, we nicely predicted the absorption spectra of Y6 aggregate, where the energy went from 2.06 eV for the Y6 monomer down to 1.48 eV for the Y6 aggregate.^65^ As we concluded before, this is mainly due to the delocalization effect of excitonic states in the Y6 aggregate. We can observe this effect in the NTOs LE state figures in Fig. 7a and b, where the molecular orbitals are delocalized to the next Y*x* molecule in the 2Y*x* + PM6 interfacial systems, compared to the Y*x* + PM6 systems in Fig. S21 (ESI†). As expected, and nicely shown by the comparison of the corresponding NTOs in Fig. 7 and Fig. S21 (ESI†), delocalization effects are much weaker for the CT states. The only difference between the two systems is the “F” atoms in Y6 molecules being replaced by “H” atoms to become Y5 molecules. The electron affinity difference of the two acceptor molecules also changes the electron population of the PM6, especially in the region near the extra fluorination site.

In detail, we find that the LE transition energy is about 25–30 meV larger for the Y5-based interfacial dimer and trimer systems compared to the Y6-containing systems, see Table N3.1 (ESI†). This is significantly smaller than the difference of about 100 meV in the peak position in absorption of the NFAs (see Fig. 1d and Fig. S2b, ESI†). We attribute this to the stronger tendency of Y6 to aggregate. The CT energy of the Y5-based aggregates is predicted to be more than 100 meV higher than the Y6 counterparts, see Table N3.1 (ESI†), which is consistent with the approximately 100 meV higher IE and EA energies and corresponds nicely to the almost 100 mV higher *V*_OC_ of the Y5-based blends (see Fig. 2c). Regarding Δ*E*_CT–LE_, the LE state lies above the CT state in all calculated systems, but the energy difference decreases significantly with aggregate size. The reason for this is the noted decrease of the LE energy with an increasing number of NFAs in the stack which is in contrast to the energy of the CT state which changes only little with aggregate size. We, therefore, expect Δ*E*_CT–LE_ to eventually tend closer to zero or to even adopt positive values in a real D:A aggregate, especially in the low-offset blends.

Table 2 summarizes all parameters which were used as constants in the steady-state rate model. The reorganisation energy *λ* consists of the inner and outer reorganization energy, *λ*_*i*_ and *λ*_*a*_, respectively, where the former is mainly related to the change of the molecular geometry, while the latter originates from the change in the polarization of the environment during charge transfer. Values for *λ*_*i*_ were obtained from computational calculations (see Supplementary Note 3 and Table N3.2 for more details, ESI†) and are similar for both Y5- and Y6 aggregates (*ca.* 325–330 meV). Recent work predicted a reorganization energy for Y6 LE dissociation of only 0.108 eV, but the time-dependent DFT calculation considered only one Y6 molecule in combination with an PM6 oligomer and, also, the molecular alignment was different in their case.^66^ The value of *λ*_*a*_ (≅ 250 meV) was taken from literature.^67^ This yields *λ* = *λ*_*i*_ + *λ*_*a*_ of *ca.* 580 meV. This value is comparable to recent reports of *λ* for a PBDB-T/ITIC dimer.^68^ Note that contrastingly smaller values for the inner (161 meV) and outer (150 meV) reorganization energy were predicted for a J61:m-ITIC dimer.^69^ We, therefore, varied *λ* between 300 and 600 meV in steps of 50 meV in the rate-model simulations. |*H*_DA_| was set equal to 10 meV based on the average of the results from DFT. The emissive decay rates *k*_f,LE_ and *k*_f,CT_ were both set equal to 1 × 10^9^ s^−1^, a typical value for our and other state of the art NFAs^14^ and for the CT decay time,^70^ respectively. Note that much higher decay rates were measured and predicted for the decay of the interfacial triplet CT *via* back-electron-transfer (BET) to the NFA triplet.^63,66^ However, dissociation of the photogenerated LE generates only singlet CT excitons, and geminate CT recombination *via* BET was shown to be insignificant. Given the lack of recombination through the CT state during free charge formation, rapid CT dissociation was assumed, which is driven, for example, by band bending at the DA heterojunction.^12,71^

**Table 2:** Fixed parameters used in the steady-state model for LE and CT populations

Parameter		Value
Decay rate of NFA LE states	*k*_f,LE_ [s^−1^]	1 × 10^9^
Decay rate of CT states	*k*_f,CT_ [s^−1^]	1 × 10^9^
Dissociation rate of CT states	*k*_diss,CT_ [s^−1^]	1 × 10^11^
Generation rate of LE states	*G* [m^−3^ s^−1^]	1 × 10^28^
Total reorganization energy	*λ* [eV]	0.3 to 0.6
D:A electronic coupling for hole transfer	|*H*_DA_| [eV]	0.01

**Fig. 8:**
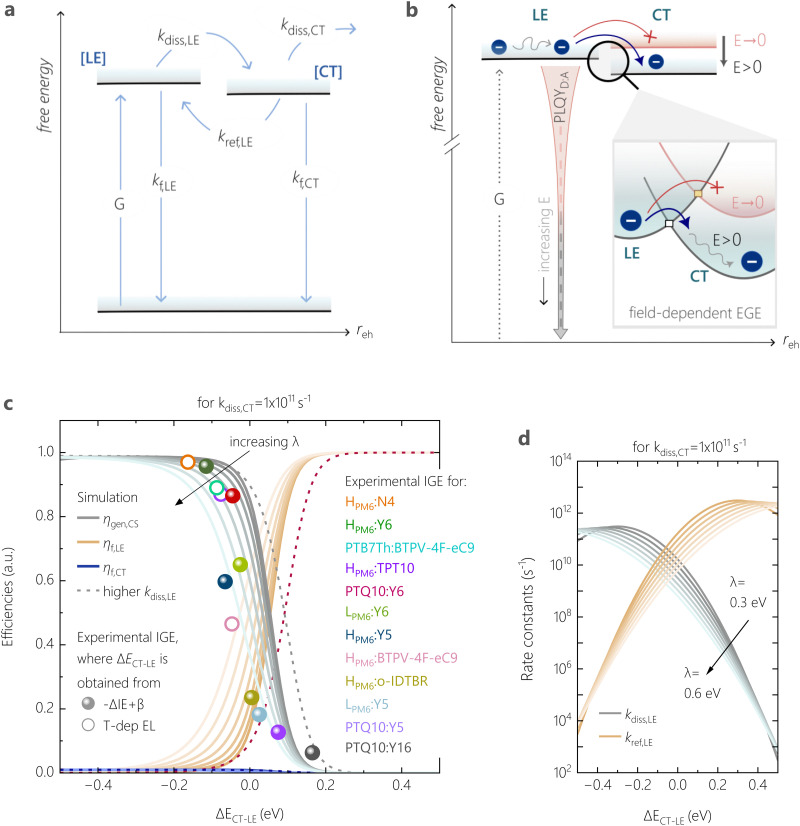
**Marcus theory used to explain singlet exciton decay as the competing pathway to free charge generation.** (a) Rate model showing the various transitions possible for excitons prior to free charge formation. (b) A representation of charge generation, *i.e.* LE dissociation, based on Marcus theory. (not to scale) The dark blue circles depict bound charge carriers in their excitonic states, and energetic states under no field (applied field) are described with red (dark grey) lines and curves, respectively. The barrier for LE-to-CT formation is explained by the Marcus type energy picture at the interface, highlighted in the box, and is the key step limiting EGE. The squares on the potential curves denote the cross-over point between LE and CT energetic potentials, and signify the activation barrier that bound charge carriers in vibrationally relaxed LE states must overcome to form the CT state – a barrier that is lowered under the influence of an effective field. (c) The dependence of free charge generation efficiency, losses *via* the decay of the local NFA exciton and the CT state (*η*_gen,CS_, *η*_f,LE_, *η*_f,CT_, respectively) on Δ*E*_CT–LE_, simulated at zero-field from the steady-state rate model assuming not all photogenerated excitons are able to undergo charge transfer. The simulated data is shown for varying reorganisation energies *λ*. Overlaid are the experimental generation efficiencies (IGE) of various tested blends measured at open-circuit conditions, as a function of Δ*E*_CT–LE_. The dotted lines describe the simulated efficiencies if the singlet dissociation rate were not reduced (*i.e.*, prefactor = 1 in eqn (4a)). (d) Energy-offset dependence of the rate constants of LE dissociation and LE reformation from CT states, for the same CT dissociation rate *k*_diss,CT_ as in (c), and for varying *λ*.

The populations [LE] and [CT], obtained from eqn (3a) and (3b) and the parameters in Table 2 were used to calculate three efficiencies according to eqn (5a)–(5c) in steady-state condition:5a
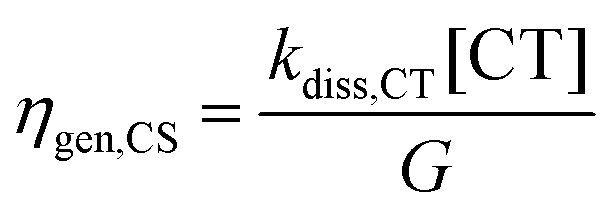
5b
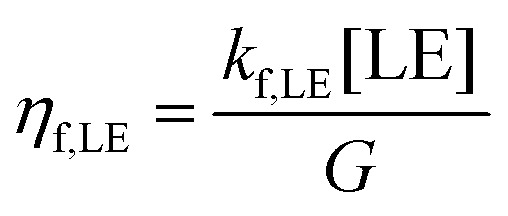
5c
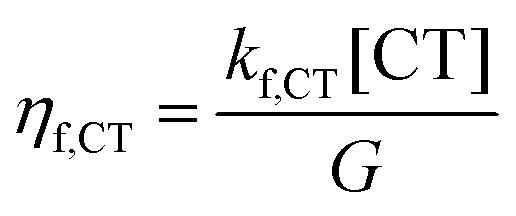
Here, *η*_gen,CS_ stands for the free charge generation efficiency while *η*_f,LE_ and *η*_f,CT_ describe the losses *via* the decay of the NFA LE state and the CT state, respectively. These three efficiencies are plotted against Δ*E*_CT–S1_ in Fig. 8c, for a *k*_diss,CT_ of 1 × 10^11^ s^−1^. We observe a sharp decrease in *η*_gen,CS_ when the CT state moves above the LE state (Δ*E*_CT–LE_*> 0*), as also shown by other works.^12,64^ We note two other findings that are important here. First, LE decay is shown to be the main competing channel to free charge generation. This is a direct consequence of the choice of a high CT dissociation rate, as shown in Fig. S23 (ESI†). Second, there exists a critical Δ*E*_CT–LE_ above which *η*_gen,CS_ is a strong function of *λ*. Interestingly, the overall dependence of *η*_gen,CS_ on Δ*E*_CT–LE_ and *λ* is similar to that of *k*_diss,LE_, which is shown with grey lines in Fig. 8d. To understand this, we analysed the analytical steady state solution of eqn (3a) and (3b) in the ESI† for different cases (please refer to Supplementary Note 5 for further discussion and explanation, ESI†). It is exactly for the case of fast CT dissociation (case 1 in the ESI† Appendix Note 5) that *η*_gen,CS_ is determined by *k*_diss,LE_, which is a strong function of *λ* through eqn (4a). On the other hand, if CT dissociation is slow, a kinetic equilibrium between the CT and the LE population is established, where [CT]/[LE] is governed by *k*_diss,LE_/*k*_reff,LE_ (case 2 in the ESI† Appendix Note 5). Since *k*_diss,LE_/*k*_ref,LE_ is independent of *λ* (see Fig. S24, ESI†), the influence of *λ* on *η*_gen,CS_ becomes reduced, as shown in Fig. S22a (ESI†). Finally, if the exciton dissociation rate increases and dominates over the LE decay rate (*i.e. k*_diss,LE_ + *k*_f,LE_ ≅ *k*_diss,LE_), the CT dissociation probability determines *η*_gen,CS_, albeit with a smaller influence of *λ* (case 3 in Appendix Note 5 and Fig. S25, ESI†).

Related to this, the question arises whether *k*_diss,LE_ can be deliberately tuned to optimize device performance for a given Δ*E*_CT–LE_. As discussed earlier for eqn (4a), the number of available CT sites for charge generation is another factor determining *k*_diss,LE_; an overall higher *k*_diss,LE_ will permit efficient exciton dissociation despite a small *E*_CT–LE_ (see Fig. S25, ESI†). However, recent work suggested that an enhanced *k*_diss,LE_ increases non-radiative voltage losses, as it promotes recombination *via* the highly non-emissive CT decay pathway.^55^ The authors proposed a ternary blend approach where a second NFA with a very small IE offset to the donor is added to reduce *k*_diss,LE_. Further work will be devoted to the question whether such *k*_diss,LE_ tuning is possible also in binary blends, for instance through chemical engineering.

To compare with experimental values, we considered IGE at *V*_OC_ (as measured by mTDCF) as the free generation efficiency *η*_gen,CS_. The proper determination of Δ*E*_CT–LE_ requires great care due to the disperse energy values reported in literature. Our approach is described in detail in the Supplementary Note 4 (ESI†). In short, for all systems where trustable values for ΔIE existed, we used a constant scaling factor *β* so that Δ*E*_CT−LE_ = −ΔIE + *β*. Such scaling factors were used in the past to account for differences between the LE and CT binding energy but also possible band-bending which lifts *E*_CT_ relative to *E*_LE_.^9,72^ Here, *β* was set to 0.235 eV to reproduce an Δ*E*_CT–LE_ of −0.115 eV for H:Y6, which was determined previously from temperature-dependent ELQY.^33^ This value is also in very good agreement with a recent time-dependent DFT-based prediction for PM6:Y6 (Δ*E*_CT–LE_ = −0.121 eV).^66^ We flanked the data set with a very poorly performing blend namely PTQ10:Y16^27^ (see Fig. S26 for *JV* and TDCF data, ESI†) and an efficient blend with no field-dependence of photocurrent generation namely PM6:N4.^73^ For PM6:N4 as well as blends of H_PM6_ with TPT10 and BTPV-4F-eC9, Δ*E*_CT–LE_ was measured directly using temperature-dependent EL (see Fig. S27, ESI†).

Fig. 8c shows an excellent agreement when overlaying the experimental IGE values with the simulation result. (See Fig. S28 (ESI†) for the overlay with experimental emission, where we considered the measured PLQY_D:A_(*V*_OC_)/PLQY_PS:A_ as the radiative decay efficiency of LE states) In particular, the predicted drop in *η*_gen,CS_ and the rapid increase in *η*_rec,LE_ upon the small variation in Δ*E*_CT–LE_ (by 200 meV) is especially consistent with the experimental anticorrelation between the blends’ emission and free charge generation data observed earlier. Our combined experimental-simulation work shows that a Δ*E*_CT–LE_ of *ca.* −0.1 V, corresponding to ΔIE of *ca.* 0.35 eV, is needed for efficient free charge generation against the competition with LE recombination. This is a direct consequence of the dominant role of the charge generation rate *k*_diss,LE_ and it is related to Δ*E*_CT–LE_ and also to *λ*.

Our model also explains the pronounced effect of the electric field on the exciton spitting efficiency in our low-offset systems. If the CT state can be easily polarized, it stands that an external field stabilizes the CT state thereby lowering its energy relative to that of the LE state,^74^ as illustrated in the inset of Fig. 8b. This diminishes the uphill barrier to CT formation and promotes free charge generation by facilitating LE dissociation. Consequentially, the radiative decay *via* NFA LE states depends on the ability of such photogenerated LE states to contribute to free charge generation. This charge transfer process enabled by the internal field directly explains the PLQY trends predicted for different external bias, and is likely the key factor impacting photocurrent in low-offset NFA-based OSCs.

Finally, we would like to address the role of morphology. Numerous studies have shown that charge separation benefits from a well phase-separated structure with rather pure and possibly well-crystallized domains of the donor and acceptor components.^32,75,76^ It has been previously suggested that such a morphology reduces the CT binding energy either through delocalization of the interfacial CT state, or by providing a larger driving force to counteract the mutual Coulombic attraction of the electron–hole pair towards the charge separated state.^12,32,74,77,78^ However, charge separation does not appear to be a major obstacle in the systems investigated here, as evidenced from the anticorrelation of TDCF and PL data, TA measurements and analytical simulations. On the other hand, charge generation takes place on a smaller length scale. Thus, although we acknowledge that the intermolecular order and orientation at the D:A heterojunction can affect critical parameters for LE–CT interactions,^48,79,80^ ΔIE (Δ*E*_CT–LE_) seems to be the most important property for the free charge generation process. This is confirmed by recent studies on planar heterojunction (PHJ) devices. For example, Wang *et al.* varied the aggregation (and orientation) in the donor or in the acceptor layer of IT-4F-based PHJ devices.^72,81^ Both studies provided strong evidence for a direct correlation between the efficiency of free charge generation and the LE–CT energy offset Following the same line of arguments, Nakano *et al.* reported a pronounced field-dependence of free charge generation in PM6/Y6 PHJ devices which was absent in the corresponding BHJ devices.^82^ Based on optical spectroscopy and temperature-dependent *V*_OC_ measurements, the authors concluded a smaller Δ*E*_CT–LE_ for the bilayer device, consistent with the conclusions from our work. On the other hand, the pronounced effect of polymer chain orientation on the BHJ device performance was attributed to charge separation, where a preferential end-on orientation of polymer chains relative to the planar heterojunction increases the delocalization of the charge pair in the CT state normal to the DA interface and thereby renders CT dissociation more efficient.^82^ Further studies with a detailed investigation of the free charge generation process are certainly needed to substantiate these conclusions. Nevertheless, the systematic dependence of the device performance on ΔIE (Δ*E*_CT–LE_) as reported across a large set of NFA-based systems, in both planar heterojunctions and bulk heterojunctions, with very different morphologies, suggests Δ*E*_CT–LE_ as the determining factor in low-offset OSCs.^12,13^

## Conclusion

To conclude, our simulations and experiments consistently reveal a large effect of the IE offset but also of the electric field, on the free charge generation efficiency in low-offset OSCs. Our key finding is that as the energy offset reduces, the decay of NFA singlet excitons becomes the primary and direct competition to free charge generation. We also learn that the IE offset of PM6:Y6 is close to the limit of the region where the exciton dissociation efficiency is close to one. In agreement to this, all state-of-the-art high efficiency binary blend devices have been based on the combination of Y6 (or derivatives of this NFA with slightly different chemical structure but similar IE) with PM6, D18 or polymers with related chemical structure and energetics.^1,83,84^ Therefore, our conclusion also encourages further material design along similar lines and suggests that any attempts to further reduce the IE offset in the blend will lead to an unavoidable loss in free charge generation efficiency. However, if the IE offset is in fact reduced, then Marcus theory provides guidelines so that the solar cell could still balance high photovoltage with high photocurrent. For example, a reduction of the reorganization energy for charge transfer will enable efficient free charge generation even for Δ*E*_CT–S1_ close to zero (in Fig. 7c, corresponding to ΔIE of *ca.* 240 meV). For example, Zhong *et al.* reported sub-ps charge transfer in ITIC-based donor:NFA blends which was related to the small reorganization energy for charge transfer.^69^ In addition, a smaller reorganization energy for hole back transfer will reduce the non-radiative voltage loss due to electron-vibration coupling.^85^ Recent works have demonstrated the power of these strategies to obtain highly efficient single-junction OSCs albeit with smaller ΔIEs than in PM6:Y6.^86,87^

## Data availability

The data that support the findings of this study are available within the article and its ESI.†

## Conflicts of interest

There are no conflicts of interest to declare.

## Supplementary Material

EE-017-D4EE01409J-s001
