# Supplementary material for: On the critical competition between singlet exciton decay and free charge generation in non-fullerene based organic solar cells with low energetic offsets
Source: Energy Environ Sci. 2024 Jul 30;17(18):6676–97. doi: 10.1039/d4ee01409j (PMC11323475; doi:10.1039/d4ee01409j)
Supplement: EE-017-D4EE01409J-s001 [file EE-017-D4EE01409J-s001.pdf]

Supplementary Information (SI) for Energy & Environmental Science.
This journal is © The Royal Society of Chemistry 2024

Supporting Information

On the critical competition between singlet exciton decay and free charge generation in non-fullerene based organic solar cells with low energetic offsets

Manasi Pranav[1], Atul Shukla[1], David Moser[2], Julia Rumeney[2], Wenlan Liu[3], Rong Wang[4], Bowen Sun[1], Sander Smeets[5,6], Nurlan Tokmoldin[1,7], Yonglin Cao[1], Guorui He[1], Thorben Beitz[1], Frank Jaiser[1], Thomas Hultzsch[1], Safa Shoaee[1,7], Wouter Maes[5,6], Larry Lüer[4], Christoph Brabec[4,8], Koen Vandewal[5,6], Denis Andrienko[3], Sabine Ludwigs[2], Dieter Neher[1*]

Affiliations:

[1] *Institute of Physics and Astronomy, University of Potsdam, Karl-Liebknecht Straße 24/25, 14476 Potsdam, Germany*

[2] *IPOC – Functional Polymers, Institute of Polymer Chemistry, University of Stuttgart, Pfaffenwaldring 55, 70569 Stuttgart, Germany*

[3] *Max Planck Institute for Polymer Research, Ackermannweg 10, 55128 Mainz, Germany*

[4] *Institute of Materials for Electronics and Energy Technology (i-MEET), Friedrich-Alexander-Universität Erlangen-Nürnberg, Martensstrasse 7, Erlangen 91058, Germany*

[5] *UHasselt—Hasselt University, Institute for Materials Research, (IMO-IMOMEC), Agoralaan 1, 3590 Diepenbeek, Belgium*

[6] *IMOMEC Division, IMEC, Wetenschapspark 1, 3590 Diepenbeek, Belgium*

[7] *Heterostructure Semiconductor Physics, Paul-Drude-Institut für Festkörperelektronik, Leibniz-Institut im Forschungsverbund Berlin e.V, Hausvogteiplatz 5-7, 10117 Berlin, Germany*

[8] *Helmholtz-Institut Erlangen-Nürnberg for Renewable Energies (HIERN), Forschungszentrum Jülich, Immerwahrstraße 2, 91058 Erlangen, Germany*

*Corresponding author: neher@uni-potsdam.de

Experimental methods

Fabrication:

For Y5-based blends, the commercial polymer PM6 with high molecular weight and the small non-fullerene acceptor Y5 were both purchased from 1-Materials Inc. These particular batches and suppliers were chosen because they provided the best device performance with the device structure ITO/PEDOT:PSS/D:A/PDINN/Ag for all material combinations tested. For Y6-based blends, the highest PCE was achieved for PM6 and NFA purchased from Brilliant Matters Inc. The low molecular weight PM6 synthesis was conducted on a custom-made continuous flow system. Exact synthesis conditions have been reported previously.[1] Flow rates were set to achieve a residence time of 1.6 min to obtain the targeted number-average molar mass (M_n) of 3.5 kg/mol.

Although optimised PM6:Y6 and PM6:Y5 (with high M_n) devices are typically fabricated with a PDINO electron transport layer,[2] we opted to use PDINN for our studies. While this leads to lower PCEs, PDINN possesses higher conductivity due to doping[3], which enables shorter dielectric relaxation times and a faster response of the device in transient optoelectronic measurements, such as TDCF. Devices were fabricated in a conventional geometry with a structure ITO/PEDOT:PSS/D:A/PDINN/Ag. Glass substrates with pre-patterned ITO (Lumtec) were cleaned in an ultrasonic bath with acetone, Hellmanex III, deionized water and isopropanol for 10 minutes each, followed by microwave oxygen plasma treatment (4 min at 200 W). Subsequently, an aqueous solution of PEDOT:PSS (Heraeus Clevios™ PEDOT:PSS) was filtered through a 0.2 µm PTFE filter and spin coated onto ITO at 5000 rpm under ambient conditions. The PEDOT:PSS coated substrates were thermally annealed at 150 °C for 15-20 min. Blends solutions were prepared to a total concentration of 14 mgmL^{-1} (17 mgmL^{-1} for low M_n-based blends) using a $CHCl_3$ solvent (purchased from Carl Roth), with a 1:1.2 weight ratio. The solution was stirred for 3 hours inside a nitrogen-filled glovebox. Polystyrene (Sigma Aldrich) blends with the NFAs were prepared similarly, with similar concentration and blend ratio as the D:A blends. The blend was spin coated onto the PEDOT:PSS coated substrates using the optimized spin speed for obtaining a photoactive layer of thickness of 100-110 nm. Y6-based blends were annealed at 100 °C for 10 min to improve the performance. Then, a 1 mgmL^{-1} solution of PDINN (1-Material Inc) in methanol (Sigma Aldrich) was spin coated at 1500 rpm. Finally, 100 nm of silver as the top electrode was evaporated under a 10^{-6}-10^{-7} mbar vacuum. The resulting active area was 1 mm^2 for TDCF and steady state biased-PL measurements, and 6 mm^2 for EQE_{PV}, JV, EL, ELQY, and TrPL measurements.

For PLQY measurements, glass substrates with full-area ITO were used to produce ca. 1 cm^2 device area, to ensure that the entire device stack is illuminated by the incident beam. For spectroelectrochemistry, the photoactive blends were spin coated on full-area ITO glass subtrates at reduced blend concentrations to obtain films of ca. 30 nm thickness. The preparation method for PM6:o-IDTBR OSCs has been previously reported.[4]

Current density-voltage characteristics (JV)

JV curves were measured using a Keithley 2400 SourceMeter in a 2-wire source configuration. Simulated AM1.5G irradiation at 100 mWcm-2 was provided by a filtered Oriel Sol2A Class AA Xenon lamp and the intensity was monitored simultaneously with a Si photodiode. The sun simulator is calibrated with a KG5 filtered silicon solar cell (certified by Fraunhofer ISE).

EQE_{PV} and absorbance

The EQE_{PV} was measured with broad white light from a 300 W Halogen lamp (Phillips) which was chopped at 80 Hz (Thorlabs MC2000), guided through a Tornerstone monochromator and coupled into an optical quartz fibre, calibrated with Newport Photodiode (818-UV). An SR 830 Lock-In Amplifier measures the response of the solar cell under different bias voltages applied by a Keithley 2400.

Absorbance was measured on films coated under the same fabrication conditions mentioned above, but on glass substrates with a Varian Cary 5000 spectrophotometer in transmission mode.

Bias dependent photoluminescence (PL) and absolute PL/EL (PLQY/ELQY)

Bias-dependent PL measurements were performed using a 520 nm CW laser diode (Insaneware) for steady state illumination, and the intensity of the laser was adjusted to a 1 sun equivalent by

illuminating a PM6:Y6 solar cell under short-circuit (provided by a Keithley 2400) and matching the current density reading to the J_{SC} obtained in the sun simulator. The excitation beam was focused onto the sample using a stage of mirrors and lenses. Bias voltages ranging from open-circuit voltage to -8 V were applied to the sample using the same Keithley 2400. To ensure that only the active layer is illuminated and contributes to the emission response, we masked the measured pixels. The emission spectra were recorded with an Andor Solis SR393i-B spectrograph with a silicon DU420A-BR-DD detector and an Indium Gallium Arsenide DU491A-1.7 detector. A calibrated Oriel 63355 lamp was used to correct the spectral response. PL spectra were recorded with different gratings at central wavelengths of 800, 1100, and 1400 nm, and merged afterwards. For PLQY measurements, the same laser was used but the excitation beam was channelled through an optical fibre into an integrating sphere containing the sample. A second optical fibre was used from the output of the integrating sphere to the Andor Solis SR393i-B spectrograph. The spectral photon density was obtained from the corrected detector signal (spectral irradiance) by division through the photon energy ($h\nu$), and the photon numbers were calculated from the numerical integration, using a Matlab code.

For absolute EL measurements, a calibrated Si photodetector (Newport) connected to a Keithley 485 picoampere meter was used. The detector, with an active area of $\sim$2 cm^2, was placed in front of the measured pixel with a distance <0.5 cm, and the total photon flux was evaluated considering the emission spectrum of the device and the external quantum efficiency of the detector. The injected current was monitored with a Keithley 2400.

For temperature-dependent EL measurements, regular EL measurements were conducted on the sample placed inside a cryostat. The temperature within the cryostat was controlled with liquid nitrogen and a LakeShore (Model 335) cryogenic temperature controller, ranging from 295K down to 180K-200K. The injected current was kept constant for each temperature, and was calculated from the J_{SC} at 1 sun.

Time-delayed collection field (TDCF) and modified-TDCF (mTDCF)

In TDCF, the device was excited with a laser pulse from a diode pumped, Q-switched Nd:YAG laser (NT242, EKSPLA) with ~6 ns pulse duration at a typical repetition rate of 500 Hz. An Agilent 81150A pulse generator was used to apply a square voltage transient waveform constituting the pre-bias V_{pre} and collection bias V_{coll}. To compensate for the internal latency of the pulse generator, the laser pulse was delayed and homogeneously scattered in an 85 m long silica fiber (LEONI) after triggering a photodiode. The device was illuminated while held at different pre-bias V_{pre}. After a pre-set delay time (calculated from the falling slope of the photodiode trigger), a high reverse bias V_{coll} was applied to extract all the charges generated in the device. V_{pre} and V_{coll} were sent by the Agilent 81150A pulse generator through a home-built current amplifier, which was triggered by a fast photodiode (EOT, ET-2030TTL). The current flowing through the device was measured via a 10 Ω resistor in series with the sample and recorded with an oscilloscope (Agilent DSO9104H). To avoid non-geminate recombination of photogenerated free charge carriers prior to extraction, the intensity of light is kept very low and the delay time of collection is set to ~1ns.

For mTDCF, a square-type waveform was programmed and fed into the Agilent 81150A pulse generator, and the delay parameters for the V_{pre} and V_{coll} voltage steps were pre-defined prior to the measurement, such that the V_{pre} was applied to the device for a duration equalling the RC time of the device plus the laser slew rate. As in classical TDCF, the laser beam incidence is delayed to compensate for the internal latency of the function generator.

Spectroelectrochemistry

The spectroelectrochemical measurements were taken place with a three-electrode setup under argon atmosphere. The blends with ITO coated glass as base substrate were used as working electrode, a platinum wire as counter electrode and a silver chloride covered silver wire as pseudo reference electrode. All the potentials were referenced to the ferrocene/ferrocenium (Fc/Fc+) redox potential, added after the measurements. The electrochemical cell contain MeCN as solvent and TBAPF6 (0.1 molar) as conducting salt as standard electrolyte. The UV-vis measurements were performed in transmission mode through the working electrode. For each data point in the cyclic voltammogram, a spectrum was measured. The potentials were applied by an PGSTAT204 potentiostat, purchased by Metrohm. The UV vis spectra were measured by a spectrometer system purchased by Zeiss, containing a MCS621 vis II detector and a CLH600 F halogen lamp.

Transient absorption (TA) spectroscopy

The femtosecond transient spectroscopy measurements were performed using a home-built setup. The output of an amplified Ti: Sapphire laser (Libra, Coherent, 800 nm, 1 KHz) was coupled to an Optical Parametric Amplifier (Opera Solo, Coherent) to generate the pump laser pulses (50 fs, 720 nm) which were chopped at □/2 (500 Hz). A portion of the fundamental laser beam was focused to an undoped YAG crystal to generate a broadband white light continuum. The probe pulses were spectrally dispersed using prism spectrometers and then collected using either a CMOS camera (visible spectral rang) or InGaAs photodiode arrays (IR spectral range). The probe continuum beam was split using a broadband beam splitter before the sample to monitor probe intensity on a shot-to-shot basis to minimize the impact of pulse-to-pulse fluctuation. The pump-probe polarization was set to magic angle (54.7°) to avoid orientational effects. The sequential probe shots corresponding to the pump on versus off were used to calculate the differential absorption signals. The pump-probe time delays up to ~7 ns are produced via a retroreflector connected to a computer controlled translational stage. In typical measurements, 6000 shots were averaged at each time delay and were repeated for at least 3 scans. The data saved as .dat files were processed via MATLAB for background and chirp correction. All the measurements were carried out under a nitrogen environment to prevent degradation from air contact.

Time-resolved photoluminescence (trPL)

Photoluminescence decay and quenching in pristine and BHJ films were recorded using Picoquant Fluotime 300. A 402 nm pulsed laser was used to excite polymer donors and a 783 nm pulsed laser was used for Y5 and Y6 acceptors. The emission signals were detected at the wavelength with the maximum steady PL peak using a single-photon counting PMT. Data fitting was performed using the internal software EasyTau2. For the field dependent TrPL measurement, a holder was built to connect the device samples to a Keithley digital source meter to control the applied bias voltage from 0 V to –12 V.

Computational modelling

We first set up a series of Y6 + Y6 + PM6 model trimer systems, where the Y6 dimer geometry was extracted from the optimized Y6 crystal structure [cite liu2023], and the PM6 (two repeat units) geometry was optimized in gas phase at the camb3lyp/6-311g(d,p) level of theory. The trimer systems

were constructed by placing the Y6 dimer and PM6 in a pi-pi stacking manner with the intermolecular distances (PM6 to the nearest Y6 molecule) 4.5 Å. We then optimized Y5 single molecule in gas phase at the camb3lyp/6-311g(d,p) level of theory. As the Y5 and Y6 molecules are very similar in their chemical formations, we utilized the Y6+Y6+PM6 trimer geometries, and only replace each Y6 molecule into the optimized Y5 molecules to set up the Y5+Y5+PM6 model trimer system. The Y6+PM6 and Y5+PM6 dimer systems are formed by simply removing the edge Y6 or Y5 molecule respectively, from the corresponding trimer systems.

The adiabatic excited states of all the system are calculated at the omega-tuned camb3lyp/6-311g(d,p) level of theory. We then performed the diabatization analysis for all the systems. The electronic coupling strengths were calculated with the diabatization scheme from ref[5] at the same level of the excited state calculation. Further details on analysis are provided in Supplementary Note 3.

Supplementary data

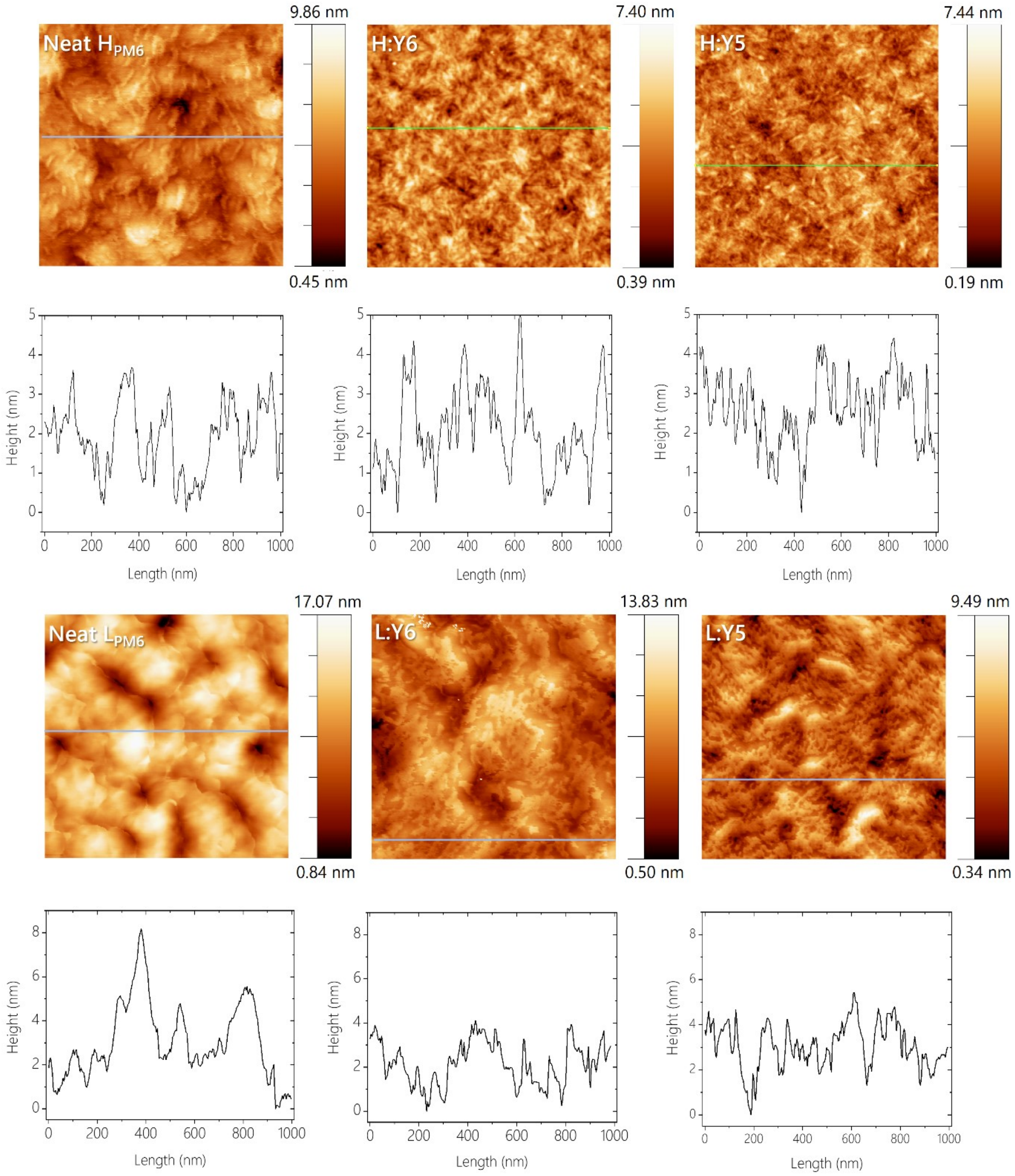

Figure S1. AFM heights images and corresponding line profiles for (top section) neat films of high molecular weight PM6 and the blends with Y6 and Y5 and (bottom section) the corresponding samples with low molecular weight PM6. The scan range was $1\ \mu m \times 1\ \mu m$ in all cases.

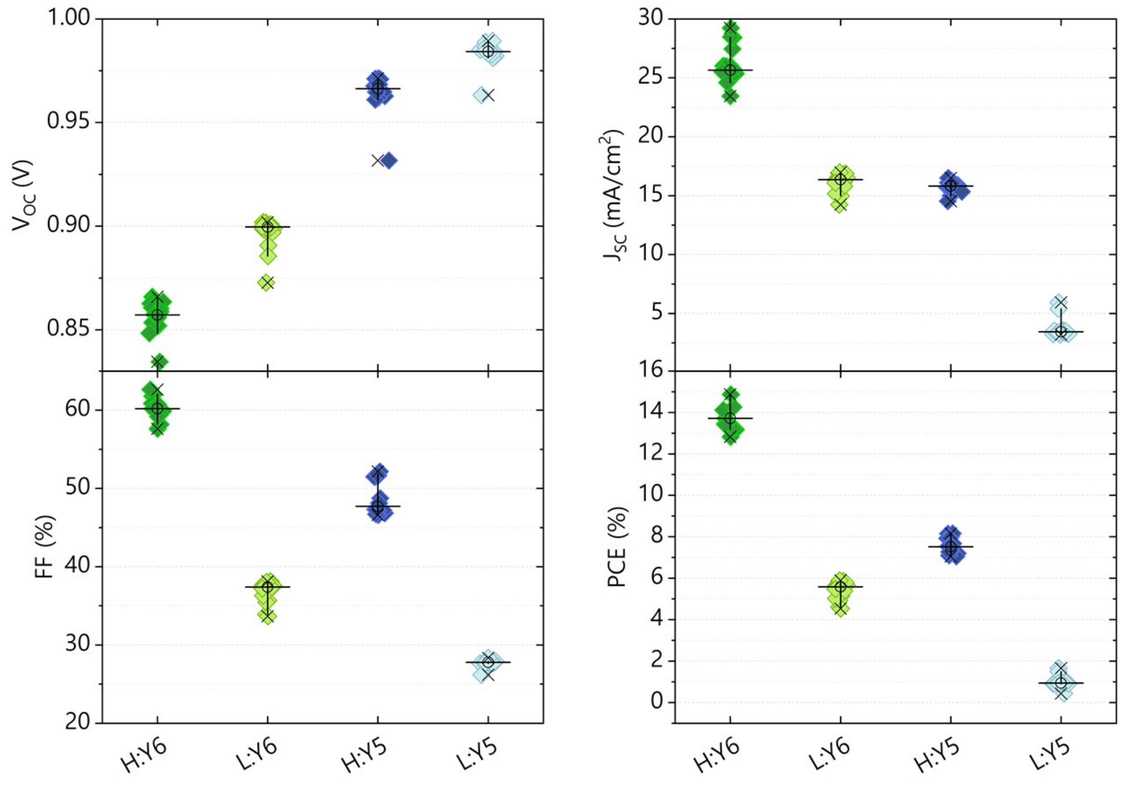

Figure S2. *Statistics of the photovoltaic parameters obtained from current-voltage measurements under simulated AM1.5G illumination of OSC devices with high molecular weight PM6 (H) and low molecular weight PM6 (L) with Y5 and Y6 non-fullerene acceptors. The statistics were obtained from 12 to 18 devices with a device are of 0.06 cm^2, and an active layer thickness of 100-110 nm.*

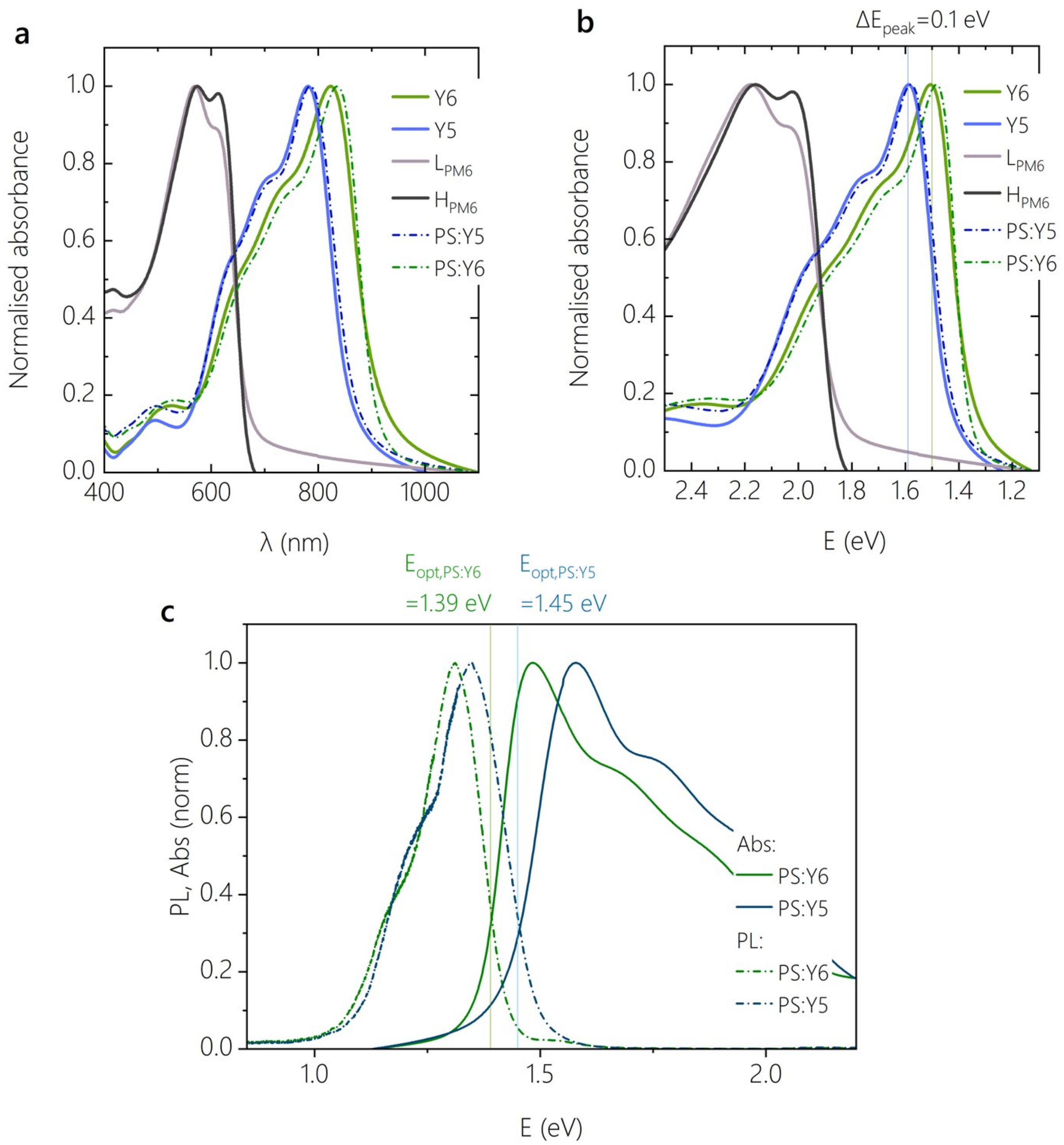

***Figure S3**. The normalised absorption spectra of the neat films of H_{PM6}, L_{PM6} and the Yx acceptor components (as neat films and as dispersed in a 1:1.2 ratio in a polystyrene, PS, matrix), plotted as a function of (a) wavelength and (b) photon energy. The reduced polymer aggregation in L_{PM6} is seen from a reduced 0-0 to 0-1 peak ratio. (c) Normalised absorption and photoluminescence spectra of the PS:NFA films, representing the higher optical bandgap for Y5.*

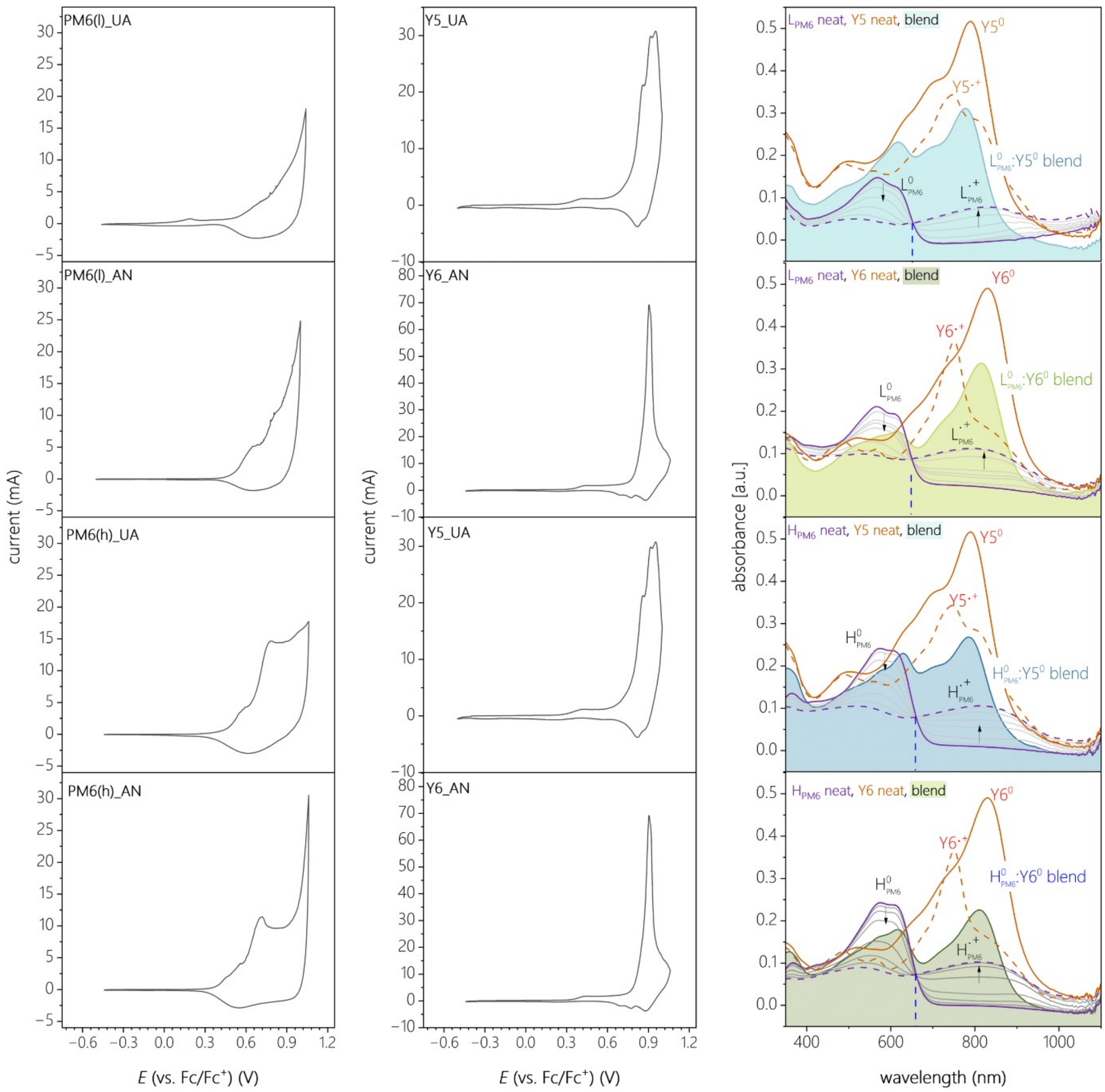

Figure S4. *(left and center) CV of neat films of PM6 polymers of different molecular weight, as well as that of neat Y5 and Y6. (right) Absorption spectra recorded during cyclic voltammetry of the neat polymer and NFA films, and the corresponding blends in neutral form (at 0.00 V vs Fc/Fc+). The dotted lines denote the absorption spectra of the radical cationic species formed at higher potentials on each of the neat components L_{PM6} and Y5.*

neats			
	**N**	$R^{\cdot+}$	**iso**
^{L}PM6	570 nm / 620 nm	815 nm	660 nm
^{H}PM6	570 nm / 610 nm	815 nm	660 nm
**Y5**	787 nm	751 nm	-
**Y6**	830 nm	751 nm	-
**L:Y5 blend**			
	**N**	$R^{\cdot+}$	**iso**
**PM6**	620 nm	650 nm - 780 nm	650 nm
**Y5**	780 nm	751 nm	-
**L:Y6 blend**			
	**N**	$R^{\cdot+}$	**iso**
**PM6**	610 nm	650 nm - 814 nm	650 nm
**Y6**	814 nm	751 nm	-
**H:Y5 blend**			
	**N**	$R^{\cdot+}$	**iso**
**PM6**	630 nm	650 nm - 785 nm	660 nm
**Y5**	780 nm	751 nm	-
**H:Y6 blend**			
	**N**	$R^{\cdot+}$	**iso**
**PM6**	620 nm	650 nm - 814 nm	660 nm
**Y5**	814 nm	751 nm	-

Figure S5. A view of the spectral positions of characteristic bands of neutral and radical cation species of the donor and acceptors, as measured in neat films as well as blend films. Also highlighted is the isosbestic point of the polymer spectral evolution.

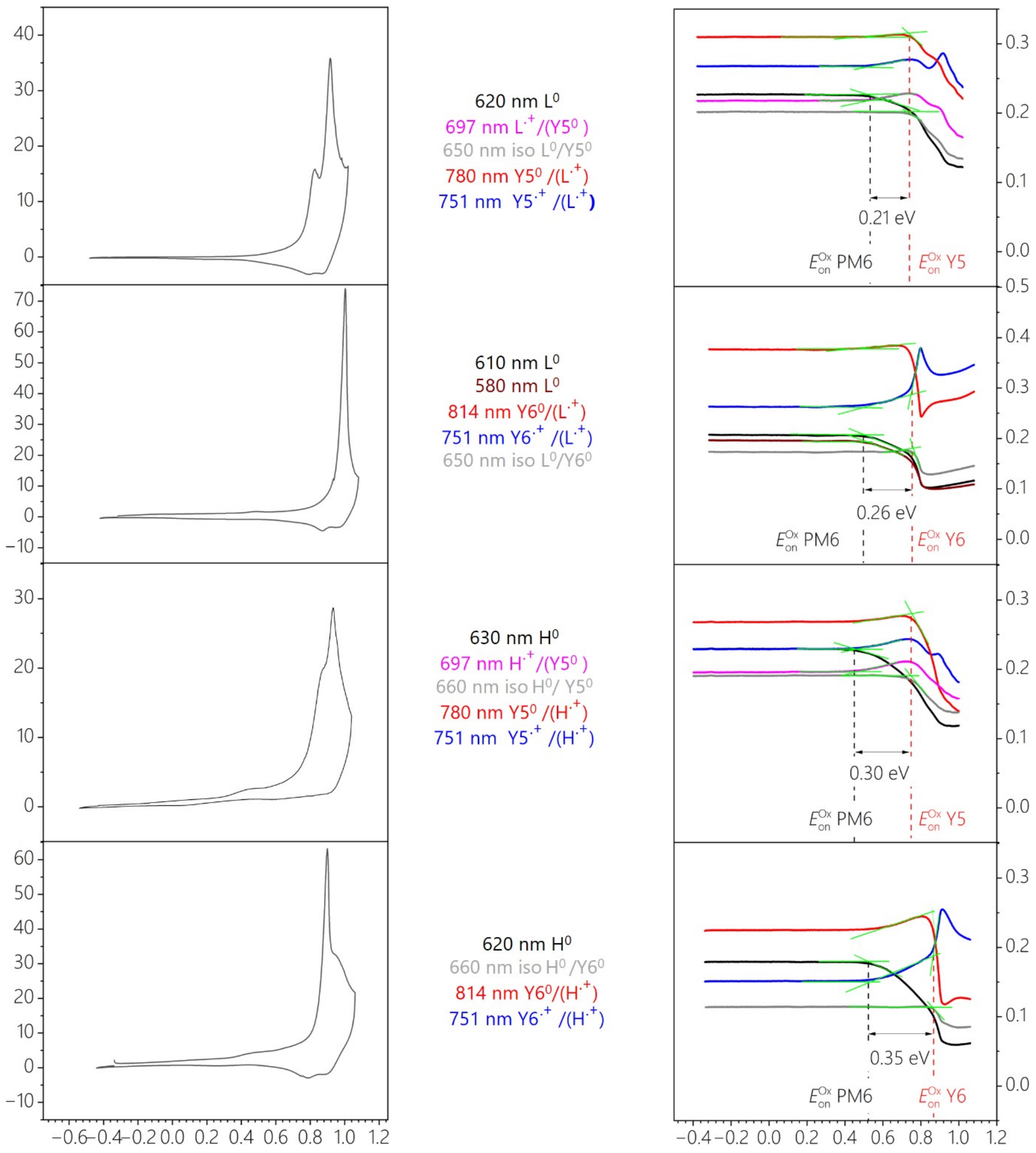

***Figure S6**. (left panels) In-situ CV plots measured on the four model blends: top-to-bottom L:Y5, L:Y6, H:Y5, H:Y6. (right panels) corresponding peak trend for the different blends L:Y5, L:Y6, H:Y5 and H:Y6 (top to down). The spectral evolution of the donor and acceptor band peaks are fitted with the tangent method to obtain the oxidation onsets of the blend constituents as measure within the blend, whose difference yields the ionization energy between the blend constituents.*

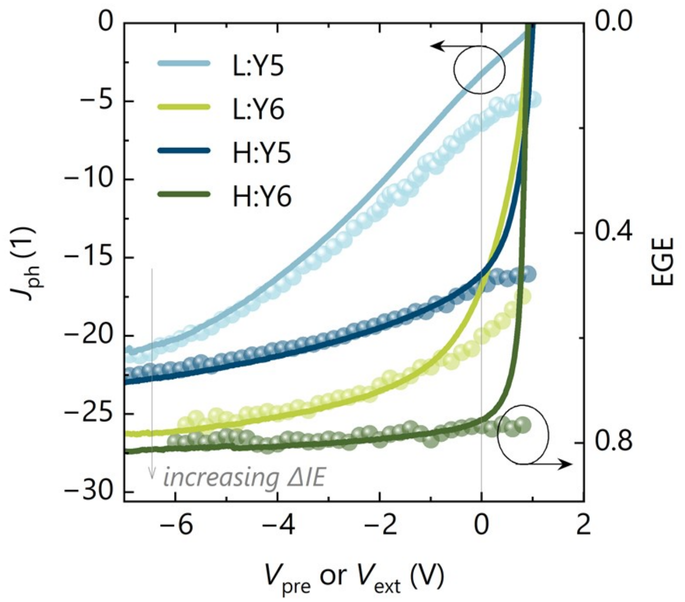

Figure S7. *High reverse bias overlay of photocurrent (J_{ph}) from JV characteristics and external free charge generation efficiency (EGE) from modified time delayed collection field (mTDCF) measurements. The excitation wavelength was 532 nm, the fluence was 50 nJ/cm^2 and the collection bias was -7 to -8 V reverse bias.*

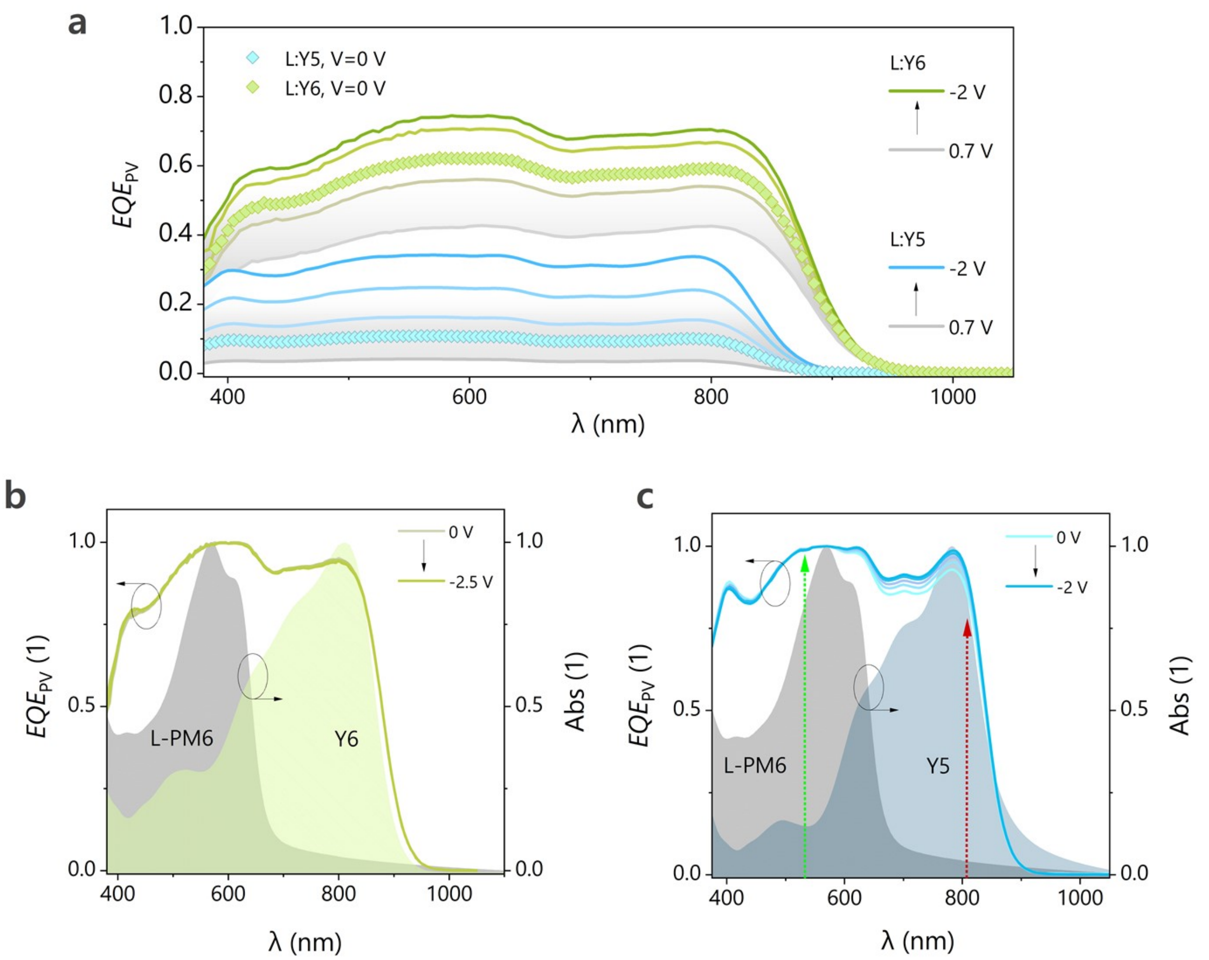

Figure S8. *(a) EQE_{PV} as a function of bias for L:Y6 (in green) and L:Y5 (in blue). (b) Normalized EQE_{PV} as a function of bias for L:Y6, showing that the shape of the spectrum is independent of bias. The right y-axis is the normalised absorption to show the spectral regions of absorption dictated by the donor and acceptor as indicated by the shaded portions. (c) Normalized EQE_{PV} as a function of bias for L:Y5, wherein the marginal enhancement of the Y5-relevant peak can be observed. The vertical coloured arrows indicated the two laser wavelengths used to selectively excite the L:Y5 system in excitation-dependent TDCF measurements, shown in Figure S6.*

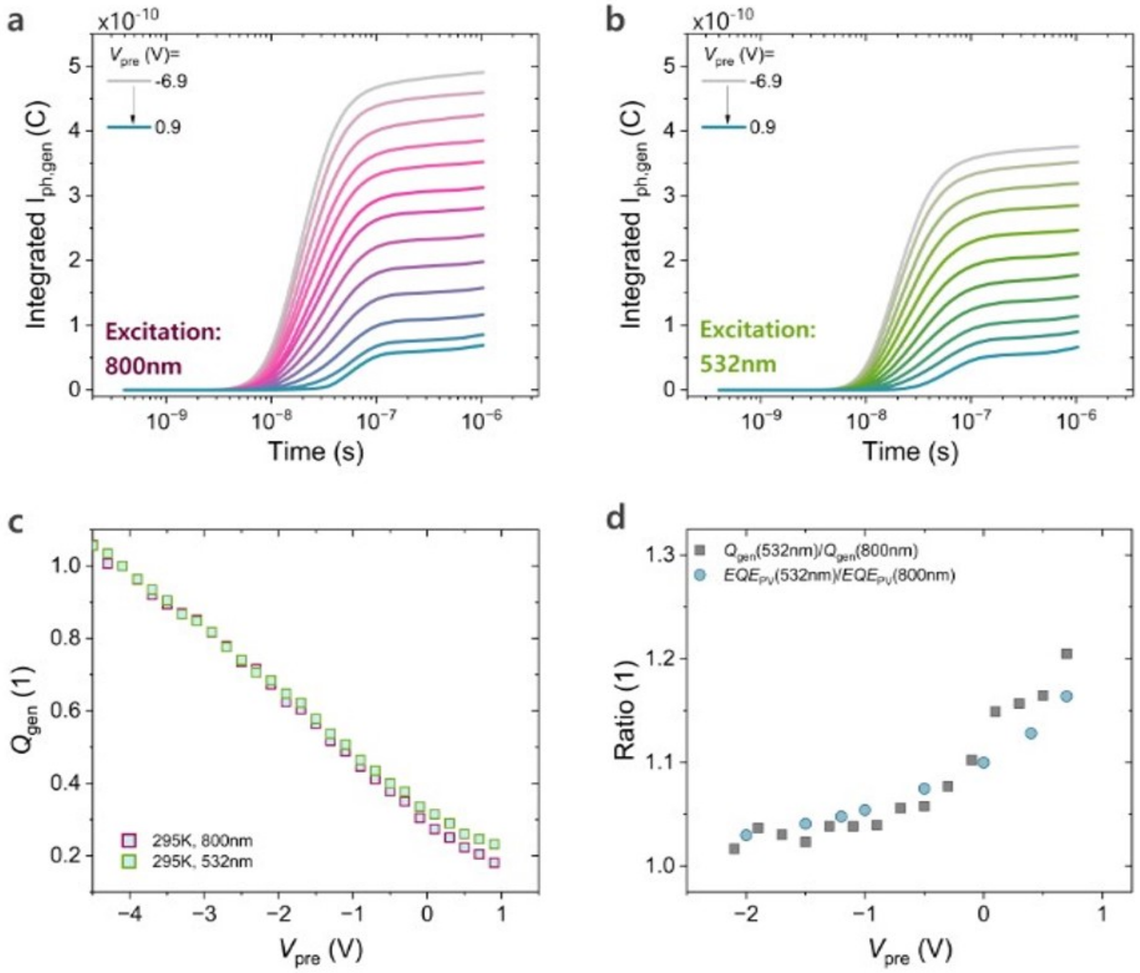

Figure S9. *(a-b) Temporal evolution of generated charge from the integrated transient photocurrent, recorded from TDCF measurements, at different pre-bias conditions (V_{pre}) for the L:Y5 system, for (a) λ=800nm excitation and (b) λ=532nm excitation. Note that in both cases, the extracted charge saturates after ca. 100 ns, meaning that the extraction of the photogenerated charges is efficient even in this low-performing blend (c) Normalized free charge plotted normalised at V_{pre}=-4 V. The bias-dependence of free charge generation is identical for the two selective excitations, with marginal divergence occurring in the more positive voltage range. In this range, excitation of the acceptor yields marginally more field-dependence than for donor excitation, in accordance with EQE_{PV} data, with the difference in bias-dependences being less than 5%. (e) The ratio of free charge as well as the EQE_{PV} for selective excitations, plotted as a function of the applied bias to the device (V_{pre} in case of free charge), showing agreement between the field-dependences recorded from both techniques for the L:Y5 system.*

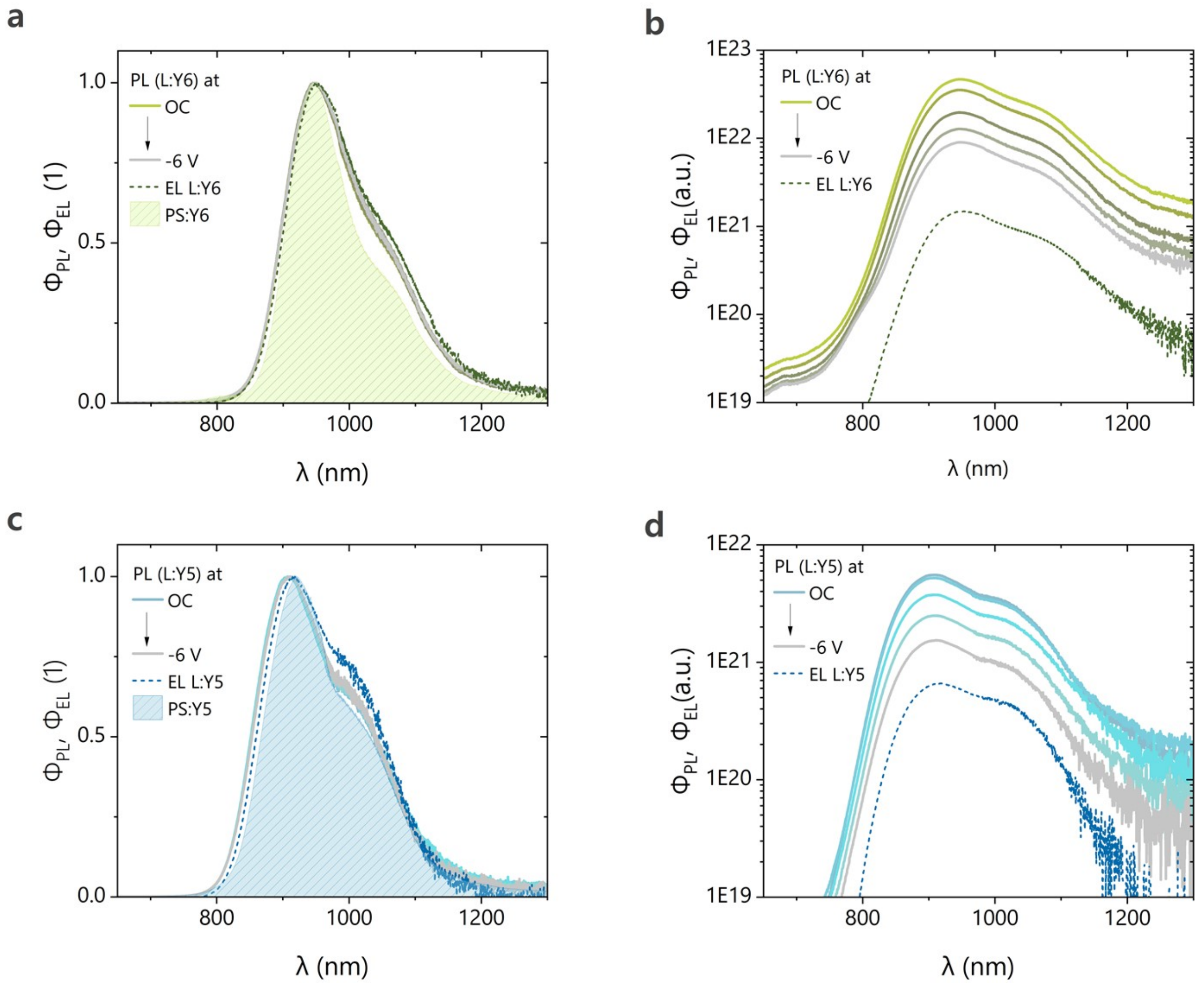

Figure S10. (a) Normalised EL and bias-dependent PL of a L:Y6 blend device, compared to a PL spectrum for a polystyrene:Y6 blend device at OC. (b) EL and bias-dependent PL spectra of L:Y6 plotted on a semi-log scale. (c) The equivalent of subgraph a, but for L:Y5. (d) The equivalent of subgraph b, but for L:Y5. The overlap of all spectra in subgraphs a and c shows that the radiative decay pathway for free charges too occurs via the NFA singlet in these systems, and that the contribution of CT emission in the PL and EL spectra of the device is likely negligible.

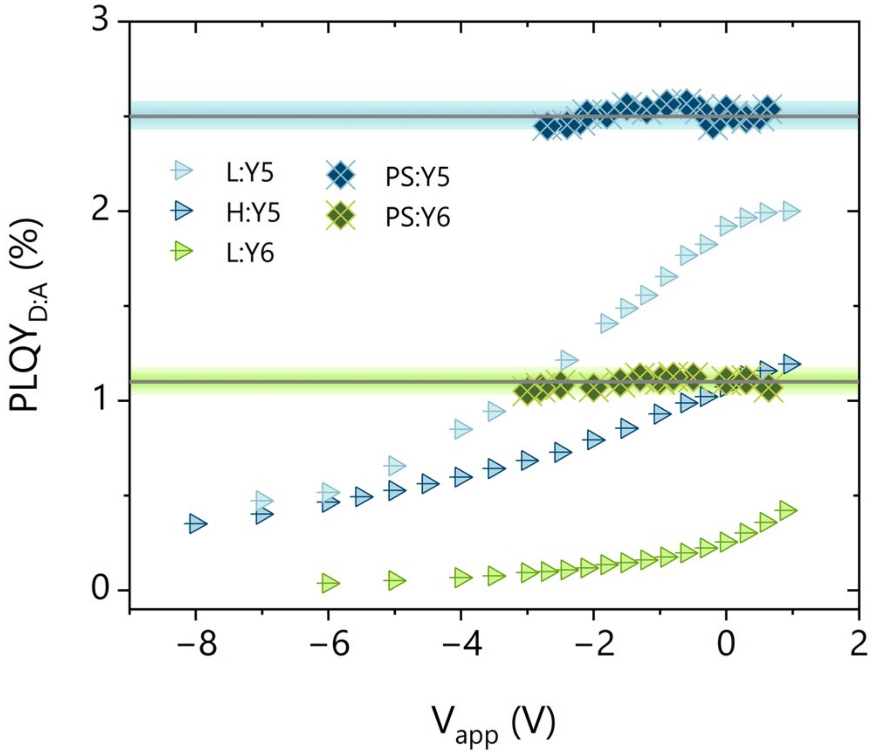

Figure S11. *Experimental PLQY values extrapolated as a function of external bias using the ssPL photon flux, for the inefficient blend systems as well as polystyrene:NFA devices in the same blend ratio (1:1.2). The conversion was done using the equation* $PLQY_{D:A}(V) = \frac{\phi_{PL,max}(V)}{\phi_{PL,max}(V_{OC})} \cdot PLQY_{D:A}(V_{OC})$*. The emission from the blends is clearly bias-dependent, while that of blends of the NFA in a polystyrene matrix are bias-invariant. This suggests that the bias-dependence of emission in the blend is caused by processes which involves both the donor and the acceptor, mostly like the dissociation of the NFA LE into an D:A CT state.*

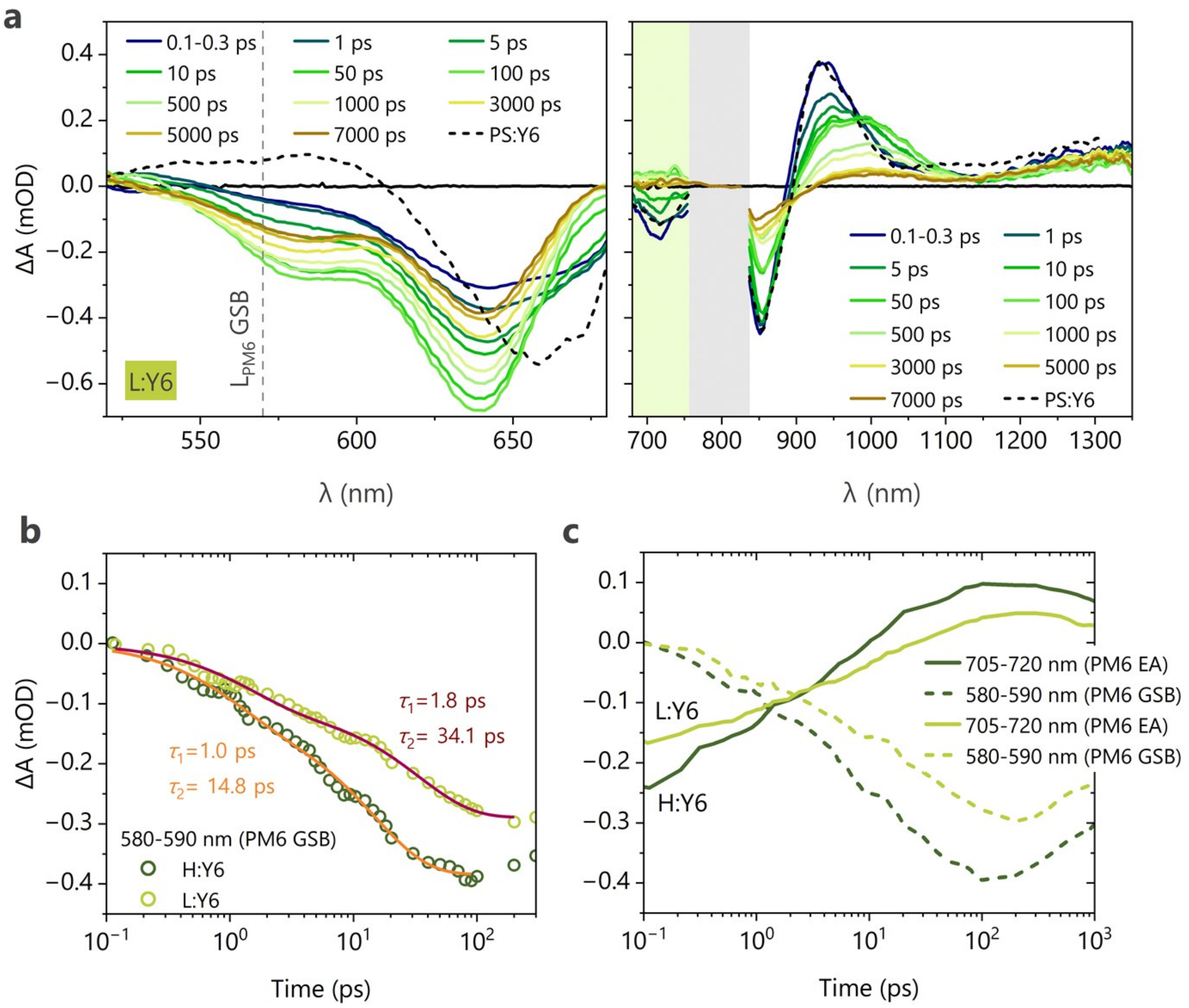

Figure S12. *(a) Transient absorption spectra in the visible and infrared region, probed on an L:Y6 film of excited with a 1.77eV laser pulse of 2 μJ/cm² fluence for preferential NFA excitation. The grey shaded area denotes the wavelength region where the signal becomes strongly disturbed by the 800 nm pump for white light generation. (b) Comparison of the PM6 ground state bleach (GSB) dynamics for H:Y6 and L:Y6 blends, showing a faster rise in the case of H:Y6 with higher ΔIE. (c) System-wise comparison of both PM6 GSB dynamics and PM6 electroabsorption (EA) signals corresponding to charge generation and charge separation.*

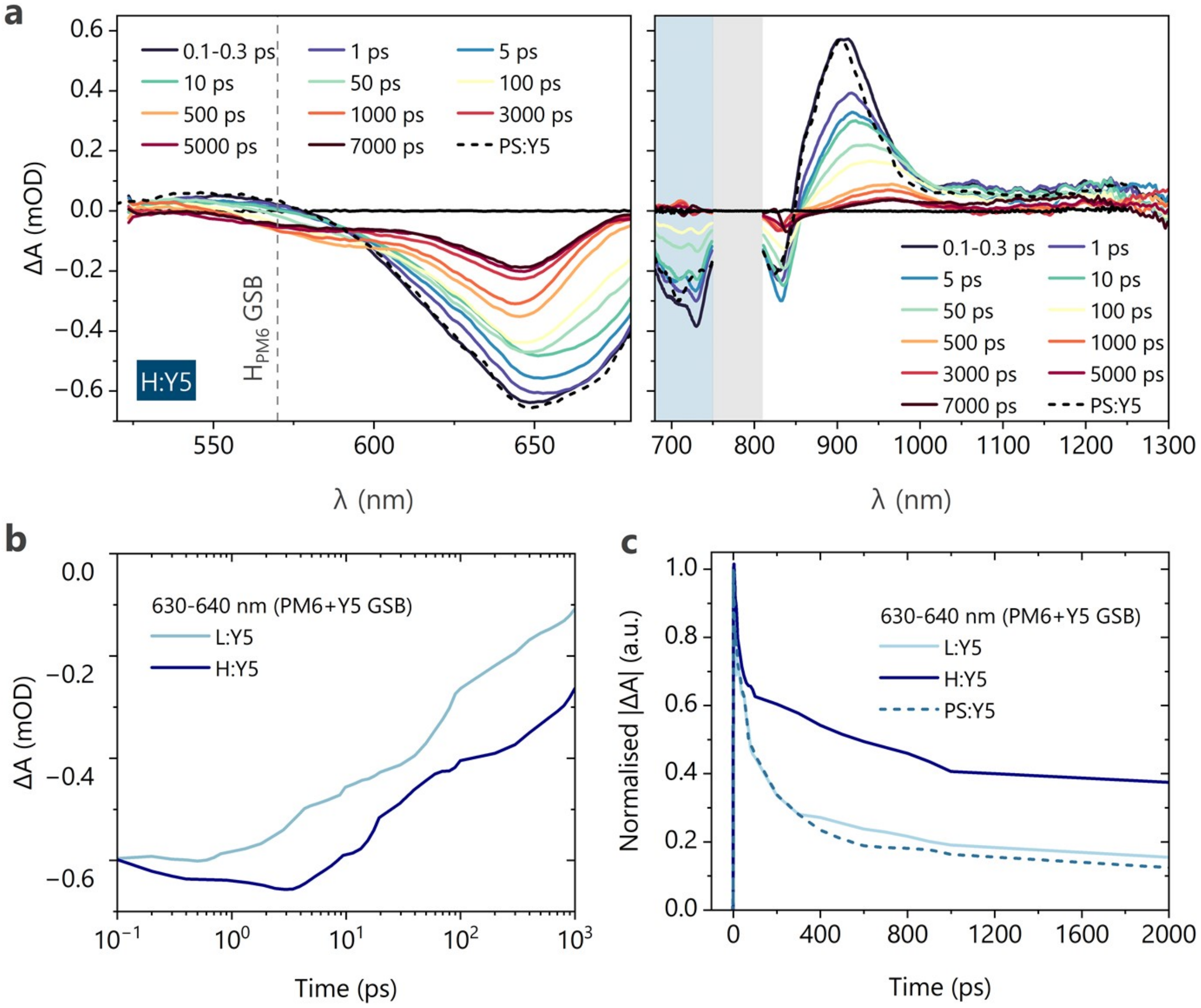

Figure S13. *(a) Transient absorption spectra in the visible and infrared region, probed on an H:Y5 film of excited with a 1.77eV laser pulse of 2 μJ/cm² fluence for preferential NFA excitation. wavelength region where the signal becomes strongly disturbed by the 800 nm pump for white light generation. (b) Comparison of the PM6 and Y5 ground state bleach (GSB) dynamics for H:Y5 and L:Y5 blends, showing a rise in the case of H:Y5 with higher ΔIE. (c) Longer time dynamics of the PM6+Y5 GSB features in Y5-based blends, normalised to the initial intensity of the two blends along with the normalised GSB decay of PS:Y5.*

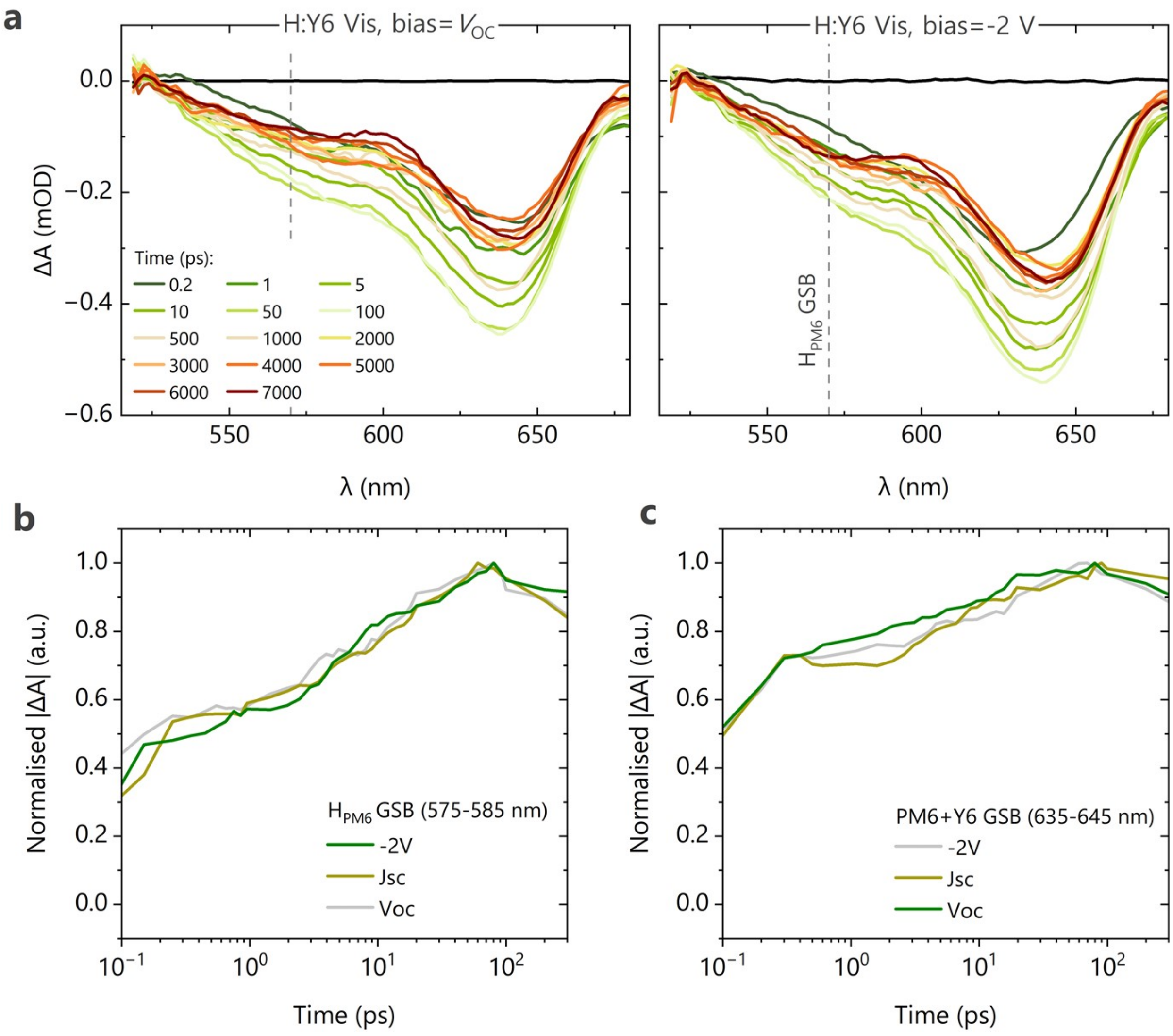

Figure S14. *(a) Bias-dependent transient absorption spectra in the visible region probed on an H:Y5 device, when biased externally with V_{OC} and -2 V. The semi-transparent sample was excited with a 1.77eV laser pulse of 7 μJ/cm² fluence for preferential NFA excitation. (b) Bias-dependent TA dynamics of the PM6 relevant GSB band in H:Y6 devices, showing no alternation of the free charge generation properties with an external field. (c) The equivalent of subgraph b, but of the GSB band around 640 nm relevant to both PM6 and Y6.*

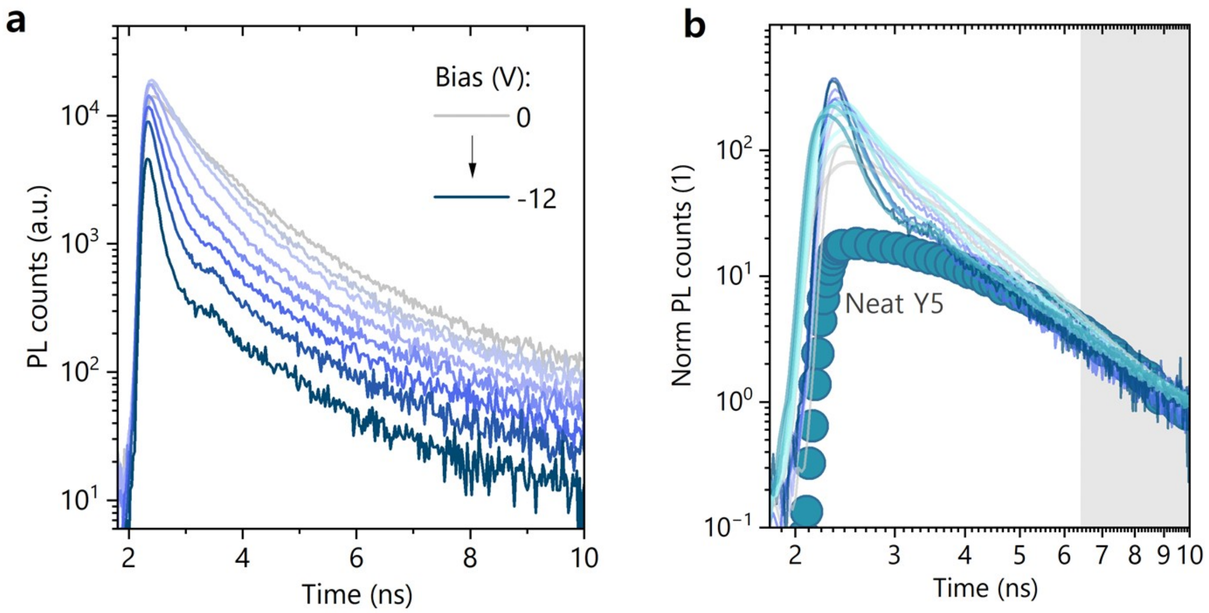

Figure S15. *(a) TrPL decays of a H:Y5 device for different bias. (b) Normalised TrPL kinetics of H:Y5 (dark blue lines) and L:Y5 devices (light blue lines), as a function of external bias. The intensities at each bias are normalised to the respective values at 10 ns. Overlaid on top is the TrPL kinetics of PS:Y5, indicating the emission from undissociated LE excitons in Y5 domains at later times in both blends.*

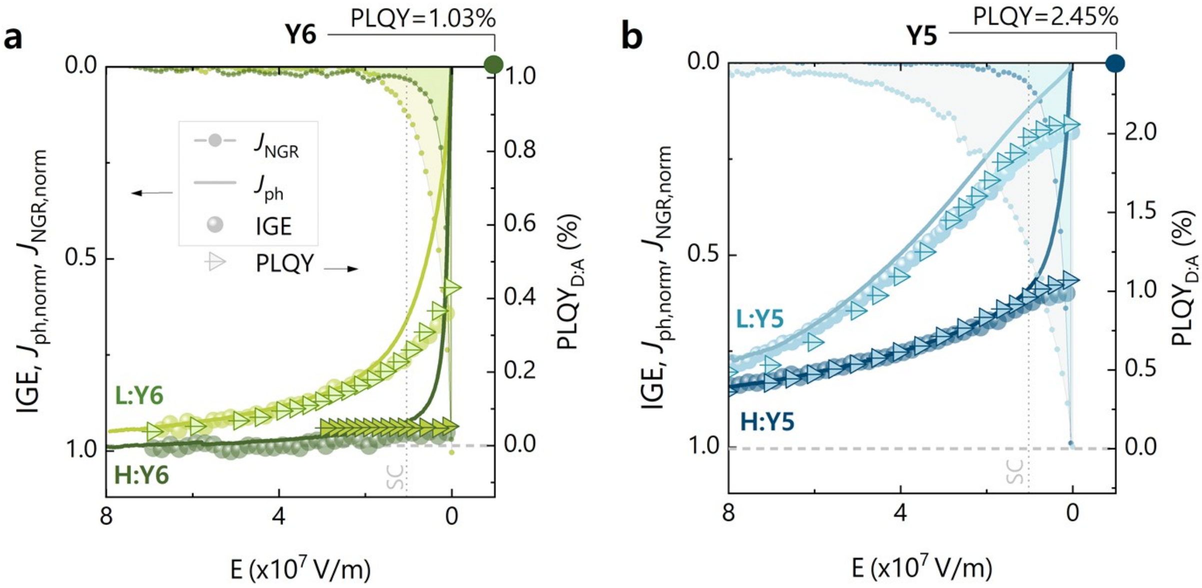

Figure S16. *(a) An overlay of J_{ph}, internal free charge generation efficiency (IGE), non-radiative recombination current (J_{NGR}), and PLQY as a function of the effective electric field across the active layer for the Y6-based model systems. In all cases, J_{ph} was normalised to -27 mA/cm^2 which is the reverse saturation current of the reference PM6:Y6 system. The IGE was obtained as the experimental external free charge generation efficiency, or EGE. The EGE is calculated using the measured photogenerated charge and the incident laser fluence, as the ratio of the generated free charge carriers to the incident photon count. To get IGE, the EGE is normalised to the maximum of 0.8, which accounts for the loss of maximum photogenerated charge from reflection and parasitic absorption losses. The difference between the normalised J_{ph} and IGE is the so-called normalised non-geminate recombination current $J_{NGR,norm}$. Based on the overlay, an excellent anticorrelation of the generation and emission properties is observed across the entire effective field range. (b) The equivalent of subgraph a, but for the Y5-based blends. The vertical dotted line marks the effective electric field at short-circuit conditions. Importantly, the scenario of zero free charge generation (open-circuit) corresponds to emission that equals the PLQY of the respective NFA. Hence, the anticorrelation of emission and generation dictates that the PLQY from the blend at a certain bias be represented as a fraction of the PLQY of the neat NFA, depending on the efficiency of free charge generation at that bias condition.*

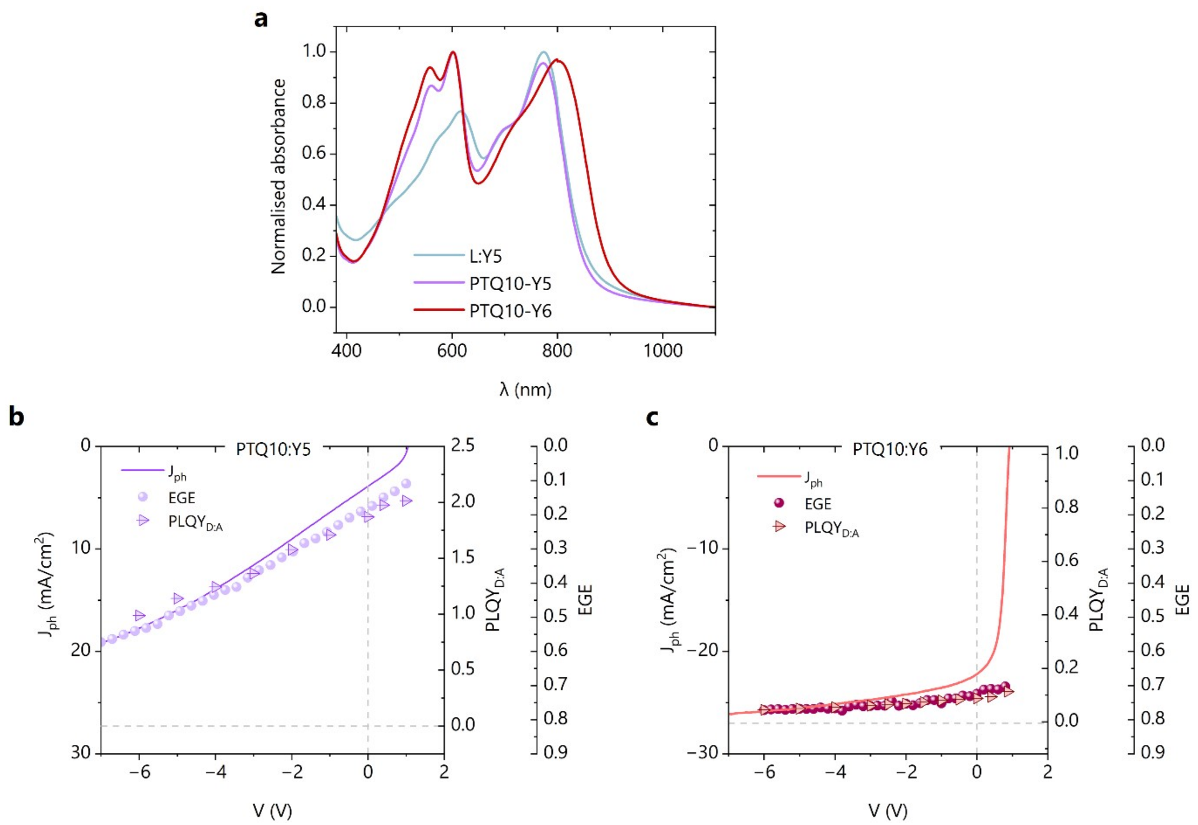

Figure S17. *(a) Normalised absorption spectra for the PTQ10:Yx systems. Overlaid is the normalised absorbance of L:Y5 for comparison. (b-c) An overlay of J_{ph} from JV characteristics, EGE from mTDCF and $PLQY_{D:A}$ from ssPL data plotted for the PTQ10:Y5 and PTQ10:Y6 OSC, respectively.*

	L:Y5	PTQ10:Y6	PTQ10:Y5
Voc (V)	0,99	0,88	1,00
Jsc (mA/cm^2)	3,48	23,1	4,3
FF (%)	27,65	59,54	37
PCE (%)	0,96	12,64	1,91

Table S1. *Photovoltaic parameters for the PTQ10 polymer based OSCs systems, along with the parameters of L:Y5 for comparison.*

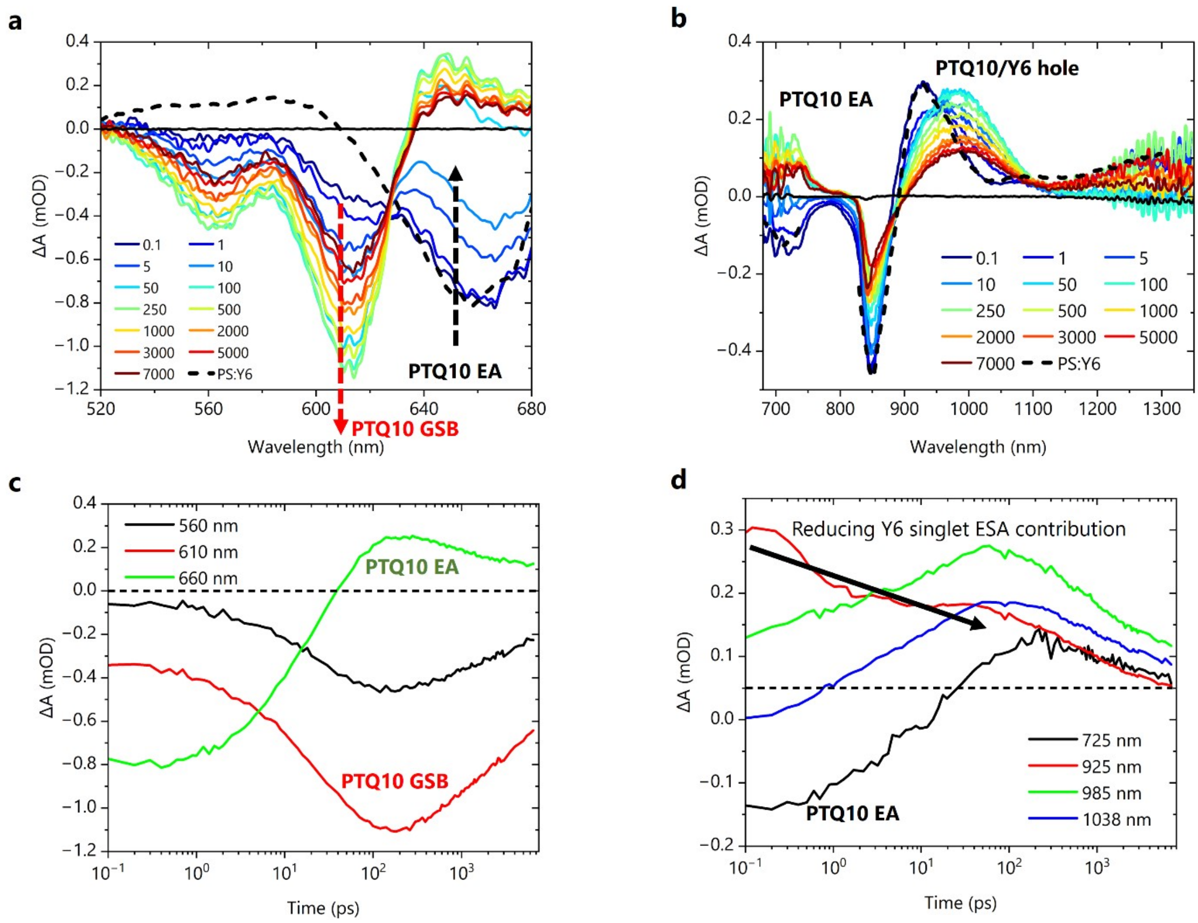

Figure S18. *(a-b) Transient absorption spectra in the visible and infrared regions, probed on a PTQ10:Y6 film excited with a 1.6eV laser pulse of 2 μJ/cm² fluence for preferential NFA excitation. (c-d) Kinetic comparison of TA spectral features in the visible region and NIR region, respectively.*

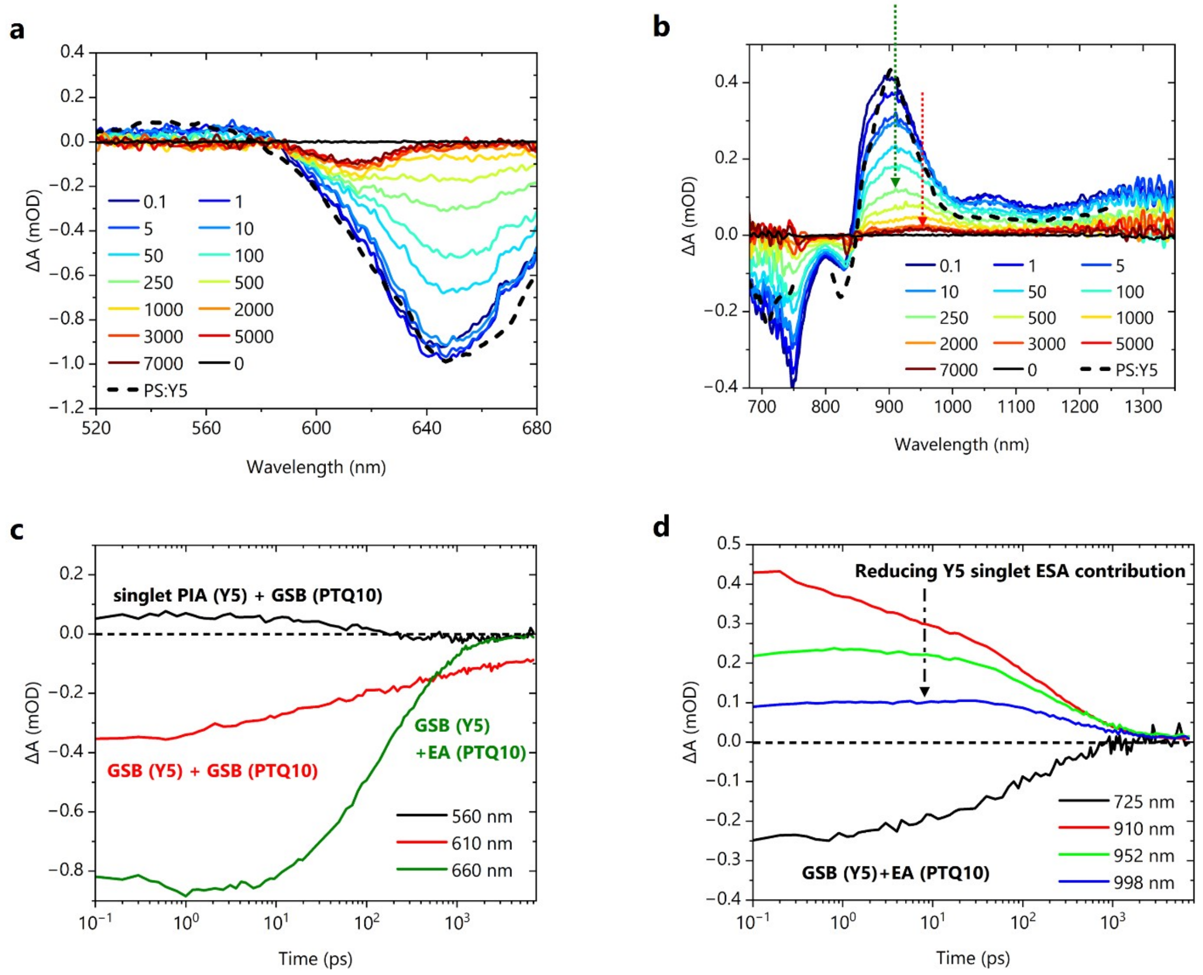

Figure S19. (a-b) Transient absorption spectra in the visible and infrared regions, probed on a PTQ10:Y5 film of excited with a 1.6eV laser pulse of 2 μJ/cm² fluence for preferential NFA excitation. (c-d) Kinetic comparison of TA spectral features in the visible region and NIR region, respectively.

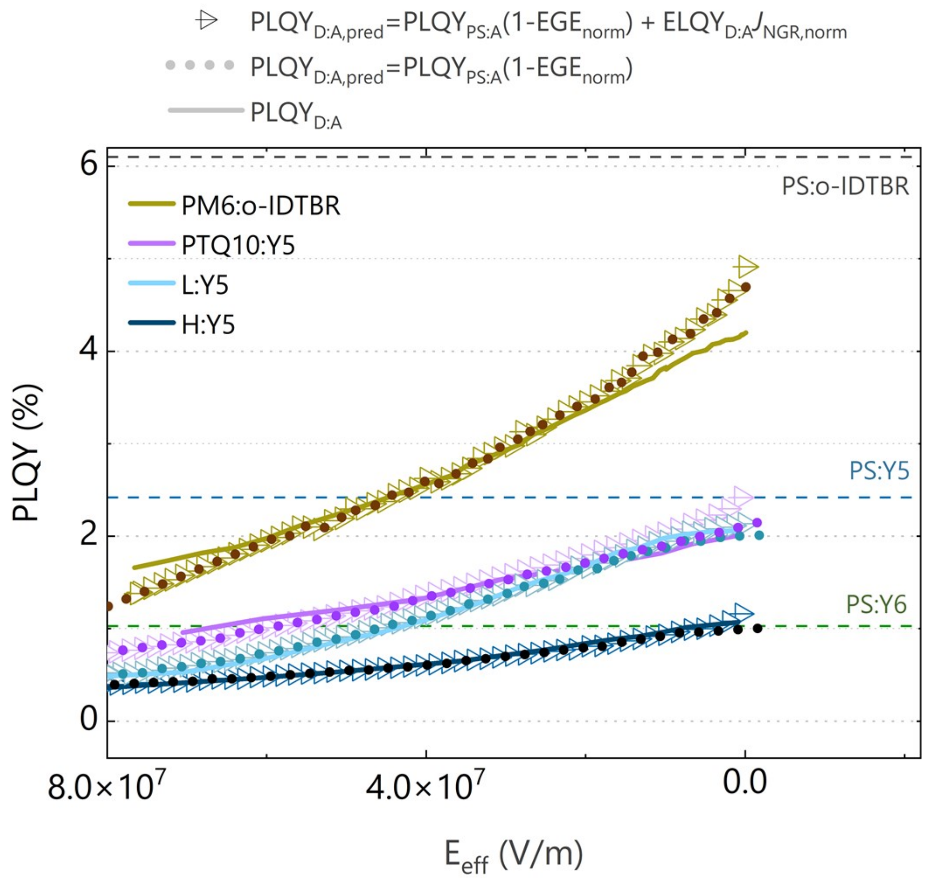

Figure S20. *Overlay and comparison of the field-dependence of the PLQY$_{D:A,pred}$ (triangles) as predicted with equation (2) in the main text from the field dependence of the free charge generation effiicency, and the experimental PLQY$_{D:A}$ (solid lines) obtained from field-dependent ssPL and equation (1) in the main text. In addition, the predicted PLQY$_{D:A,pred}$ without non-geminate recombination component is plotted (dotted). The data show that the field dependence of the measured PLQY$_{D:A}$ is largely due to geminate rather than non-geminate recombination.*

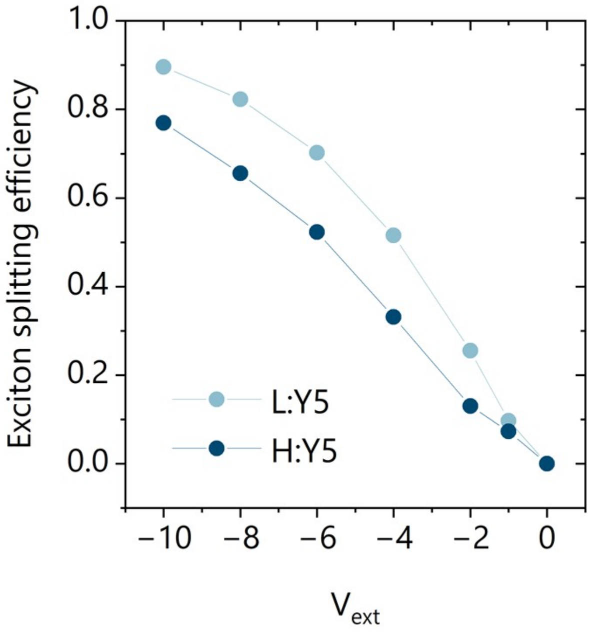

Figure S21. *Bias-dependence of exciton splitting efficiency for the L:Y5 and H:Y5 devices, relative to the emission at 0 V, obtained by integrating the TrPL kinetics of each blend.*

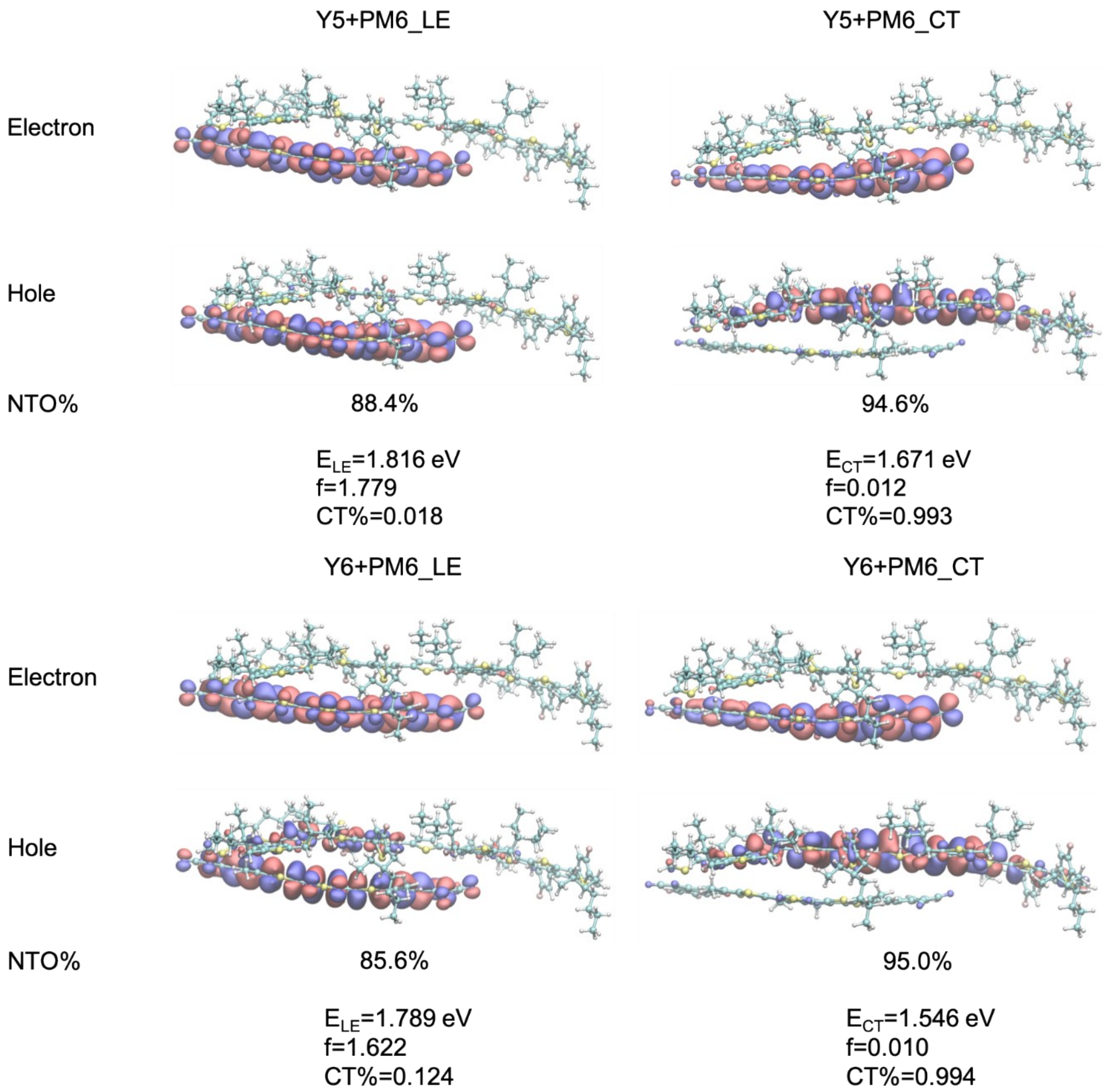

Figure S22. *Natural transition orbitals (hole and electron) of the singlet local excited (LE) and charge transfer (CT) state of the Y5+PM6 and Y6+PM6 system, together with the corresponding weights (NTO%). The excitation energies (E_{LE}/E_{CT}), oscillator strength (f) and weight of CT character are summarized.*

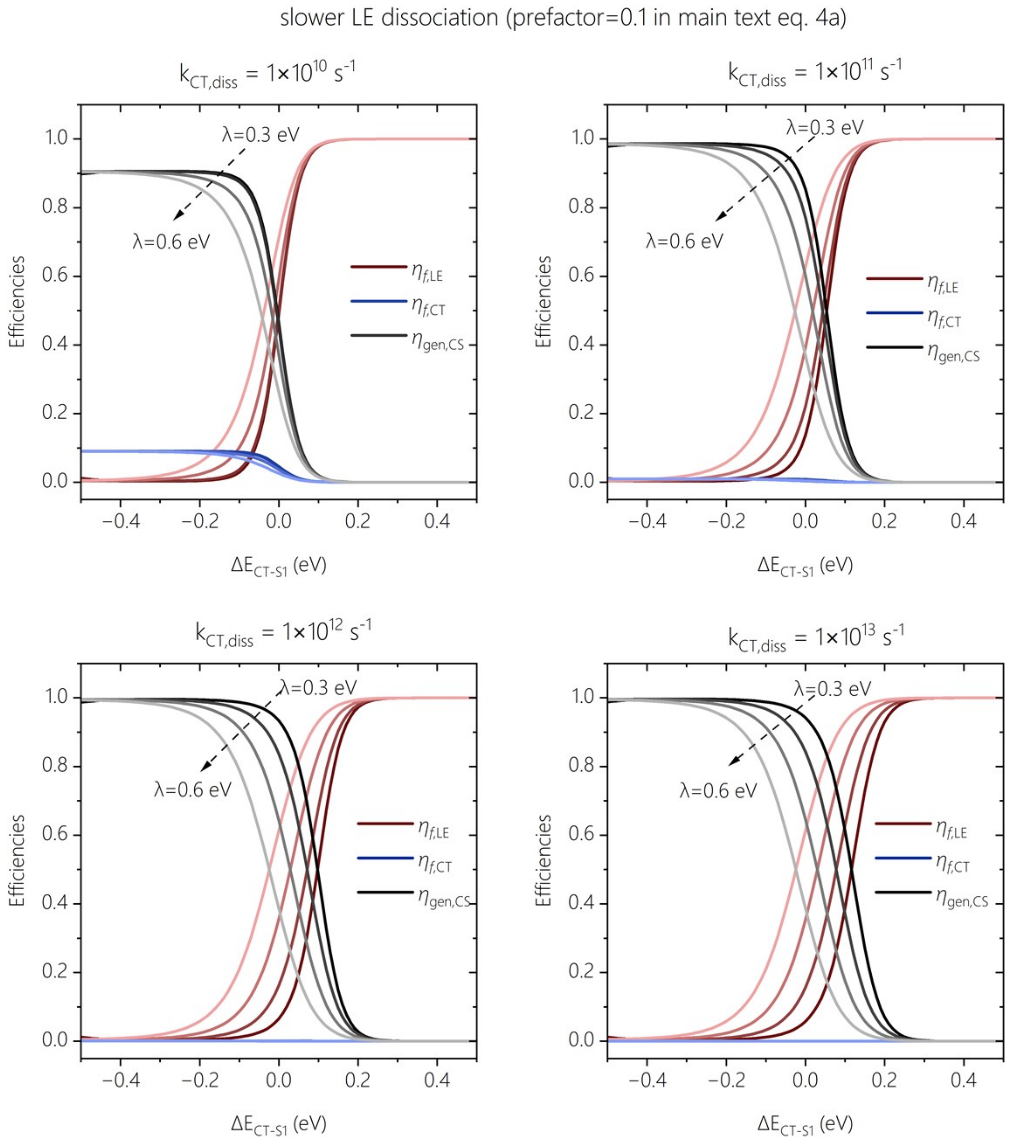

Figure S23. *The dependence of charge generation efficiency, the losses via the decay of the local NFA exciton and the losses due to CT state recombination ($\eta_{gen,CS}$, $\eta_{f,LE}$, $\eta_{f,CT}$, respectively) on ΔE_{CT-S1}. All curves were simulated from the steady-state rate model described in the main text and with a prefactor of 0.1 in Equation 4a(reduced $k_{diss,LE}$). The simulated data is shown for varying reorganisation energies λ, simulated with the parameters in the given table. For all values of $k_{diss,CT}$, when lambda increases, a more negative ΔE_{CT-S1} (CT lower than FE) is needed to achieve efficient free charge generation. This is to ensure that $k_{diss,LE}$ is fast enough to compete with LE decay. Only for rather small $k_{diss,CT}$ does CT recombination contribute to the loss.*

With reduced $k_{diss,LE}$ (prefactor=0.1 in eq.4a in main text)	
Parameter	Value
$k_{f,LE}$ and $k_{f,CT}$	$1x10^{9}$ s^{-1}
$k_{diss,CT}$	$1x10^{10}$ s^{-1} to $1x10^{13}$ s^{-1}
G	$1x10^{28}$ $m^{-3}s^{-1}$
$\|H_{DA}\|$	0.01 eV

λ	0.3, 0.4, 0.5, 0.6 eV

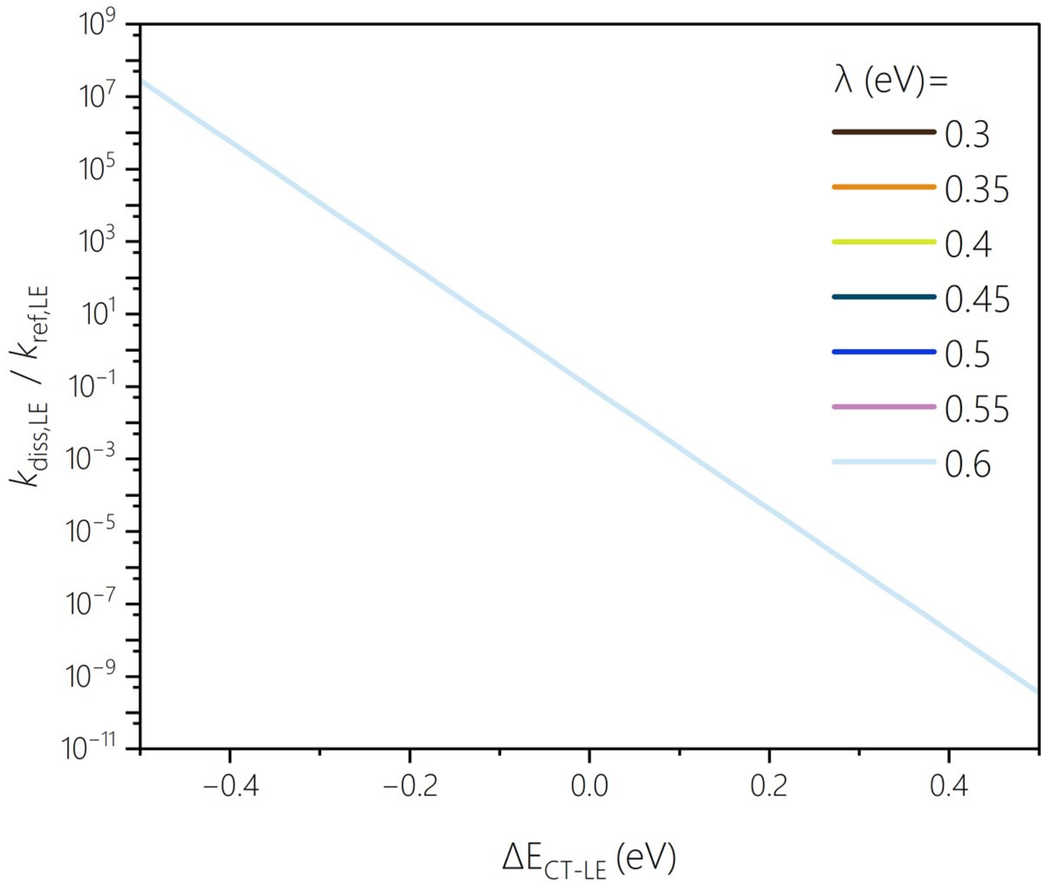

Figure S24. *The ratio of LE dissociation coefficient ($k_{diss,LE}$) to the LE reformation coefficient ($k_{ref,LE}$), taken from Figure 7d of the main text, as a function of the CT-LE offset and for various reorganisation energies. The ratio is completely independent of the reorganisation energy, in consistence with the predictions of detailed balance. Because of this, the value of* λ *affects the individual rates but not the ratio.*

faster LE dissociation (prefactor=1 in main text eq. 4a)

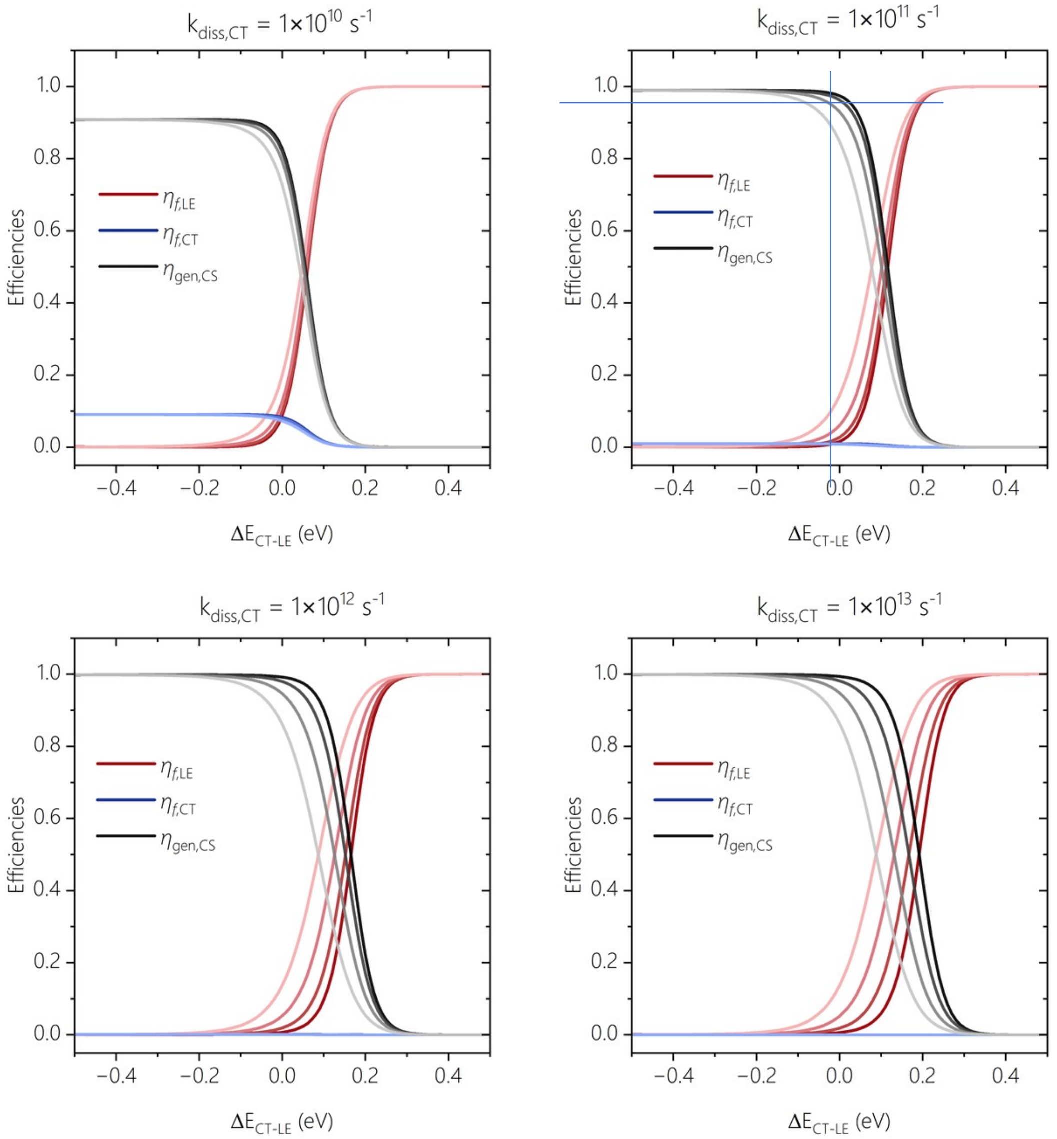

Figure S25. *The dependence of charge generation efficiency, the losses via the decay of the local NFA exciton and the CT state recombination losses ($\eta_{gen,CS}$, $\eta_{f,LE}$, $\eta_{f,CT}$, respectively) on ΔE_{CT-S1}, simulated with our steady-state rate model, now without a reduction in $k_{diss,LE}$. This corresponds to the situation of equal densities of FE and CT states. The simulated data is shown for varying reorganisation energies λ, simulated with the parameters in the given table. As a consequence of faster FE dissociation, free charge generation becomes efficient even for a ΔE_{CT-S1}, provided that the CT dissociation rate $k_{diss,LE}$ is high enough to efficiently compete with CT recombination*

Without reduced $k_{diss,LE}$ (prefactor=1 in eq.4a in main text)

Parameter
$k_{f,LE}$ and $k_{f,CT}$
$k_{diss,CT}$
G
$\|H_{DA}\|$
λ

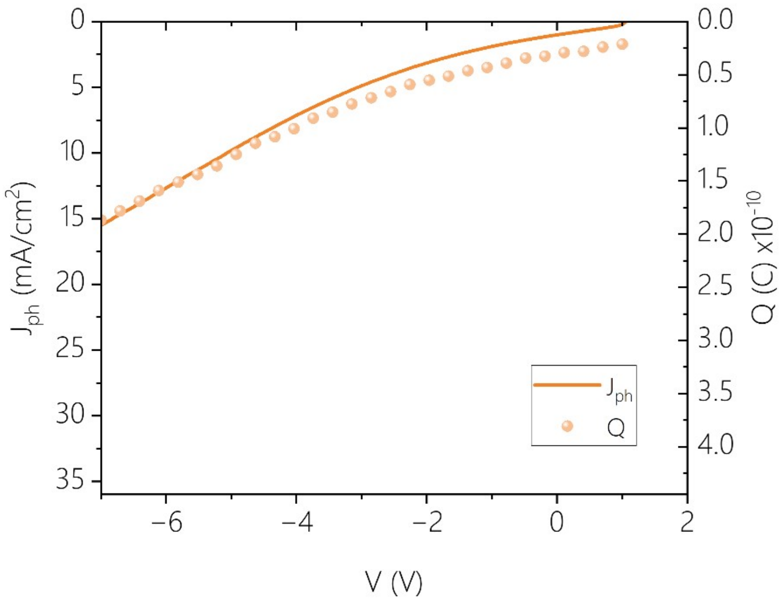

Figure S26. *The dependence of steady state J_{ph} from JV measurements and photogenerated free charge as measured from TDCF for the PTQ10:Y16 system. Both quantities are strongly field-dependent. In the TDCF measurement, the excitation wavelength was 532 nm, the fluence was ca. 50 nJ/cm^2 and the collection bias was -7 to -8 V reverse bias.*

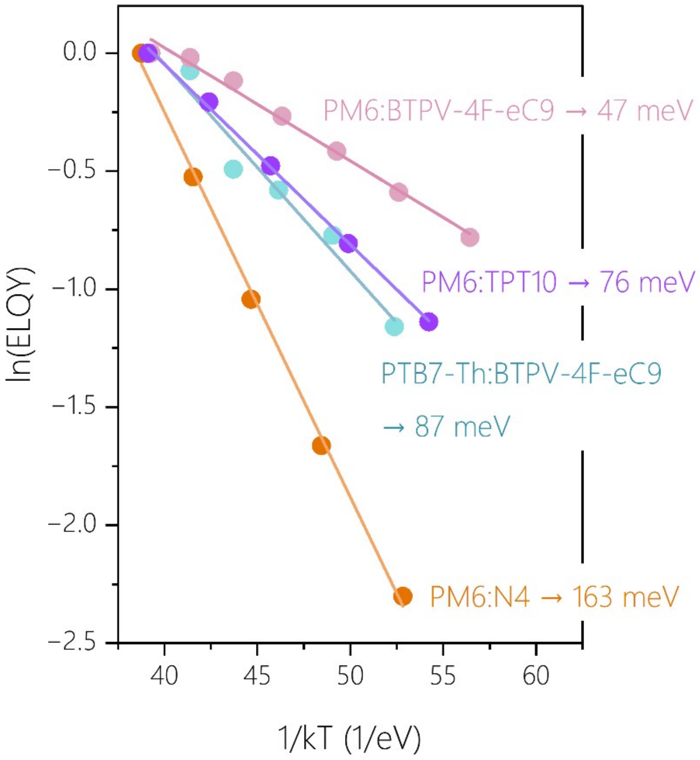

Figure S27. *Temperature-dependent ELQY of additional D:NFA systems which possess varying degrees of field-dependence of free charge generation. The slope gives the activation energy for CT states to emit a photon via the LE state, i.e.* $\Delta E_{LE\text{-}CT}$*. The temperature-dependent ELQY values of PM6:TPT10 are taken from a previous publication*[6].

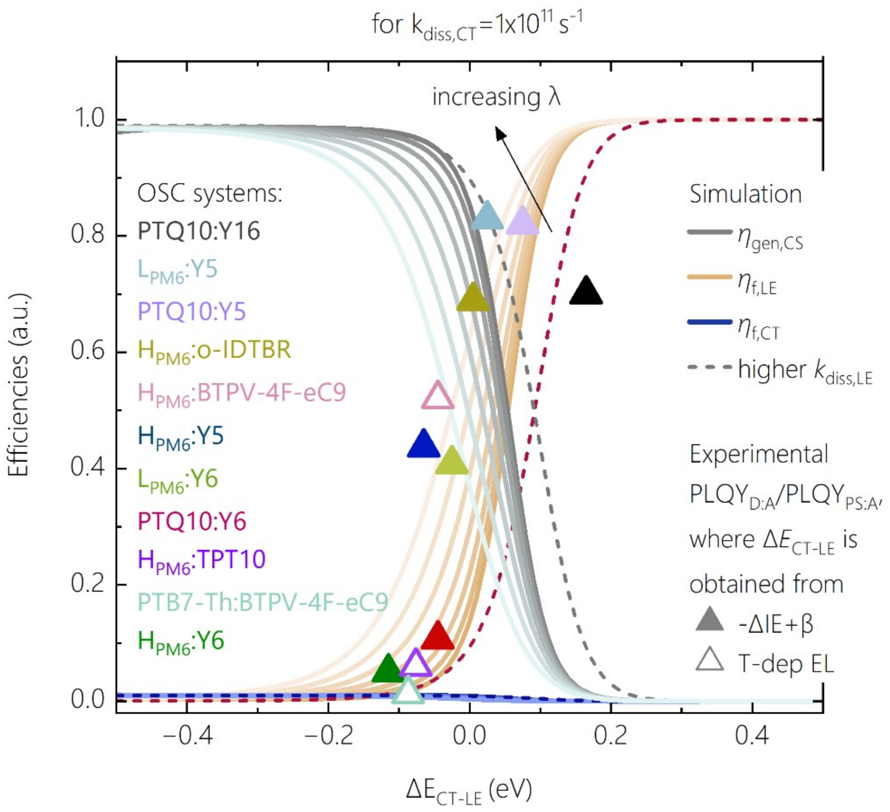

Figure S28. *The dependence of charge generation efficiency, losses via the decay of the local NFA exciton and via the CT state ($\eta_{gen,CS}$, $\eta_{f,LE}$, $\eta_{f,CT}$, respectively) on ΔE_{CT-S1}, simulated at zero-field from the steady-state rate model assuming not all photogenerated excitons are able to undergo charge transfer. The simulated data is shown for varying reorganisation energies λ. Overlaid are the experimental radiative decay efficiencies ($PLQY_{D:A}/PLQY_{PS:NFA}$) of various tested blends measured at open-circuit conditions, as a function of ΔE_{CT-LE}. The dotted lines describe the simulated efficiencies if the singlet dissociation rate were not reduced (i.e., prefactor=1 in equation 4a).*

Supplementary Notes

Supplementary note 1: Organic compounds chemical names

The following organic materials were used as the components in the active layer:

- PM6
  poly[[4,8-bis[5-(2-ethylhexyl)-4-fluoro-2-thienyl]benzo[1,2-*b*:4,5-*b*']dithiophene-2,6-diyl]-2,5-thiophenediyl-[5,7-bis(2-ethylhexyl)-4,8-dioxo-4*H*,8*H*-benzo[1,2-*c*:4,5-*c*']dithiophene-1,3-diyl]-2,5-thiophenediyl]
- Y6
  2,2'-[[12,13-bis(2-ethylhexyl)-12,13-dihydro-3,9-diundecylbisthieno[2'',3'':4',5']thieno-[2',3':4,5]pyrrolo[3,2-*e*:2',3'-*g*][2,1,3]benzothiadiazole-2,10-diyl]bis[methylidyne(5,6-difluoro-3-oxo-1*H*-indene-2,1(3*H*)-diylidene)]]bis[propanedinitrile]
- Y5
  2,2'-[[12,13-bis(2-ethylhexyl)-12,13-dihydro-3,9-diundecylbisthieno[2'',3'':4',5']thieno-[2',3':4,5]pyrrolo[3,2-*e*:2',3'-*g*][2,1,3]benzothiadiazole-2,10-diyl]bis[methylidyne(3-oxo-1*H*-indene-2,1(3*H*)-diylidene)]]bis[propanedinitrile]
- o-IDTBR
  (5*Z*,5'*Z*)-5,5'-((7,7'-(4,4,9,9-tetraoctyl-4,9-dihydro-*s*-indaceno[1,2-*b*:5,6-*b*']dithiophene-2,7-diyl)bis(benzo[*c*][1,2,5]thiadiazole-7,4-diyl))bis(methanylylidene))bis(3-ethyl-2-thioxothiazolidin-4-one)
- PTQ10
  poly[[6,7-difluoro[(2-hexyldecyl)oxy]-5,8-quinoxalinediyl]-2,5-thiophenediyl]]

Supplementary Note 2: Constructing bias-dependent trPL quenching

To construct a bias-dependent TrPL quenching, we use the exciton splitting efficiency $\eta_{\mathrm{LE,split}}$ as follows:

$$Max\ of\ \phi_{TrPL}(V) = (1 - \eta_{LE,split}) \cdot Max\ of\ \phi_{TrPL}(0)$$

$$where\ \eta_{LE,split} = 1 - \frac{\int \phi_{TrPL}(V)}{\int \phi_{TrPL}(0)}$$

Then, the maximum of $\phi_{TrPL}(V)$ is normalised and scaled to (1-IGE), so that the normalised emission extrapolated to zero field equals a generation efficiency of EGE=0. This would be the case if all photogenerated NFA singlet excitons would decay radiatively without contributing to free charge generation.

Supplementary Note 3: Quantum mechanical calculations

The natural transition orbitals (NTOs) of the electrons and holes of the excited states along with the excitation energies (E_{LE}/E_{CT}), oscillator strength (f) and weight of CT character (CT%) of the Yx+PM6 interfacial dimer system are shown in Figure S22, and the excited state energies for all computed interfacial systems (dimer and trimer) are provided below in Table N3.1.

Table N3.1. *Energies of excited state energies (local singlet state, LE, and charge-transfer state, CT) in interfacial dimer (Yx+PM6) and interfacial trimer (2Yx+PM6) systems, used in computational modelling. The difference in LE-CT state energies, as well differences in the energies in Y5- and Y6-based systems are also provided.*

Energy of:	*CT*	*LE*	*CT*	*LE*
Interfacial system:	*2Y5+PM6*	*2Y5+PM6*	*Y5+PM6*	*Y5+PM6*
@ 4.5 Å	1.696	1.790	1.671	1.816
LE-CT for Y5	0.095		0.145	
	CT	*LE*	*CT*	*LE*
	2Y6+PM6	*2Y6+PM6*	*Y6+PM6*	*Y6+PM6*
@ 4.5 Å	1.550	1.770	1.546	1.789
LE-CT for Y6	0.220		0.243	
Y5-Y6	0.146	0.020	0.125	0.027

We evaluated the forward and backward reorganization energies (λ_1, λ_2) of the interface systems for their NE to CT transition were calculated with the following equation[7]:

$\lambda_1 = E_{eA}(Yx)-E_{eE}(Yx)+ E_{nC}(PM6)-E_{nN}(PM6)$,

$\lambda_2 = E_{aE}(Yx)-E_{aA}(Yx)+ E_{cN}(PM6)-E_{cC}(PM6)$,

where the E_{eA} and E_{eE} are the Yx excited state energies at the anionic state and excited state optimized geometries respectively, and the E_{aE} and E_{aA} are the Yx anionic state energies at the excited state and anionic state geometries respectively, the E_{nC} and E_{nN} are the PM6 ground state energies at the cationic state and ground state optimized geometries respectively, and the E_{cN} and E_{cC} are the PM6 cationic state energies at the ground state and cationic state geometries respectively. The inner reorganisation energy λ_i is then obtained as the average of the forward and backward reorganization energies.

The energies used in the calculation of the forward and backward reorganisation energies are provided below in Table N3.2. The units are in Hartree, which can be converted to eV by multiplying with a factor of 27.21.

Table N3.2. *Energies (in Hartree) used in the calculation of forward and backward reorganisation energies, used for determining the inner reorganisation energy necessary for the steady-state rate model in the main text. The inner reorganisation energy λ_i is obtained as the average of the forward and backward reorganization energies.*

States	*Energy (in Hartree)*
PM6	
E_{nC}	-12423.6577196
E_{nN}	-12423.6664638
E_{cN}	-12423.4233529
E_{cC}	-12423.4331599
Y6	
E_{eA}	-4817.93566447
E_{eE}	-4817.93850848
E_{aE}	-4818.12018875
E_{aA}	-4818.12309981
Y5	
E_{eA}	-4420.96453717
E_{eE}	-4420.96721174
E_{aE}	-4421.14262068
E_{aA}	-4421.14537882

Supplementary Note 4: Comparison of Marcus rate calculation with experimental data

Table N4.1: Summarises our survey of energy values of PTQ10 polymer and NFAs Y16 and o-IDTBR as reported in literature. We only consider previous reports wherein the energetics were measured in direct comparison with either PM6 (for the donor) or Y6 (for the NFA). Given the variation in reported energy values due to, for instance, different measurement methods and processing techniques, we average the differences. This average is then used that as a shifting parameter to approximate the IE relative to that of H_{PM6} and Y6 (which we determined from spectroelectrochemistry (SEC)).

PTQ10 vs. PM6 energetics (eV)				
PTQ10	*PM6*	*Delta*	*Method*	*Reference*
5.55	5.69	0.14	CV	Joule 4, 1790–1805, August 19, 2020
5.5	5.55	0.05	CV	Adv. Mater. 2022, 34, 2202575
5.1	5.32	0.22	UPS	Adv. Mater. 2022, 34, 2202575
5.15	5.25	0.1	PES	Adv. Mater. 2022, 34, 2202575
5.50	5.55	0.05	CV	Adv. Mater. 2022, 34, 2108749
		Avg=0.11		
o-IDTBR vs. Y6 energetics (eV)				
o-IDTBR	*Y6*	*Delta*	*Method*	*Reference*
5.6	5.68	0.08	PESA	ACS EnergyLett.2020, 5, 1371−1379
5.51	5.63	0.12	CV	Adv. Energy Mater. 2023, 2300980
5.4	5.67	0.27	CV	Adv. Mater. 2022, 34, 2202575
		Avg=0.16		
Y16 vs. Y6 energetics (eV)				
Y16	*Y6*	*Delta*	*Method*	*Reference*
5.56	5.74	0.18	PESA	J.Mater.Chem.A,2023,11,17581–17593
5.46	5.67	0.23	CV	Adv. Mater. 2022, 34, 2202575
		Avg=0.21		

IE determination from SEC and literature		
	IE from SEC (this work)	*Shifted SEC using average difference from literature*
H_{PM6}	-5.28 eV	-
Y5	-5.55 eV	-
Y6	-5.67 eV	-
PTQ10	-	-5.39 (-5.28 + 0.11)
o-IDTBR	-	-5.51 (-5.67 + 0.16)
Y16	-	-5.46 (-5.67 + 0.21)

Table N4.2: Summarises the ways used to determine $\Delta E_{CT\text{-}LE}$. For low offset blends, $\Delta E_{CT\text{-}LE}$ was calculated from the determined ΔIE values by adding a constant offset β, which accounts for the differences in LE and CT binding energies but also band bending effects, see the main document. For the high offset blends, $\Delta E_{CT\text{-}LE}$ was measured as the activation energy of singlet emission in temperature dependent EL experiments.

**System**	**-ΔIE (eV)**	**β** (accounts for LE,CT binding energies and possible band-bending)	**$\Delta E_{CT\text{-}LE}$ = - ΔIE + β (eV)**	**$\Delta E_{CT\text{-}LE}$ from T-EL**
L:Y5	-0.21	0.235	0.025	
L:Y6	-0.26	0.235	-0.025	
H:Y5	-0.3	0.235	-0.065	
H:Y6	-0.35	0.235	-0.115	-0.115[6]
PTQ10:Y5	-0.16*	0.235	0.075	
PTQ10:Y6	-0.28*	0.235	-0.045	
PTQ10:Y16	-0.07*	0.235	0.165	
PM6:o-IDTBR	-0.23*	0.235	-0.005	
PM6:TPT10				-0.076**
PM6:BTPV-4F-eC9				-0.047**
PTB7-Th:BTPV-4F-eC9				-0.087**
PM6:N4				-0.163**

*The ΔIE for the PTQ10-based and o-IDTBR-based blends were taken from the average difference in relevant donor and acceptor IEs tabulated in Table N4.1.

**For these blends, reliable values and statistics of the IEs were not found in literature. Therefore, the LE-CT offset value was determined directly from temperature-dependent EL, a technique previously reported to evaluate energetic offsets at the D:A interface by inspecting the thermally-activated back-transfer process from CT states to LE states. The data are shown in SI Figure S27.

Supplementary Note 5: Scenarios of differing dissociation rates in the Marcus picture

By setting the time derivative in both rate equations (eqs. 3a and 3b in the main text) to zero and solving for CT population, the $\eta_{gen,CS}$ is calculated using eq. 5a in the main text, producing the following:

$$\eta_{gen,CS} = \frac{k_{diss,LE}k_{diss,CT}}{(k_{diss,LE} + k_{f,LE})(k_{ref,LE} + k_{f,CT} + k_{diss,CT}) - k_{diss,LE}k_{ref,LE}}$$

Test cases:

1) For the case of fast CT dissociation, $k_{ref,LE} + k_{f,CT} + k_{diss,CT} \cong k_{diss,CT}$. Then,

$$\eta_{gen,CS} \cong \frac{k_{diss,LE}k_{diss,CT}}{k_{diss,LE}k_{diss,CT} + k_{f,LE}k_{diss,CT} - k_{diss,LE}k_{ref,LE}}$$

$$\cong \frac{k_{diss,LE}k_{diss,CT}}{k_{diss,LE}k_{diss,CT} + k_{f,LE}k_{diss,CT}} = \frac{k_{diss,LE}}{k_{diss,LE} + k_{f,LE}}$$

The right-hand side of the above equation denotes the LE dissociation efficiency competing with its decay. So $\eta_{gen,CS}$ is determined by $k_{diss,LE}(\Delta E, \lambda)$ and progressively depends on λ, seen in the progression of the sub-figures in SI Figure S23 towards fast CT dissociation.

In other words, $\eta_{gen,CS}$ in the transition region has the same shape as $k_{diss,LE}$. We see this in the Figure 8d of the main text.

2) Slow CT dissociation (or fast LE reformation), so $k_{ref,LE} + k_{f,CT} + k_{diss,CT} \cong k_{ref,LE}$. Then,

$$\eta_{gen,CS} \cong \frac{k_{diss,LE}k_{diss,CT}}{k_{diss,LE}k_{ref,LE} + k_{f,LE}k_{ref,LE} - k_{diss,LE}k_{ref,LE}}$$

$$= \frac{k_{diss,LE}k_{diss,CT}}{k_{f,LE}\ \ k_{ref,LE}} = \frac{k_{diss,CT}\ k_{diss,LE}}{k_{f,LE}\ \ k_{ref},LE}$$

Here, the right-hand side of the equation contains $\frac{k_{diss}}{k_{ref}}$, whose ratio of coefficients is independent of λ (see SI Figure S24) but depends on ΔE. Hence, in the scenario when a slow CT dissociation establishes a kinetic equilibrium between the CT and LE population, the free charge generation efficiency shows a reduced dependence on the reorganisation energy, which we see this in Figure S23a for slow CT dissociation.

3) Fast LE dissociation, so $k_{diss,LE} + k_{f,LE} \cong k_{diss,LE}$. Then,

$$\eta_{gen,CS} \cong \frac{k_{diss,LE} k_{diss,CT}}{k_{diss,LE}\left(k_{ref,LE} + k_{f,CT} + k_{diss,CT}\right) - k_{diss,LE} k_{ref,LE}} \cong \frac{k_{diss,CT}}{k_{f,CT} + k_{diss,CT}}$$

Here, the right-hand side of the equation denotes the CT dissociation efficiency competing with CT decay which has a smaller dependence on the reorganization energy than the previous test cases. We see this in SI Figure S25.

References:

[1] Q. Liu *et al.*, “Narrow electroluminescence linewidths for reduced nonradiative recombination in organic solar cells and near-infrared light-emitting diodes,” *Joule*, vol. 5, no. 9, pp. 2365–2379, 2021, doi: 10.1016/j.joule.2021.06.010.

[2] M. Pranav *et al.*, “Anticorrelated Photoluminescence and Free Charge Generation Proves Field-Assisted Exciton Dissociation in Low-Offset PM6:Y5 Organic Solar Cells,” *APL Mater.*, vol. 11, no. 6, p. 061111, 2023, doi: 10.1063/5.0151580.

[3] E. Sağlamkaya *et al.*, “Self-Doping of the Transport Layers Decreases the Bimolecular Recombination by Reducing Static Disorder,” *Sol. RRL*, p. 2300423, 2023, doi: 10.1002/solr.202300423.

[4] N. Tokmoldin *et al.*, “Elucidating How Low Energy Offset Matters to Performance of Nonfullerene Acceptor-Based Solar Cells,” *ACS Energy Lett.*, pp. 2552–2560, 2023, doi: 10.1021/acsenergylett.3c00572.

[5] W. Liu, B. Lunkenheimer, V. Settels, B. Engels, R. F. Fink, and A. Köhn, “A general ansatz for constructing quasi-diabatic states in electronically excited aggregated systems,” *J. Chem. Phys.*, vol. 143, no. 8, p. 84106, Aug. 2015, doi: 10.1063/1.4929352.

[6] B. Sun *et al.*, “Toward More Efficient Organic Solar Cells: A Detailed Study of Loss Pathway and Its Impact on Overall Device Performance in Low-Offset Organic Solar Cells,” *Adv. Energy Mater.*, p. 2300980, 2023, doi: 10.1002/aenm.202300980.

[7] O. López-Estrada, H. G. Laguna, C. Barrueta-Flores, and C. Amador-Bedolla, “Reassessment of the Four-Point Approach to the Electron-Transfer Marcus-Hush Theory,” *ACS Omega*, vol. 3, no. 2, pp. 2130–2140, 2018, doi: 10.1021/acsomega.7b01425.
